# Degradation of G-Quadruplex-Binding Proteins by G4L-PROTAC via Quaternary Complex Formation

**DOI:** 10.1002/anie.202515045

**Published:** 2025-10-05

**Authors:** Rena Nohara, Yuma Tanaya, Mohammad Jafar Sheikhi, Pratiksha Chaudhary, Grinsun Sharma, Hanbin Mao, Kazuo Nagasawa, Masayuki Tera

**Affiliations:** Department of Biotechnology and Life Science, Tokyo University of Agriculture and Technology, 2-24-16 Naka-cho, Koganei, Tokyo 184-8588, Japan; Department of Biotechnology and Life Science, Tokyo University of Agriculture and Technology, 2-24-16 Naka-cho, Koganei, Tokyo 184-8588, Japan; Department of Biotechnology and Life Science, Tokyo University of Agriculture and Technology, 2-24-16 Naka-cho, Koganei, Tokyo 184-8588, Japan; Department of Chemistry and Biochemistry, Kent State University, Kent, OH 44242, USA; School of Biomedical Sciences, Kent State University, Kent, OH 44242, USA; Department of Chemistry and Biochemistry, Kent State University, Kent, OH 44242, USA; School of Biomedical Sciences, Kent State University, Kent, OH 44242, USA; Advanced Materials and Liquid Crystal Institute, Kent State University, Kent, OH 44242, USA; Department of Biotechnology and Life Science, Tokyo University of Agriculture and Technology, 2-24-16 Naka-cho, Koganei, Tokyo 184-8588, Japan; Department of Biotechnology and Life Science, Tokyo University of Agriculture and Technology, 2-24-16 Naka-cho, Koganei, Tokyo 184-8588, Japan

**Keywords:** G-Quadruplex-binding protein, G-Quadruplex ligands, G-Quadruplexes, Proteolysis targeting chimera (PROTAC)

## Abstract

G-Quadruplexes (G4s) are noncanonical nucleic acid secondary structures enriched in genomic regions critical for transcription and replication. These dynamic scaffolds recruit G4-binding proteins (G4BPs), thereby regulating diverse cellular processes. However, the functional roles of G4BPs in the G4-bound state remain poorly defined. Here, we report the development of G4L-PROTACs—bifunctional small molecules that couple a G4 ligand with an E3 ligase recruiter to achieve selective proteasomal degradation of G4-bound G4BPs. Unlike RNAi or CRISPR-Cas9, which eliminate proteins irrespective of binding state, G4L-PROTACs enable depletion of G4BPs only when associated with G4s. Using model G4 motifs from telomeres and the *NRAS* 5′ UTR, we demonstrated in vitro ternary complex formation. In cells, G4L-PROTAC treatment reduced endogenous levels of the G4-resolving helicase DHX36, resulting in a marked increase in intracellular G4 abundance, as shown by BG4 immunofluorescence. This phenotype highlights the ability of G4L-PROTACs to modulate the G4–protein equilibrium in living cells. Notably, G4L-PROTACs do not induce G4-mediated transcriptional silencing, underscoring their precision in modulating nucleic acid–protein interactions. This strategy offers a powerful platform for probing G4–G4BP functions and holds promise for therapeutic targeting of G4-associated proteins.

## Introduction

G-Quadruplexes (G4s) are noncanonical nucleic acid secondary structures formed in guanine-rich sequences. These structures are prevalent in key genomic regions, including promoters,^[1,2]^ telomeres,^[3,4]^ and 5′ untranslated regions (5′ UTRs),^[ 5,6]^ where they regulate essential cellular processes such as transcription, replication, and translation.^[7]^ Historically, G4s were thought to act primarily as static structural barriers, obstructing transcription and translation through steric hindrance.^[8]^ For instance, G4s in the *MYC* promoter were shown to inhibit transcription by RNA polymerase,^[9]^ while RNA G4s in the *NRAS* 5′ UTR hindered ribosomal elongation.^[10]^ This perspective drove significant efforts to develop ligands that stabilize G4 structures, and over a thousand G4 ligands have been reported to date.^[11,12]^

However, recent findings have reshaped our understanding of G4s. Rather than functioning solely as passive barriers, G4s are now recognized as dynamic scaffolds that actively recruit G4-binding proteins (G4BPs) to modulate biological processes.^[13–17]^ For instance, G4s in the *MYC* promoter facilitate transcription by interacting with transcription factors, histone-modifying enzymes, and so forth.^[15]^ Similarly, RNA G4s regulate translation through interactions with proteins like DHX36, which resolves G4 structures.^[18]^ G4s are involved in the elongation of the lifetime of mRNA.^[19]^ These discoveries emphasize the importance of G4–G4BP interactions in cellular biology, suggesting that G4s play active roles in regulating gene expression and other critical processes.^[20]^

The development of G4 ligands has further highlighted the dynamic nature of G4–G4BP interactions. Recent studies demonstrate that G4 ligands can influence these interactions by stabilizing G4 structures or inducing subtle conformational changes.^[21]^ For example, we previously showed that the G4 ligand 6OTD induces flipping out of specific nucleobases in G4, as evidenced by altered S1 nuclease sensitivity.^[22]^ These changes modulate G4–G4BP interactions, emphasizing the need to understand how ligands affect G4 biology. Such findings suggest that optimizing G4 ligands requires a detailed understanding of their impact on G4–G4BP dynamics. Thus, studying these interactions is important not only for advancing G4 biology but also for leveraging G4 ligands for therapeutic applications.

**Figure 1.**
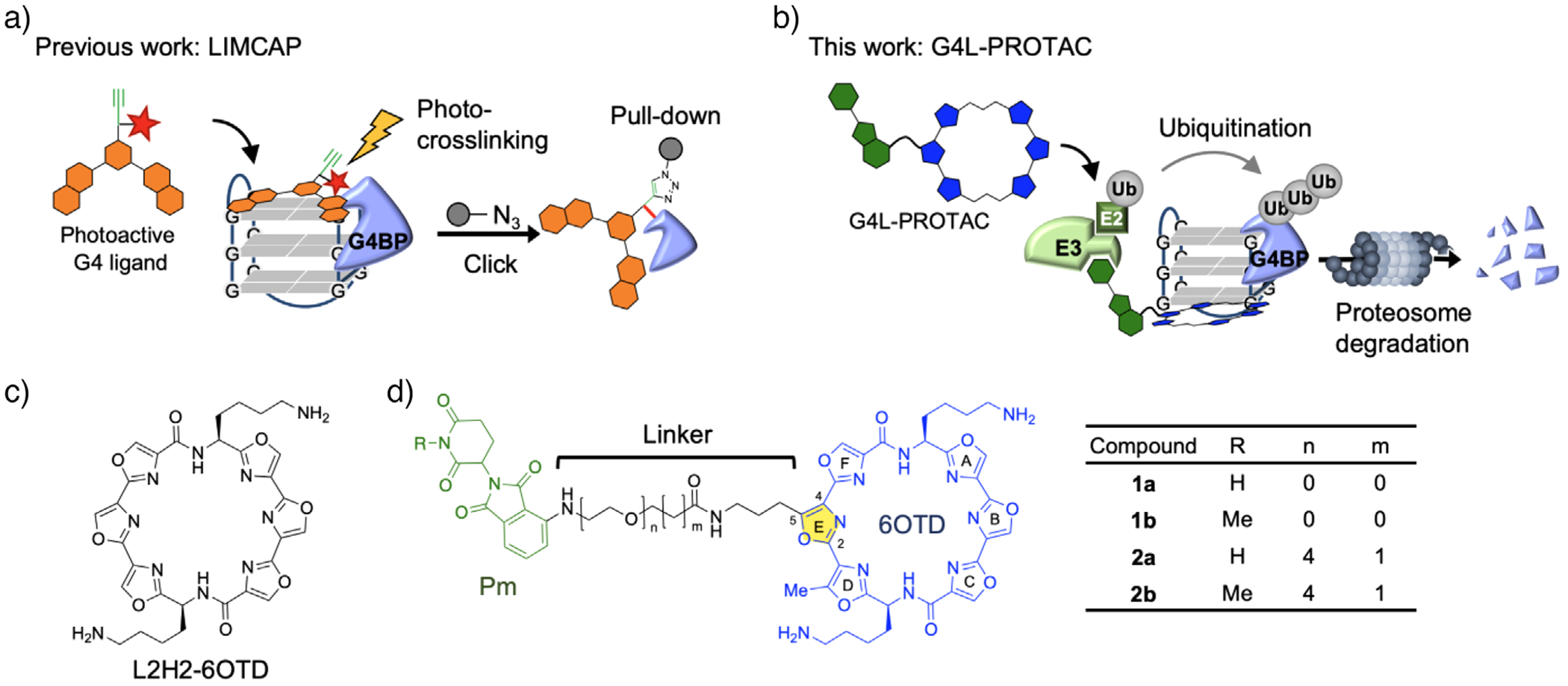
Design of G4L-PROTAC. a) Pulldown of G4BP. G4BP was crosslinked using a G4 ligand containing a photocrosslinking group and an alkyne group, followed by pulldown using azide-modified beads. b) In our approach, G4BP was targeted for proteasomal degradation using a G4 ligand conjugated with a cereblon ligand. c) and d) Chemical structure of L2H2-6OTD (c) and G4L-PROTACs (d).

Despite advances in identifying G4BPs, traditional methods such as pull-down assays and proximity labeling face limitations.^[ 23–25]^ Pull-down assays using intracellular extracts rely on synthetic oligonucleotides,^[26]^ which may not fully replicate cellular environments, while proximity labeling methods such as photocrosslinking can interfere with native G4–G4BP interactions or cause DNA damage and non-specifically crosslink proteins to nucleic acids, as known from ChIP-seq and related techniques.^[27]^ Recently developed techniques, such as G-quadruplex ligand-mediated cross-linking and pulldown (G4-LIMCAP, Figure 1a),^[28]^ have enabled the identification of hundreds of G4BPs under chromatin-like conditions in living cells. Utilizing these technologies, the individual roles of G4s and G4BPs in cellular processes are increasingly understood.

However, the functional consequences of G4–G4BP interactions remain largely unexplored, highlighting a critical gap in our understanding of their biological roles. To address these gaps, elucidating the functional roles of G4–G4BP interactions will require precise experimental approaches, including labor-intensive knockout studies. Oligonucleotide-based PROteolysis TArgeting Chimeras (ODN-PROTACs) represent a promising alternative for targeting nucleic acid-binding proteins.^[29–32]^ For example, a G4 oligonucleotide-conjugated PROTAC using a synthetic G4 motif to induce the selective degradation of RHAU/DHX36 has proved useful for probing G4-protein networks.^[33,34]^ However, ODN-PROTACs rely on introducing exogenous oligonucleotides at concentrations far exceeding physiological levels, raising concerns about their biological relevance. To overcome these limitations, our strategy employs a small-molecule G4 ligand as a warhead, enabling the targeted degradation of endogenous G4BPs bound to native G4 structures. This design is expected to extend the applicability of G4L-PROTACs to diverse G4-mediated cellular processes.

We hypothesized that small-molecule G4L-PROTACs can selectively degrade G4-bound G4BPs while preserving their unbound counterparts (Figure 1b). By facilitating the formation of a ternary complex between an E3 ubiquitin ligase and a G4BP, this approach should enable the precise degradation of G4BPs under physiological conditions, thereby bridging the gap between structural and functional studies of G4s and providing a tool to uncover the molecular mechanisms underlying G4–G4BP interactions and establish a foundation for developing therapeutic applications targeting G4-associated diseases. In this study, therefore, we developed a G4L-PROTAC designed to selectively target endogenous G4BPs bound to naturally occurring G4 structures.

## Results and Discussion

### Design and Synthesis of G4L-PROTAC

To enable the targeted degradation of G4-binding proteins, we designed a molecular glue capable of bridging G4 structures with an E3 ubiquitin ligase. This glue is based on L2H2-6OTD (Figure 1c), a hexaoxazole macrocycle with high specificity and stabilizing effects on G4 structures.^[ 35]^ Structural studies have demonstrated that L2H2-6OTD binds stably to G4 not only with the phosphate backbone via cation–anion interactions but also through *π*–*π* interaction while maintaining excellent solubility under physiological conditions.^[36]^ To preserve its G4-binding properties, an azidopropyl group was introduced at the 5-position of the E ring in 6OTD, a site shown to not interfere with G-quartet binding.^[37,38]^ The resulting compound, L2H2-6OTD-Az, served as the G4-recognition domain for the G4L-PROTAC.

Next, L2H2-6OTD-Az was conjugated with pomalidomide (Pm), a ligand for the E3 ubiquitin ligase cereblon (CRBN). Pomalidomide was chosen not only for its established CRBN-binding capability but also for its intrinsic fluorescence,^[39]^ which facilitates the determination of dissociation constants between G4 structures and the G4L-PROTAC ligands. Assuming that linker length would significantly influence PROTAC activity, we synthesized two derivatives with either a short or a long linker (Figure 1d).^[40]^ To evaluate the specificity of CRBN binding, negative control compounds (**1b** and **2b**) were also synthesized by incorporating an *N*-methylated derivative of Pm, which lacks CRBN-binding activity. The synthesis of compounds **1a** through **2b** involved reducing the azido group of L2H2-6OTD-Az to primary amine, followed by amide coupling with pomalidomide derivatives pre-modified with a carboxylic acid unit (Scheme S1).

### Interaction of G4L-PROTACs with G4 Structures

**Figure 2.**
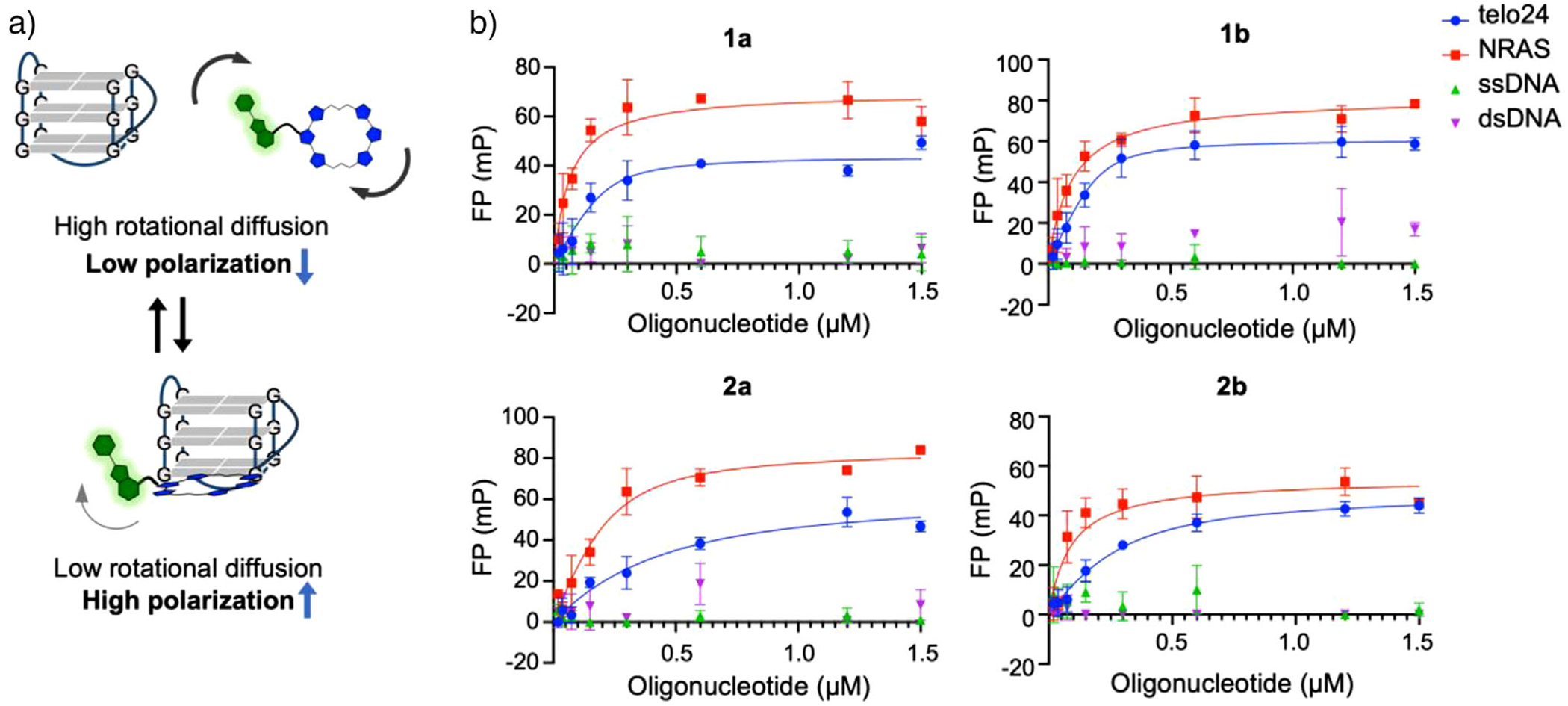
Functional evaluation of G4L-PROTACs. a) Schematic illustration of FP analysis. b) Dissociation constants of G4L-PROTACs for G4 (telo24 and NRAS) and non-G4 (ssDNA and dsDNA). Data represents means ± SD (*n* = 3).

To evaluate whether the introduction of Pm affects G4 binding, we utilized the intrinsic fluorescence of Pm in fluorescence polarization (FP) assays (Figure 2a).^[41]^ Based on the increase of molecular weight of **1a**–**2b** upon the binding with oligonucleotides, the bound/free ratio was calculated from the increment of fluorescence polarization to determine the binding affinities of compounds **1a**–**2b** to DNA or RNA G4 structures. All compounds could bind to both DNA G4 and RNA G4 structures, with dissociation constants (*K*_d_) for telo24 DNA G4 (hybrid-type topology) ranging from 33 nM to 356 nM and those for *NRAS* RNA (parallel-type topology) G4 ranging from 10 nM to 96 nM (Figure 2b and Table 1). Compounds with shorter linkers, **1a** and **1b**, showed slightly stronger binding to G4 structures compared to those with longer linkers, **2a** and **2b**. Consistent binding was also observed across other G4-forming sequences, confirming the general ability of these ligands to interact with G4 topologies (Figure S1 and Table S3). In contrast, no binding was detected with non-G4-forming sequences such as complementary (ssDNA) and duplex (dsDNA) oligonucleotides, underscoring the specificity of these compounds for G4 structures. These results demonstrate that the conjugation of Pm at the fifth ring of the oxazoles in the 6OTD skeleton does not impair the G4-binding properties of L2H2-6OTD (Figure S2).^[36,38]^ Importantly, the ligands showed compatibility with both hybrid and parallel G4 topologies, which represent the majority of biologically relevant G4 structures reported to date.^[42]^ This versatility underscores the potential of G4L-PROTACs to target diverse G4 structures with high affinity.

### Binding of G4L-PROTACs to CRBN

**Figure 3.**
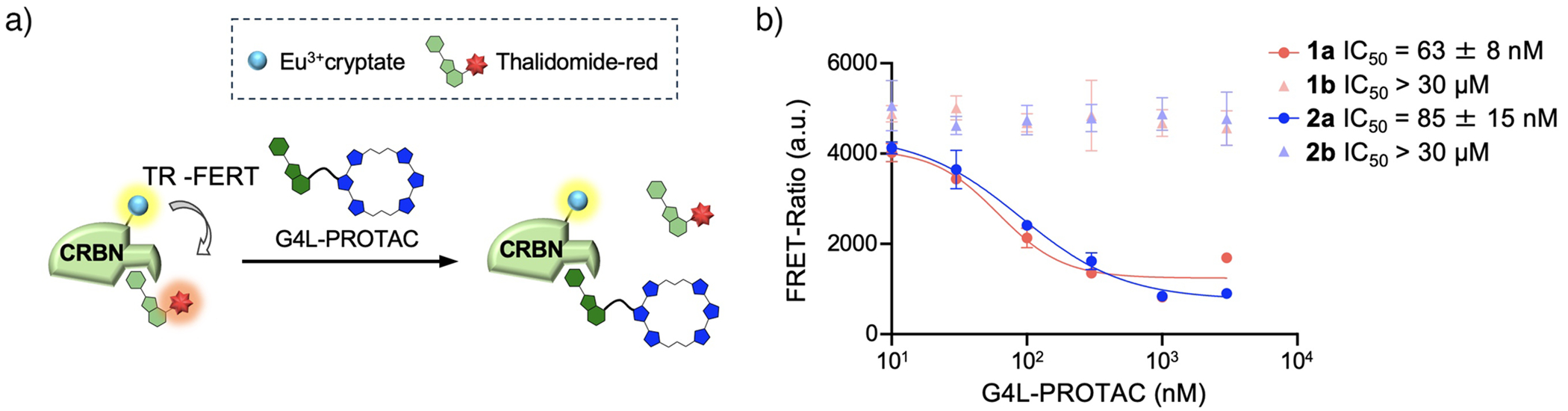
Functional evaluation of G4L-PROTACs utilizing cereblon. a) Schematic illustration of CRBN binding analysis using TR-FRET as an indicator. b) TR-FRET-based CRBN-binding assay. Data represents means ± SD (*n* = 3).

To assess whether conjugation of a G4 ligand to Pm affects its ability to bind CRBN, we employed a time-resolved fluorescence resonance energy transfer (TR-FRET) assay described in supporting information. The assay utilized Eu^3+^-cryptate-labeled CRBN and thalidomide-red as a fluorescent probe. Changes in TR-FRET signals were monitored upon the addition of compounds **1a**–**2b**, to evaluate their ability to displace thalidomide-red (Figure 3a). Compounds **1a** and **2a** caused a significant reduction in the TR-FRET signal, indicating displacement of thalidomide-red and binding of **1a** and **2a** to CRBN. The calculated IC_50_ values were 61 and 90 nM, respectively (Figure 3b). In contrast, compounds **1b** and **2b**, incorporating *N*-methylated Pm, produced no measurable changes in the TR-FRET signal, consistent with their lack of CRBN-binding activity. These results demonstrate that compounds **1a** and **2a** retain the ability to bind CRBN even after conjugation with the G4 ligand, while the *N*-methylated derivatives, **1b** and **2b**, serve as effective negative controls due to their inability to interact with CRBN. This confirms that the G4L-PROTACs **1a** and **2a** competitively bind CRBN without interference from the conjugated G4-binding (6OTD) domain.

### Probing Ternary Complex Formation with G4L-PROTACs

**Figure 4.**
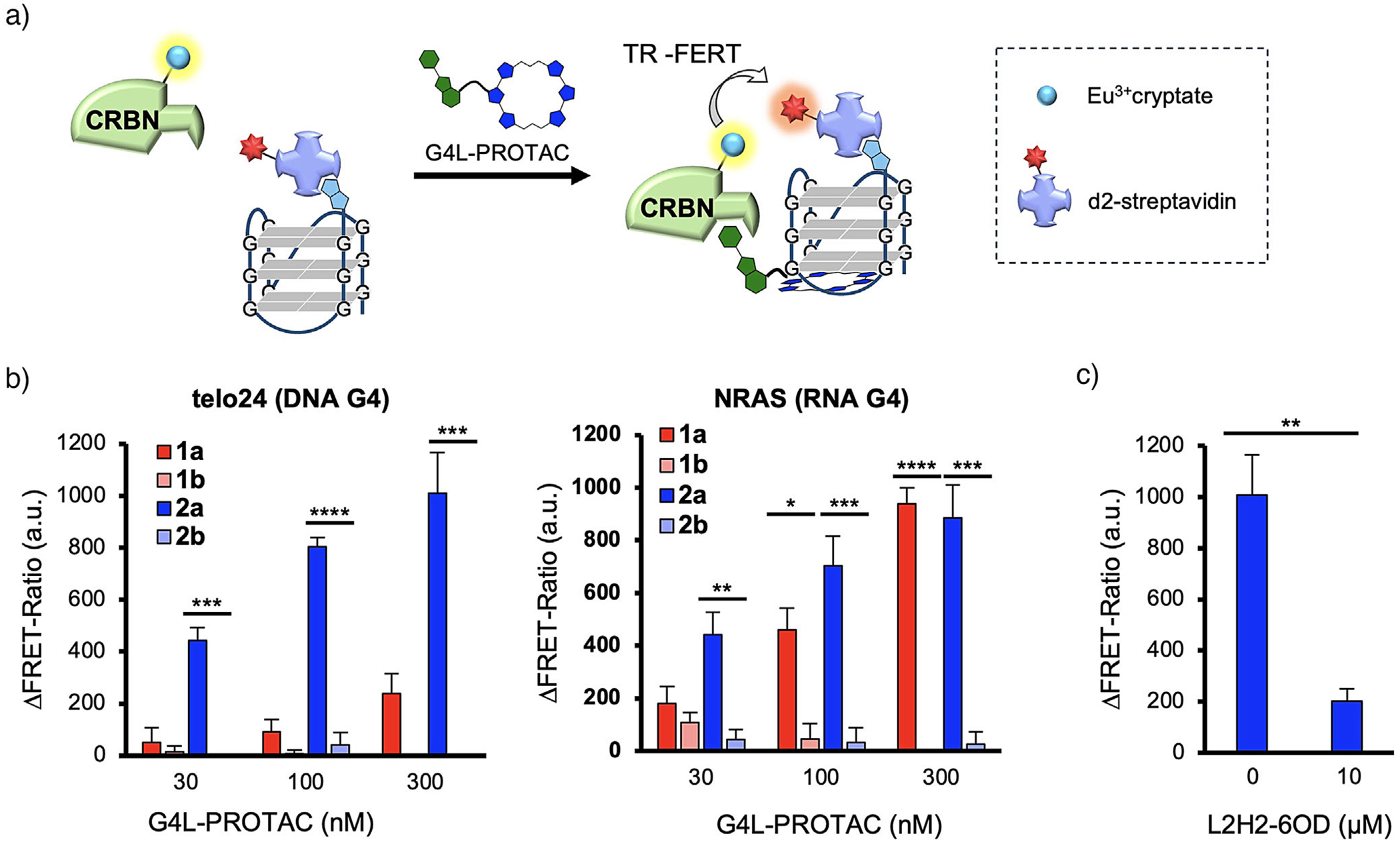
Formation of a CRBN-G4L-PROTACs-G4 ternary complex in vitro. a) Schematic illustration of the formation of a CRBN-G4L-PROTACs-G4 complex. b) Verification of ternary complex formation using G4 (telo24 and NRAS) with TR-FRET. c) Inhibition of ternary complex formation by excess L2H2-6OTD. Data represents means ± SD (*n* = 3). *p*-Values were determined using the unpaired two-tailed Student’s *t*-test. *p < 0.05; **p < 0.01; ***p < 0.001; ****p < 0.0001.

The mechanism of action of G4L-PROTACs involves the formation of a quaternary complex consisting of CRBN, the G4L-PROTACs, G4, and a G4-binding protein (G4BP). To investigate the formation of this quaternary complex in vitro, we developed a TR-FRET assay using Eu^3+^-cryptate-labeled CRBN and biotinylated G4 complexes tethered to streptavidin labeled with d2, which is a quencher for Eu^3+^ (Figure 4a). Experiments were conducted with telo24, a DNA hybrid-type G4, and NRAS, an RNA parallel-type G4, to evaluate the ability of compounds **1a**–**2b** to mediate quaternary complex formation (Figure 4b). In the case of telo24 G4, compound **2a**, which has a longer linker, produced significant TR-FRET signals at concentrations ranging from 30 nM to 300 nM, indicating efficient quaternary complex formation. In the case of higher concentration of **1a**–**2b** (>1 μM), the fluorescent signal from Pm disturbed the TR-FRET system. In contrast, **1a** with a shorter linker produced only weak TR-FRET signals under similar conditions. With NRAS G4, both **1a** and **2a** generated dose-dependent TR-FRET signals in the same concentration range, suggesting that the linker length plays a lesser role in quaternary complex formation with NRAS RNA G4 topology. In both cases, no TR-FRET signals were observed with **1b** or **2b**, which incorporate *N*-methylated Pm, consistent with their inability to bind CRBN.

To confirm the role of G4-6OTD interactions in the quaternary complex formation, an excess of L2H2-6OTD (10 μM, Figure 1c) was added to **2a** (300 nM). The addition of L2H2-6OTD disrupted the quaternary complex, resulting in a significant reduction in the TR-FRET signal (Figure 4c). Control experiments using biotin-labeled single-stranded or duplex oligonucleotides did not produce TR-FRET signals, confirming the specificity of quaternary complex formation (Figure S3). In general, PROTACs exhibit a characteristic dose-response phenomenon known as the “hook effect,” which occurs when the molecular glue can independently bind both the POI and CRBN at higher concentrations, thereby reducing ternary complex formation.^[43]^ To evaluate this, we tested **2a** using a TR-FRET assay at higher concentrations (30–3000 nM), which revealed a typical hook effect pattern (Figure S4).

These results demonstrate that both CRBN-Pm and G4-6OTD interactions are essential for quaternary complex formation. Furthermore, the linker length of G4L-PROTACs affected the efficiency of quaternary complex formation, depending on the topology of the target G4 structure, as demonstrated by the different behaviors observed with telo24 and NRAS G4s.

### G4L-PROTAC Reduces the Protein Level of DHX36

Building on the in vitro evidence suggesting that **2a** induces quaternary CRBN-G4–G4BP complex formation with high efficiency, we next focused on testing **2a** in cellular systems to evaluate its ability to degrade G4BPs. Since the TR-FRET results demonstrated that the longer linker in **2a** enhances quaternary complex formation both with DNA G4 (telo24) and with RNA G4 (NRAS), it was selected as a versatile candidate for further evaluation. Western blot analysis was used to quantify DHX36, a well-characterized G4BP, following treatment with **2a** and related controls. Due to concerns that the large molecular sizes of **2a** and **2b** might limit their cellular permeability, we evaluated cytotoxicity under conditions with or without a transfection reagent (Endo-Porter PEG).^[44]^ HeLa cells were treated with **2a** or **2b** (3–30 μM) in the presence or absence of a transfection reagent, and cell viability was assessed using Alamar-Blue assay. No acute toxicity was observed under any of the conditions examined. Based on these results, we adopted treatment conditions of 3–30 μM for 24 h to ensure sufficient exposure without inducing toxicity (Figure S5).

**Figure 5.**
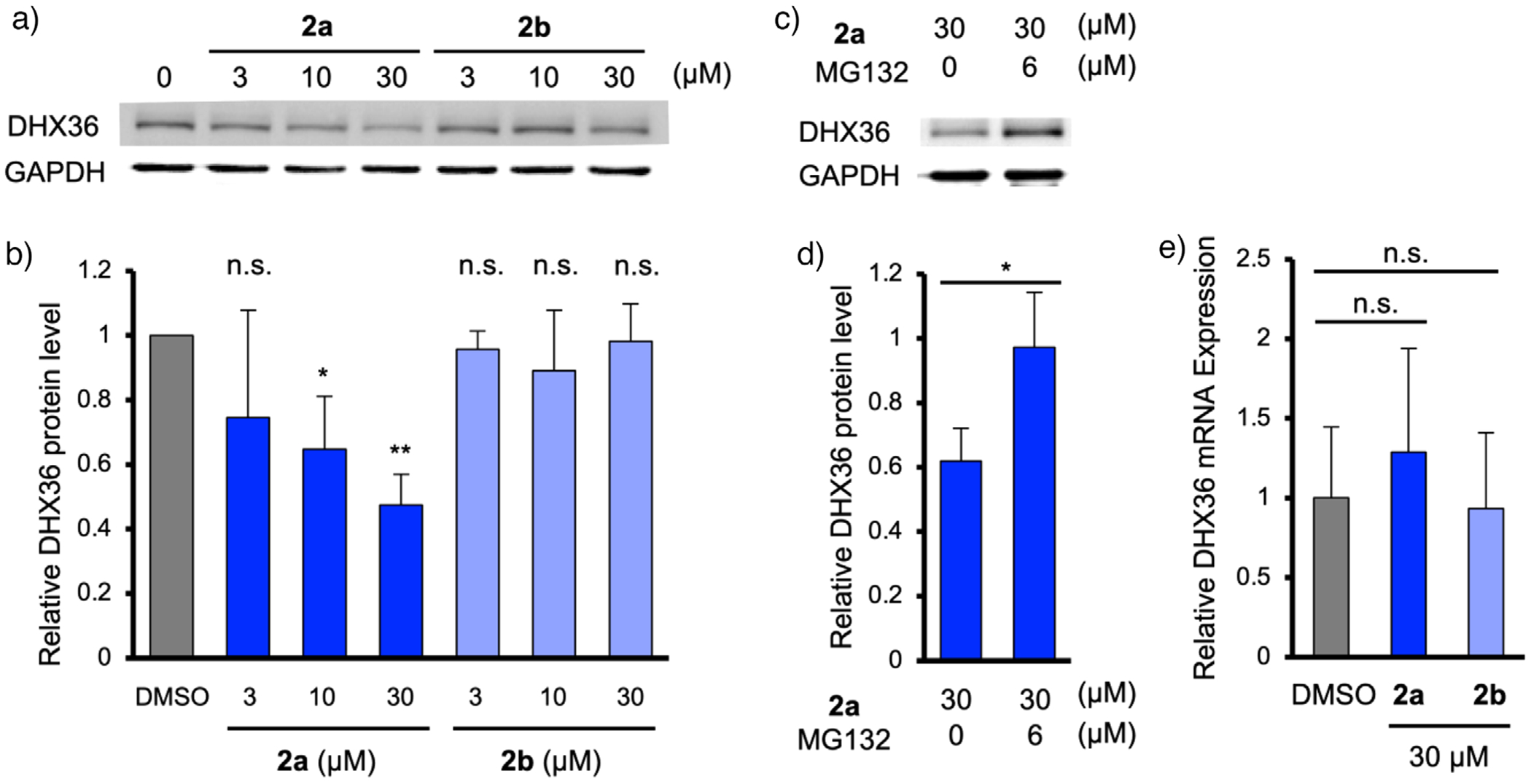
Application of G4L-PROTACs in HeLa cells. a) and b) Western blotting analysis of DHX36 degradation in HeLa cells treated with **2a** for 24 h. c) and d) Western blotting analysis of DHX36 degradation in HeLa cells treated with **2a** and protease inhibitor MG132 for 12 h. e) Quantification of DHX36 mRNA levels after cells were treated with **2a** or **2b** (30 μM) for 24 h by real-time PCR. Data represents means ± SD (*n* = 3). *p*-Values were determined using the unpaired two-tailed Student’s *t*-test. n.s., not significant; *p < 0.05; **p < 0.01.

We next treated HeLa cells with **2a** or **2b** (3–30 μM) for 24 h and quantified DHX36 protein levels. Without a transfection reagent, no degradation of DHX36 was observed for either compound (Figure S6). However, in the presence of the transfection reagent, treatment with **2a** resulted in a dose-dependent reduction in DHX36 protein, with 10 and 30 μM **2a** leading to 35% and 53% reductions, respectively (Figure 5a,b). In contrast, **2b**, which contains *N*-methylated Pm, had no effect on DHX36 level. Furthermore, co-treatment with the proteasome inhibitor MG132 abolished the reduction in DHX36 protein levels observed with **2a**, confirming that the degradation was proteasome-dependent (Figure 5c,d). To ensure that the reduction in DHX36 protein levels was not caused by transcriptional inhibition, we performed RT-PCR to quantify DHX36 mRNA. No changes in mRNA levels were detected following treatment with 30 μM **2a** or **2b** (Figure 5e), indicating that the observed reduction in DHX36 protein was due to proteasomal degradation mediated by the interaction between **2a**, CRBN, and intracellular G4s. Although ternary complex formation was observed in vitro, DHX36 degradation in cells may occur through competition or vicinity binding at clustered G4s rather than requiring a strict quaternary complex. These results demonstrate that G4L-PROTAC **2a** selectively induces the proteasomal degradation of DHX36, highlighting its potential as a tool for targeting G4BPs in cellular systems.

We further confirmed that **2a** also degrades nucleolin (NCL), a well-characterized G4-binding protein known to bind and stabilize the c-myc promoter G4.^[45]^ At 10 μM, NCL was reduced, while the effect diminished at 30 μM, consistent with a PROTAC hook effect (Figure S7A). In contrast, endothelial differentiation-related factor 1 (EDF1), a nucleic acid-binding protein,^[46]^ has not been reported to bind G4 structures, and accordingly, was not degraded by **2a**, supporting a G4-dependent mechanism (Figure S7B).

### G4L-PROTAC 2a Promotes G4 Accumulation in Cells in Association with DHX36 Degradation

**Figure 6.**
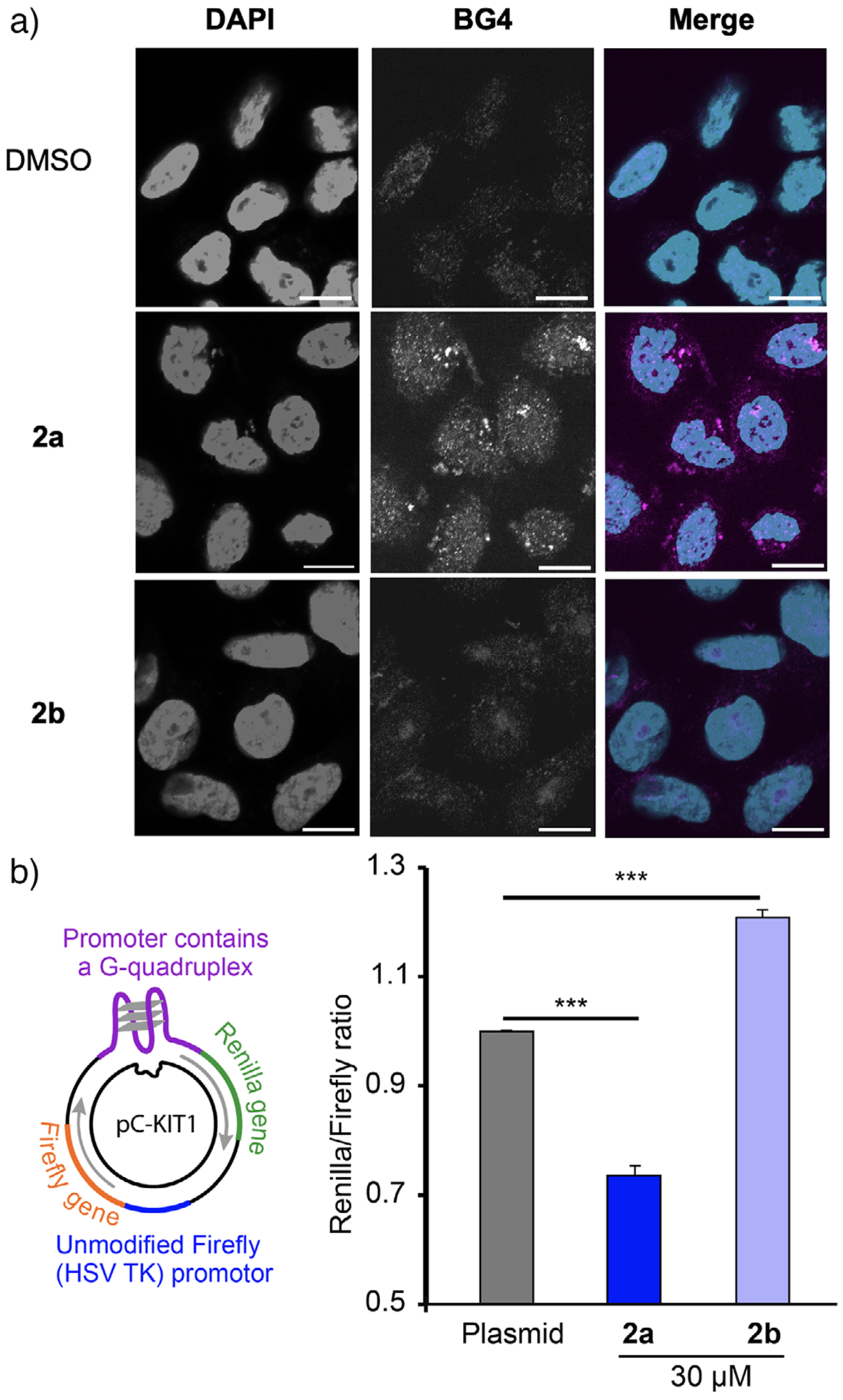
a) Increased G4 signal in HeLa cells following treatment with **2a**. HeLa cells were untreated (control) or treated with **2a** or **2b** (30 μM) for 24 h and subjected to immunofluorescence staining. G4 structures were visualized by immunostaining with BG4, shown in magenta in the images. Nuclei were counterstained with DAPI, shown in cyan. Scale bar: 10 μm. b) HEK 293 cell-based G4-luciferase reporter assay. HEK 293 cells were treated with **2a** or **2b** (30 μM) for 24 h. Data are shown as mean ± SD. *p*-Values were determined by *t*-test. ***p < 0.001.

In HeLa cells, treatment with **2a** led to proteasomal degradation of DHX36, a major G4 helicase, as confirmed by Western blotting. To evaluate the functional consequence of this degradation, we performed BG4-based immunofluorescence staining after 24-h treatment with either **2a** or the control compound **2b** (Figure 6a). Cells treated with **2a** exhibited a pronounced increase in cytoplasmic G4 signal compared to those treated with **2b**, indicating that loss of DHX36 impaired the resolution of G4 structures. Given that the BG4 signal is predominantly localized to the cytoplasm, these G4 structures are likely of RNA origin, which is consistent with the fact that DHX36 can unwind RNA G4s.^[47]^ These observations demonstrate that G4L-PROTACs can modulate the dynamic equilibrium between G4 structures and their binding proteins in live cells via selective protein degradation. Importantly, such phenotypic readouts cannot be achieved using conventional G4-decoy-based PROTAC systems. This is because the synthetic G4 DNA motifs used as warheads in those systems directly compete with BG4 antibodies for G4 recognition, thereby interfering with immunodetection. In contrast, our small-molecule G4L-PROTAC design does not interfere with BG4 binding, thus uniquely enabling both functional manipulation of G4-binding proteins and reliable phenotypic observation in the same system. While DHX36 degradation is a major contributor to the observed accumulation of G4s, additional G4-associated proteins may also participate in this process, since **2a** is capable of binding to G4 structures that are recognized by not only DHX36 but also multiple G4BPs.

To directly assess gene expression outcomes, we used a cell-based luciferase reporter in which a G4 sequence was placed upstream of the coding region.^[48,49]^ Treatment with **2a** led to a decrease in luciferase activity, whereas **2b** unexpectedly increased reporter output (Figure 6b). We interpret these results as the superposition of two mechanistic layers: i) a ubiquitination-dependent effect—recruitment of CRBN by **2a** promotes proteasomal degradation of G4-associated factors (e.g., DHX36), thereby impairing G4 unwinding and reducing Renilla gene expression; and ii) a ligand-intrinsic effect—when CRBN recruitment is disabled in **2b**, the G4 ligand moiety alone can modulate G4–protein interactions that support transcription and/or translation. Consistent with our recent finding that G4 ligands can either competitively inhibit or support G4–G4BP interactions depending on sequence and structural context, the latter scenario likely reflects a supportive/vicinity-G4 mode that facilitates productive protein engagement or G4 remodeling to a transcription/translation-permissive state.^[50,51]^

### G4L-PROTAC Targets a Broad Range of G4-Associated Proteins

Following the demonstration of **2a**-mediated DHX36 degradation and its binding to various G4 structures, we anticipated that **2a** would also induce the proteasomal degradation of other G4BPs in cells. To investigate this, we performed proteomic analyses of cells treated with **2a** for 24 h and compared protein levels to those in cells treated with **2b**. Compound **2b** retains G4-binding capacity but lacks CRBN-binding activity, serving as a control to distinguish degradation from G4-ligand-induced transcriptional or translational suppression. Proteomic profiling revealed that 682 proteins were downregulated by >10% upon treatment with **2a** compared to **2b** (Figure S8A and Table S4). Gene ontology analysis of these proteins indicated significant enrichment in RNA-related processes, particularly metabolism of RNA and mRNA metabolic processes, highlighting the involvement of G4 structures in RNA regulation (Figure S8B). Comparison of the proteins reduced by **2a** with the 201 G4-related proteins (G4RPs) identified by G4-LIMCAP using a photocrosslinking G4 ligand^[ 28]^ revealed an overlap of 57 proteins (20%; Figure S8C). This overlap validates the capability of our method to selectively target G4RPs. Although the canonical RGG motif showed no significant enrichment, likely due to its limited occurrence, the proteins degraded by **2a** exhibited a higher density of aromatic–glycine (*π*–Gly) motifs such as YGG, FGG, and WGG,^[52,53]^ previously reported to contribute to G4 binding compared to unchanged proteins (Figure S8D).

The larger number of proteins degraded by **2a** compared to the proteins identified by G4-LIMCAP could be attributed to several factors. First, the spatial reach of ubiquitin transfer by E2 enzymes is greater than that of photocrosslinking, which may not give quantitative adducts and is restricted to the short duration of light exposure, thereby enabling ubiquitination of nearby but not directly contacted G4BPs.^[54]^ Second, the observed protein reductions may include secondary effects resulting from the proteasomal degradation of G4BPs, which could influence downstream cellular processes.^[47]^ Third, the binding mode of the G4 ligand 6OTD may also help explain why more proteins are degraded by **2a**. 6OTD and G4BPs can bind opposite ends of the same G4 or to adjacent G4s, allowing G4L-PROTACs to recruit not only direct G4 binders but also proteins located near clustered G4 sites, thereby broadening the spectrum of proteins subjected to ubiquitination.^[50]^ Fourth, the resulting proteomic analysis may include both false positives and false negatives. False positives could arise from ubiquitination of proteins located near G4s bound by **2a**, whereas false negatives may result from low protein abundance, mass spectrometry limitations, or the applied cutoff threshold. Despite these limitations, the G4L-PROTAC approach provides a unique advantage: an extended time window for ubiquitination. In prior studies using photocrosslinking, the identification of G4RPs was limited to the duration of UV light exposure, typically a few minutes,^[ 25,28]^ due to the cytotoxic effects of UV irradiation. In contrast, G4L-PROTACs remain active throughout the treatment period, enabling continuous ubiquitination of G4-bound proteins over 24 h. This extended activity increases the likelihood of capturing transient or low-abundance interactions, thereby enhancing the scope of G4RP identification.

G4L-PROTACs operate by forming a quaternary complex with CRBN and G4, facilitating the recruitment of ubiquitin transfer machinery to G4-bound proteins. This mode of action not only enables the selective degradation of G4-bound G4BPs but also allows the transfer of ubiquitin to these proteins, effectively tagging them for proximity labeling.^[23,24]^ Ubiquitinated G4RPs can be enriched using anti-ubiquitin antibodies and subsequently identified, providing a powerful approach for studying G4RPs and their interactions in cellular contexts.^[55,56]^

## Conclusion

In this study, we developed G4L-PROTACs that selectively induce the proteasomal degradation of G4BPs through ternary complex formation with CRBN and G4 structures. Cellular and proteomic analyses demonstrated that G4L-PROTACs not only degrade representative G4BPs but also expand the coverage of G4-related proteins compared to photocrosslinking approaches, owing to their extended time window for ubiquitination. These findings establish G4L-PROTACs as versatile tools to probe the dynamic interplay between G4 structures and their related proteins. Beyond functional studies, this approach provides a foundation for proximity labeling strategies and offers unique advantages over conventional genetic perturbation methods, highlighting its potential for therapeutic applications in G4-driven diseases.

## Supplementary Material

Supplementary file

Additional supporting information can be found online in the Supporting Information section

## Figures and Tables

**Table 1:** *K*_d_ values (nM) of **1a**–**2b** for G4 sequences.

Oligonucleotides	*K*_d_ (nM)			
	1a	1b	2a	2b
telo24	108 ± 75	33 ± 17	356 ± 210	155 ± 29
NRAS	65 ± 2	10 ± 5	68 ± 56	96 ± 28
ssDNA	>1200	>1200	>1200	>1200
dsDNA	>1200	>1200	>1200	>1200

## Data Availability

The data that support the findings of this study are available in the Supporting Information of this article.
