# Supplementary material for: Degradation of G-Quadruplex-Binding Proteins by G4L-PROTAC via Quaternary Complex Formation
Source: Angew Chem Int Ed Engl. Author manuscript; Available in PMC 2026 Mar 4. (PMC12959348; doi:10.1002/anie.202515045)
Supplement: Supplementary file [file NIHMS2144399-supplement-Supplementary_file.pdf]

Supporting information

Degradation of G-Quadruplex Binding Proteins by G4L-PROTACs via Quaternary Complex Formation

Rena Nohara,[a] Yuma Tanaya, [a] Mohammad Jafar Sheikhi, [a] Pratiksha Chaudhary,[b] Grinsun Sharma, [b] Hanbin Mao, [b] Kazuo Nagasawa, [a] Masayuki Tera*[a]

[a] Department of Biotechnology and Life Science, Tokyo University of Agriculture and Technology, 2-24-16 Naka-cho, Koganei, Tokyo 184-8588, Japan

E-mail: tera@go.tuat.ac.jp

[b] Department of Chemistry and Biochemistry, School of Biomedical Sciences, Advanced Materials and Liquid Crystal Institute, Kent State University, Kent, Ohio 44242, USA

Table of Contents:

Experimental Methods	S3
General synthetic methods and reagents	S3
General experimental procedures for biochemistry experiments	S10
Table S1	S16
Table S2	S16
Figure S1	S17
Table S3	S17
FigureS2	S18
Figure S3	S19
Figure S4	S19
Figure S5	S20
Figure S6	S20
Figure S7	S20
Figure S8	S21
Whole gel images	S22
^{1}H and ^{13}C NMR spectra	S23
Table S5	S39
References for supporting information	S61

Experimental Methods

General synthetic methods and reagents. **S1**, **S3–S6** and L2H2-6OTD were synthesized as previously reported[1]. All other reagents were obtained in the molecular biology grades from Sigma-Aldrich, Wako chemicals, or TCI. Flash chromatography was performed on Silica gel 60 (spherical, particle size 40 ~ 100 mm; Kanto Co., Inc., Japan). ^{1}H and ^{13}C NMR spectra were recorded on JEOL JNM-ECX 400 (400 MHz) and JEOL JNM-ECX 500 (500 MHz). The spectra are referenced internally according to residual solvent signals of DMSO-d_6 (^{1}H NMR; δ = 2.50 ppm). Data for 1H NMR are recorded as follows: chemical shift (δ, ppm). Mass spectra were recorded on JEOL JMS-T100LC spectrometer.

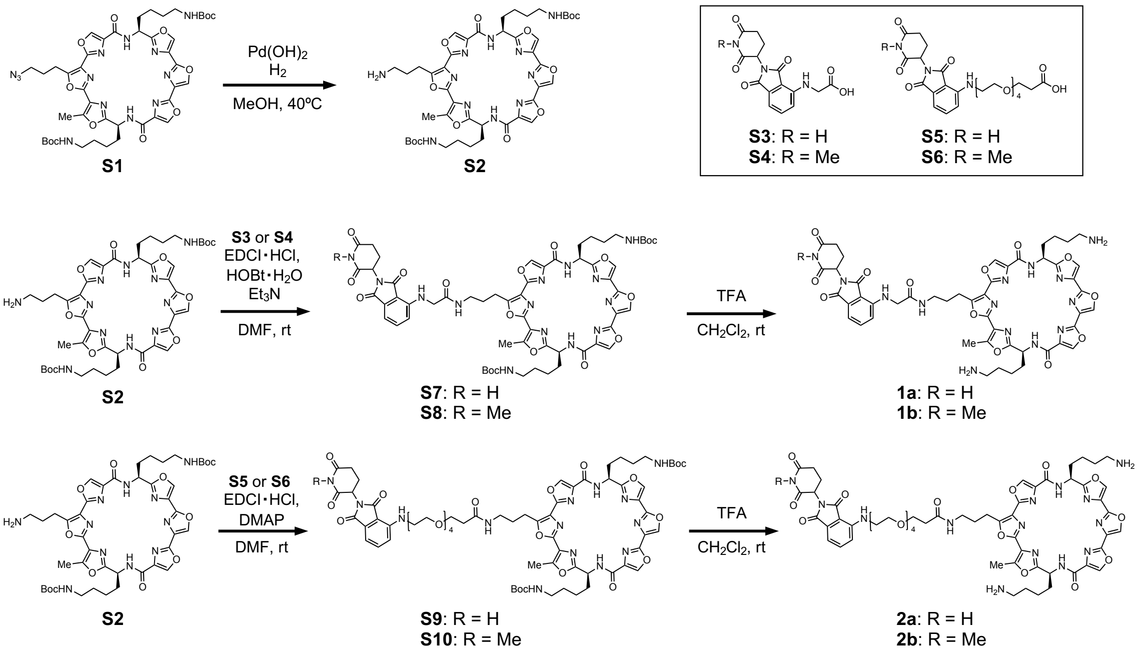

Scheme S1. Synthesis of **1a**–**2b**.

Synthesis of **S2**

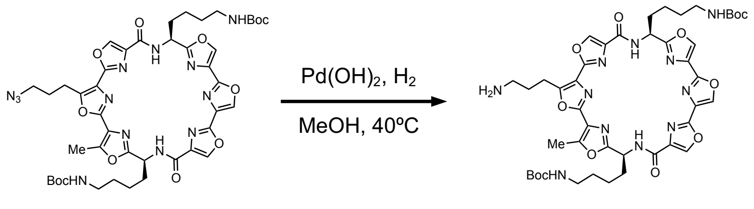

S1 **S2**

To a solution of the **S1** (14.6 mg, 0.015 mmol) in MeOH-THF (1:1 2 mL) was added $Pd(OH)_2$ (3

mg, 20% wt.) at room temperature. After stirring for 2 h at 40 °C under H_2 atmosphere, the reaction mixture was filtered through a pad of celite. The filtrates were concentrated in vacuo. The residue was purified by amino silica gel column chromatography (chloroform/methanol = 9:1, NH_3 0 M to 0.7 M) to give **S2** as a white solid (14 mg, 0.015 mmol, 98% yield).

^{1}H-NMR (400 MHz, DMSO-*d$_6$*) δ 9.12 (s, 1H), 9.10 (s, 1H), 8.91 (s, 1H), 8.87 (s, 1H), 8.32 (s, 2H), 6.77 (d, *J* = 5.5 Hz, 2H), 5.43 (s, 1H), 5.34 (s, 1H), 3.19 (s, 2H), 2.85-2.79 (m, 6H), 2.71 (s, 3H), 2.04-1.89 (m, 6H), 1.33-1.08 (m, 33H); ^{13}C-NMR (100 MHz, DMSO-*d$_6$*) δ 164.6, 162.1, 158.8, 158.7, 155.7, 155.5, 155.4, 154.5, 153.8, 151.5, 142.5, 142.3, 141.7, 141.0, 136.0, 135.9, 129.8, 128.4, 124.6, 123.7, 79.2, 77.2, 47.3, 47.2, 33.3, 29.2, 28.2, 22.7, 21.1, 21.0, 11.6; HRMS (ESI-TOF) 930.4119 (M + H)$^+$ calcd for $C_{44}H_{56}N_{11}O_{12}$ 930.4104.

Synthesis of **S7**

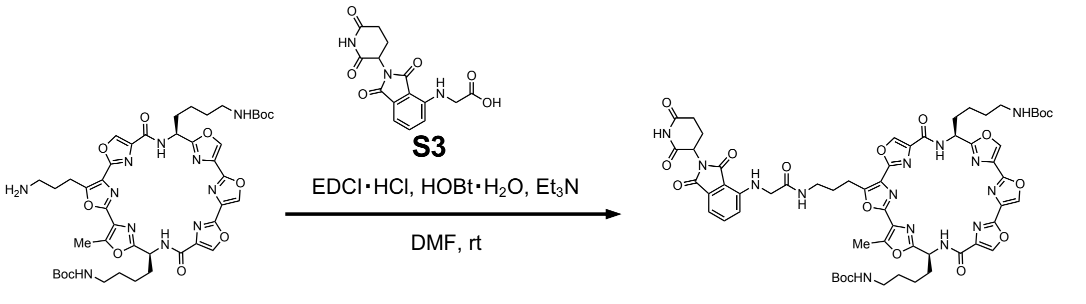

S2 **S7**

To a solution of **S2** (12.4 mg, 0.013 mmol) in DMF (1 mL) was added EDCI•HCl (7.6 mg, 0.040 mmol) , HOBt•H_2O (5.4 mg, 0.040 mmol) and Et_3N (9.2 µL, 0.067 mmol) at room temperature under Ar atmosphere. After stirring 30 min, the reaction was added **S3** (5.3 mg, 0.016 mmol). After stirring overnight, the reaction was concentrated in vacuo with toluene for three times. The residue was purified by column chromatography on silica gel (chloroform/methanol = 60:1 to 50:1) to **S7** as a yellow solid (7.0 mg, 0.0056 mmol, 42% yield).

^{1}H-NMR (400 MHz, DMSO-*d$_6$*) δ 11.11 (s, 1H), 9.10 (d, *J* = 11.0 Hz, 2H), 8.89 (d, *J* = 14.7 Hz, 2H), 8.32 (d, *J* = 6.4 Hz, 3H), 8.27-8.20 (m, 1H), 7.54 (t, *J* = 7.8 Hz, 1H), 7.08-7.03 (m, 1H), 6.95 (s, 1H), 6.87-6.76 (m, 4H), 5.43-5.40 (m, 1H), 5.29 (d, *J* = 5.5 Hz, 1H), 5.07 (dd, *J* = 12.8, 5.5 Hz, 1H), 3.90 (d, *J* = 5.5 Hz, 2H), 3.50 (s, 1H), 3.27-3.13 (m, 3H), 2.93-2.82 (m, 8H), 2.69 (s, 2H), 2.65-2.56 (m, 1H), 2.04-1.91 (m, 9H), 1.43-1.23 (m, 42H); ^{13}C-NMR (100 MHz, DMSO-*d$_6$*) δ 172.8, 170.1, 168.7, 168.5, 167.3, 164.5, 162.0, 158.8, 158.6, 155.7, 155.5, 154.5, 153.9, 151.5, 145.7, 142.5, 142.3, 141.7, 141.0, 136.1, 136.0, 135.9, 132.0, 129.8, 128.4, 124.6, 123.7, 117.4, 111.0, 109.8, 79.2, 77.2, 69.9, 69.8, 69.2, 48.6, 47.2, 45.2, 38.1, 33.4, 31.0, 29.1, 28.2, 28.1, 27.0, 23.0, 22.2, 21.1, 20.9, 11.5; HRMS (ESI-TOF) 1265.4628 (M + Na)$^+$ calcd for $C_{59}H_{66}N_{14}NaO_{17}$ 1265.4623.

Synthesis of **1a**

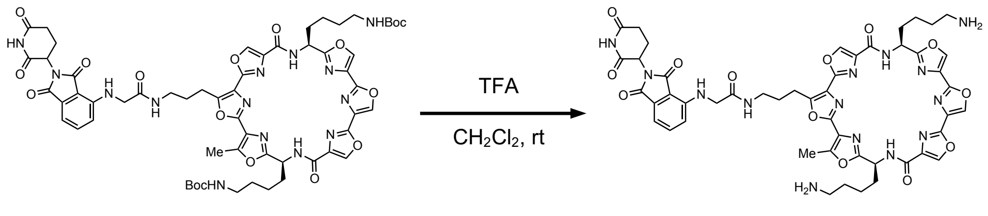

S7 **1a**

To a solution of **S7** (13 mg, 0.010 mmol) in CH_2Cl_2 (1 mL) was added TFA (0.5 mL) at room temperature. After stirring for 30 min, the reaction was concentrated in vacuo with toluene for three times. The residue was purified by waters sep-paq (H_2O/methanol = 1:0 to 2:3) to give **1a** as a yellow solid (10 mg, 0.0079 mmol, 79% yield).

^{1}H-NMR (400 MHz, DMSO-*d*$_6$) δ 9.13 (d, J = 5.5 Hz, 1H), 8.93 (s, 0H), 8.89 (s, 0H), 8.31-8.24 (m, 2H), 7.57-7.52 (m, 1H), 7.04 (d, J = 6.9 Hz, 1H), 6.94 (s, 1H), 6.84 (d, J = 8.7 Hz, 1H), 5.44 (d, J = 6.9 Hz, 1H), 5.30 (d, J = 7.3 Hz, 0H), 5.07 (dd, J = 13.3, 5.5 Hz, 1H), 3.91 (s, 2H), 2.89-2.85 (m, 1H), 2.75-2.69 (m, 6H), 2.60 (d, J = 17.9 Hz, 2H), 2.04-1.90 (m, 6H), 1.62-1.16 (m, 9H); ^{13}C-NMR (100 MHz, DMSO-*d*$_6$) δ 173.1, 170.3, 168.9, 168.7, 167.5, 164.6, 162.2, 159.1, 158.2, 157.9, 155.9, 155.7, 154.7, 154.2, 151.8, 145.9, 142.8, 142.6, 141.3, 136.4, 136.2, 136.0, 132.2, 129.9, 128.7, 124.8, 123.9, 117.6, 111.2, 110.0, 70.0, 48.8, 47.0, 45.3, 40.3, 33.6, 31.2, 27.2, 27.0, 22.4, 21.1, 20.9, 11.8; HRMS (ESI-TOF) 1043.3769 $(M + H)^+$ calcd for $C_{49}H_{51}N_{14}O_{13}$ 1043.3755.

Synthesis of **S8**

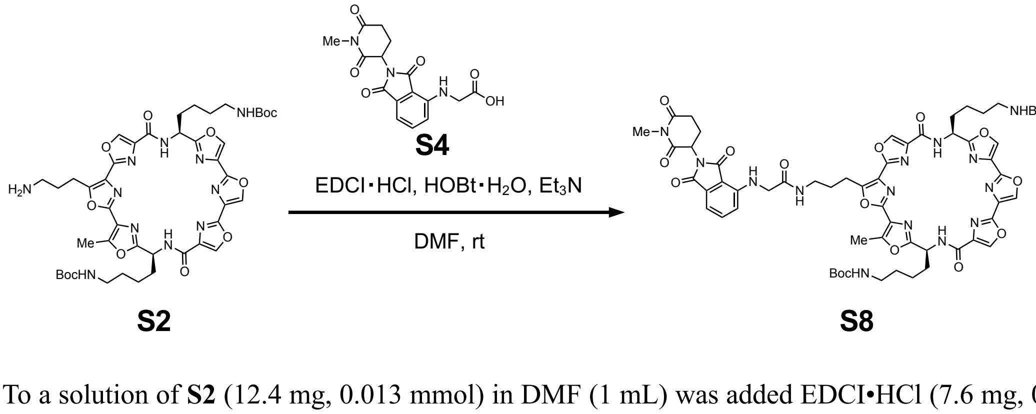

S2 **S8**

To a solution of **S2** (12.4 mg, 0.013 mmol) in DMF (1 mL) was added EDCI•HCl (7.6 mg, 0.040 mmol) , HOBt•H_2O (7.6 mg, 0.040 mmol) and Et_3N (9.2 μL, 0.067 mmol) at room temperature under Ar atmosphere. After stirring 30 min, the reaction was added **S4** (5.5 mg, 0.016 mmol). After stirring

overnight, the reaction was concentrated in vacuo with toluene for three times. The residue was purified by column chromatography on silica gel (chloroform/methanol = 100:1 to 80:1) to **S8** as a yellow solid (15 mg, 0.012 mmol, 92% yield).

^{1}H-NMR (400 MHz, DMSO-d_6) δ 9.11 (s, 1H), 9.08 (s, 1H), 8.90 (s, 1H), 8.86 (s, 1H), 8.32 (d, J = 8.2 Hz, 2H), 8.27-8.20 (m, 2H), 7.54 (t, J = 7.8 Hz, 1H), 7.04 (d, J = 7.3 Hz, 1H), 6.94 (s, 1H), 6.84 (d, J = 8.7 Hz, 1H), 6.76 (s, 2H), 5.42 (dd, J = 12.1, 5.3 Hz, 1H), 5.28 (d, J = 4.1 Hz, 1H), 5.13 (dd, J = 13.1, 5.3 Hz, 1H), 3.90 (s, 2H), 3.27-3.13 (m, 4H), 3.01 (s, 3H), 2.95-2.91 (m, 0H), 2.84-2.74 (m, 4H), 2.69 (s, 3H), 2.55 (dd, J = 13.3, 4.1 Hz, 1H), 2.05-1.90 (m, 7H), 1.33-1.07 (m, 30H); ^{13}C-NMR (100 MHz, DMSO-d_6) δv 171.8, 169.8, 168.7, 168.5, 167.3, 164.5, 162.0, 158.9, 158.7, 155.7, 155.5, 154.5, 153.9, 151.5, 145.8, 142.5, 142.3, 141.7, 141.0, 136.2, 136.1, 135.9, 132.0, 129.8, 128.5, 124.6, 123.7, 117.5, 111.0, 109.8, 79.2, 77.3, 49.1, 47.4, 47.2, 45.2, 38.1, 33.4, 33.3, 31.1, 29.1, 28.2, 27.0, 26.6, 23.0, 21.4, 21.1, 20.9, 11.5; HRMS (ESI-TOF) 1279.4786 (M + Na)$^+$ calcd for $C_{60}H_{68}N_{14}NaO_{17}$ 1279.4779.

Synthesis of **1b**

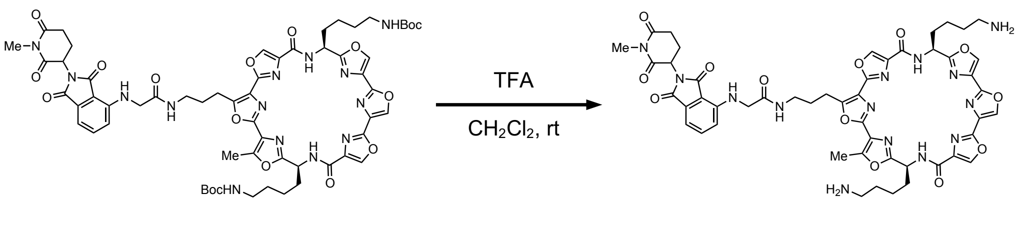

S8 **1b**

To a solution of **S8** (20 mg, 0.0159 mmol) in CH_2Cl_2 (1 mL) was added TFA (0.5 mL) at room temperature. After stirring for 30 min, the reaction was concentrated in vacuo with toluene for three times. The residue was purified by waters sep-paq (H_2O/methanol = 1:0 to 2:3) to give **1b** as a yellow solid (19.7 mg, 0.0153 mmol, 96% yield).

^{1}H-NMR (400 MHz, DMSO-d_6) δ 8.94 (d, J = 6.0 Hz, 4H), 8.73 (s, 2H), 8.68 (d, J = 8.2 Hz, 2H), 8.09 (dd, J = 18.3, 7.8 Hz, 6H), 7.55 (s, 13H), 7.35 (t, J = 7.8 Hz, 2H), 6.85 (d, J = 6.9 Hz, 2H), 6.75 (s, 2H), 6.65 (d, J = 8.2 Hz, 2H), 5.25 (dd, J = 12.6, 5.3 Hz, 2H), 5.11 (q, J = 3.5 Hz, 2H), 4.94 (dd, J = 13.1, 5.3 Hz, 2H), 3.72-3.67 (m, 3H), 3.09-2.95 (m, 7H), 2.81 (d, J = 11.0 Hz, 7H), 2.75 (dd, J = 17.2, 5.3 Hz, 2H), 2.59-2.53 (m, 11H), 2.41-2.33 (m, 3H), 2.31 (t, J = 1.8 Hz, 5H), 1.88-1.85 (m, 6H), 1.77-1.71 (m, 5H), 1.35-1.22 (m, 12H), 1.03-0.97 (m, 6H); ^{13}C-NMR (100 MHz, DMSO-d_6) δ 171.8, 169.8, 168.7, 168.5, 167.3, 164.4, 162.0, 158.9, 158.7, 155.7, 155.5, 154.5, 154.0, 151.6, 145.8, 142.6, 142.4, 141.8, 141.1, 136.2, 136.0, 135.8, 132.0, 129.7, 128.5, 124.6, 123.7, 117.5, 111.0, 109.8, 49.1, 47.2, 47.1, 45.1, 40.4, 38.6, 38.1, 33.4, 31.1, 27.0, 26.7, 26.6, 22.9, 21.4, 20.9, 20.8, 11.5; HRMS (ESI-TOF) 1057.3928 (M + H)$^+$ calcd for $C_{50}H_{53}N_{14}O_{13}$ 1057.3911.

Synthesis of **S9**

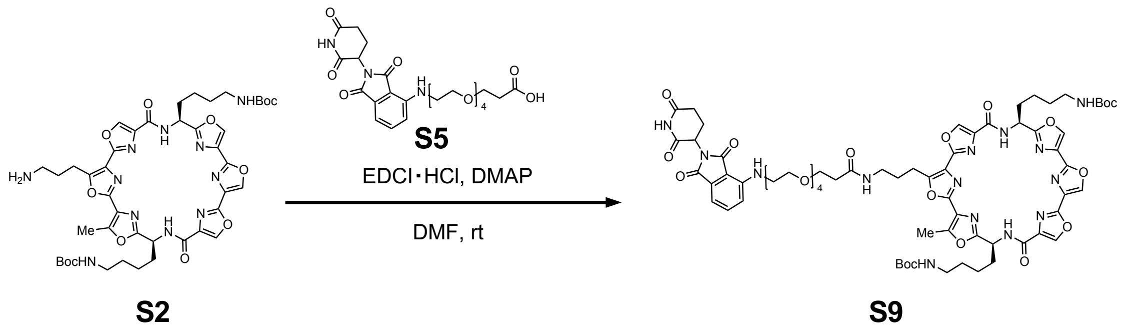

S2 **S9**

To a solution of **S2** (14.6 mg, 0.016 mmol) in CH_2Cl_2-THF (3 mL) was added EDCI•HCl (9.0 mg, 0.047 mmol) and DMAP (5.8 mg, 0.047 mmol) at room temperature under Ar atmosphere. After stirring 30 min, the reaction was added **S5** (5.5 mg, 0.016 mmol). After stirring overnight, the reaction was concentrated in vacuo with toluene for three times. The residue was purified by column chromatography on silica gel (chloroform/methanol = 80:1 to 60:1) and preparative TLC (chloroform/ethyl acetate/methanol 3:2:1) to give **S9** as a yellow solid (7.8 mg, 0.0054 mmol, 35% yield).

^{1}H-NMR (500 MHz, DMSO-d_6) δ 11.07 (d, J = 6.9 Hz, 1H), 9.11 (s, 1H), 9.08 (d, J = 2.3 Hz, 1H), 8.90 (d, J = 1.1 Hz, 1H), 8.85 (s, 2H), 8.30 (dd, J = 19.8, 7.2 Hz, 2H), 7.94 (t, J = 5.4 Hz, 2H), 7.55 (t, J = 8.0 Hz, 2H), 7.15-7.09 (m, 2H), 7.00 (d, J = 6.9 Hz, 1H), 6.77 (t, J = 5.4 Hz, 3H), 6.55 (s, 1H), 5.41 (q, J = 5.9 Hz, 2H), 5.34-5.30 (m, 1H), 5.06-4.99 (m, 2H), 3.61-3.44 (m, 40H), 3.19-3.10 (m, 6H), 2.83 (s, 7H), 2.70 (d, J = 2.9 Hz, 4H), 2.29 (t, J = 6.3 Hz, 3H), 2.05 (t, J = 5.2 Hz, 5H), 1.89 (t, J = 6.9 Hz, 6H), 1.40-1.22 (m, 51H); ^{13}C-NMR (125 MHz, DMSO-d_6) δ 172.8, 170.1, 168.9, 167.2, 164.5, 162.0, 158.8, 158.7, 155.6, 155.5, 154.5, 153.9, 151.5, 146.4, 142.4, 142.2, 141.7, 141.0, 136.2, 136.0, 135.9, 132.0, 129.8, 128.5, 124.6, 123.7, 117.4, 110.6, 109.2, 77.2, 69.8, 69.7, 69.7, 69.5, 68.9, 66.8, 48.6, 41.7, 39.8, 37.8, 36.2, 33.3, 31.0, 29.1, 28.1, 27.2, 22.9, 22.1, 21.1, 20.9, 11.5; HRMS (ESI-TOF) 1433.6027 $(M + H)^+$ calcd for $C_{68}H_{85}N_{14}O_{21}$ 1433.6008.

Synthesis of **2a**

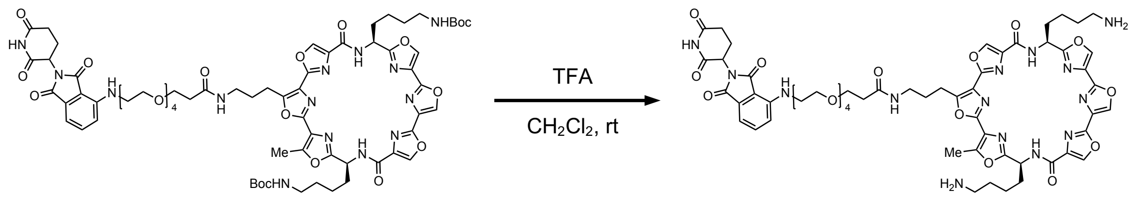

S9 **2a**

To a solution of **S9** (7.8 mg, 0.0054 mmol) in CH_2Cl_2 (1 mL) was added TFA (0.5 mL) at room temperature. After stirring for 30 min, the reaction was concentrated in vacuo with toluene for three times to give **2a** as a yellow solid (7.1 mg, 0.0049 mmol, 90% yield).

^{1}H-NMR (400 MHz, DMSO-d_6) δ 9.13-9.11 (m, 2H), 8.92 (d, J = 1.4 Hz, 1H), 8.87 (d, J = 1.4 Hz, 1H), 8.31-8.26 (m, 2H), 7.96 (t, J = 4.8 Hz, 1H), 7.55 (t, J = 7.8 Hz, 2H), 7.11 (t, J = 8.2 Hz, 1H), 7.00 (d, J = 6.9 Hz, 1H), 6.55 (s, 1H), 5.43 (d, J = 6.9 Hz, 1H), 5.34 (d, J = 6.4 Hz, 1H), 5.04-4.98 (m, 1H), 3.60-3.37 (m, 79H), 3.16 (d, J = 5.5 Hz, 5H), 2.91-2.83 (m, 2H), 2.73-2.70 (m, 8H), 2.61 (d, J = 32.5 Hz, 1H), 2.29 (t, J = 6.4 Hz, 3H), 2.06 (s, 3H), 1.90 (d, J = 6.9 Hz, 5H), 1.51-1.17 (m, 4H); ^{13}C-NMR (100 MHz, DMSO-d_6) δ 172.9, 170.1, 168.9, 167.2, 164.4, 162.0, 158.9, 158.7, 158.1, 155.7, 155.5, 154.5, 154.0, 151.6, 146.3, 142.5, 142.3, 141.8, 141.1, 136.2, 136.0, 135.8, 132.0, 129.7, 128.5, 124.6, 123.7, 117.4, 110.6, 109.2, 69.7, 69.7, 69.5, 68.9, 66.8, 48.6, 47.2, 47.1, 41.7, 38.7, 37.8, 36.1, 33.4, 31.0, 27.2, 27.0, 22.8, 22.1, 20.9, 20.9, 11.5; HRMS (ESI-TOF) 1233.4979 $(M + H)^+$ calcd for $C_{58}H_{69}N_{14}O_{17}$ 1233.4960.

Synthesis of **S10**

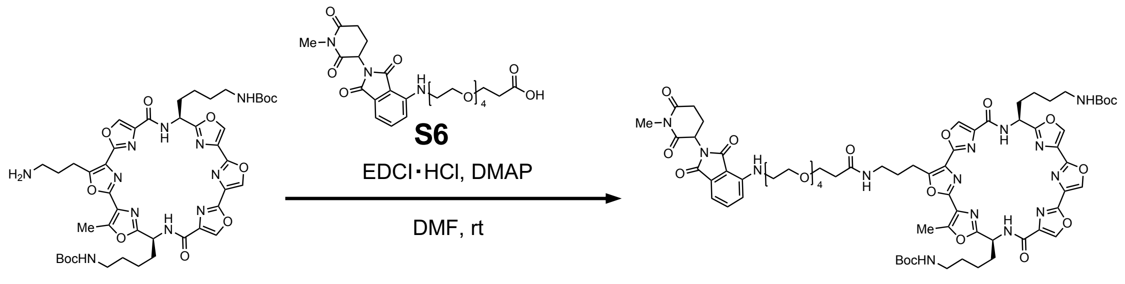

S2 **S10**

To a solution of **S2** (13.9 mg, 0.015 mmol) in CH_2Cl_2-THF (3 mL) was added EDCI•HCl (14.4 mg, 0.075 mmol) and DMAP (9.0 mg, 0.075 mmol) at room temperature under Ar atmosphere. After stirring 30 min, the reaction was added **S6** (19 mg, 0.0354 mmol). After stirring overnight, the reaction was concentrated in vacuo with toluene for three times. The residue was purified by column chromatography on silica gel (chloroform/methanol = 80:1 to 50:1) and to give **S10** as a yellow solid (16.0 mg, 0.010 mmol, 69% yield).

^{1}H-NMR (500 MHz, DMSO-d_6) δ 9.11 (s, 1H), 9.08 (s, 1H), 8.90 (s, 1H), 8.85 (s, 1H), 8.30 (dd, J = 19.5, 6.9 Hz, 2H), 7.94 (s, 1H), 7.69 (d, J = 10.3 Hz, 2H), 7.55 (t, J = 7.7 Hz, 1H), 7.10 (d, J = 8.6 Hz, 1H), 7.00 (d, J = 6.9 Hz, 1H), 6.77 (s, 2H), 6.55 (s, 1H), 5.40 (d, J = 5.2 Hz, 1H), 5.32 (t, J = 6.0 Hz, 1H), 5.10-5.05 (m, 1H), 4.16-4.10 (m, 2H), 3.60-3.44 (m, 23H), 3.17-3.10 (m, 4H), 2.99-2.70 (m, 12H), 2.28 (t, J = 6.3 Hz, 2H), 2.04 (s, 3H), 1.89 (t, J = 6.6 Hz, 3H), 1.62 (d, J = 5.7 Hz, 1H), 1.33-1.22 (m, 39H), 0.87 (t, J = 7.2 Hz, 6H); ^{13}C-NMR (100 MHz, DMSO-d_6) δ 171.8, 170.1, 169.8, 168.9, 167.2, 164.5, 162.0, 158.8, 158.7, 155.6, 155.5, 154.5, 153.9, 151.5, 146.4, 142.4, 142.2, 141.0, 136.2, 136.0, 135.9, 132.0, 129.8, 128.4, 124.6, 123.7, 117.4, 110.6, 109.1, 79.2, 77.2, 69.7, 69.6, 69.5, 68.8, 66.8, 49.1, 47.3, 41.7, 37.8, 36.3, 36.1, 36.0, 33.4, 31.1, 29.1, 28.1, 27.3, 27.2, 26.9, 26.6, 22.9, 21.3, 21.1, 20.9, 11.5; HRMS (ESI-TOF) 1469.6015 $(M + Na)^+$ calcd for $C_{69}H_{86}N_{14}NaO_{21}$ 1469.5984.

Synthesis of **2b**

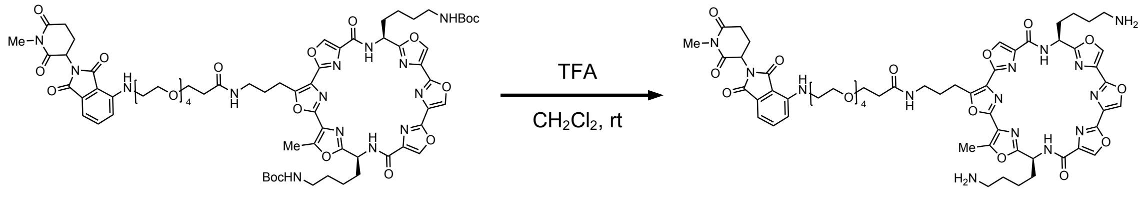

S10 **2b**

To a solution of **S9** (18 mg, 0.012 mmol) in CH_2Cl_2 (1 mL) was added TFA (0.5 mL) at room temperature. After stirring for 30 min, the reaction was concentrated in vacuo with toluene for three times to give **2b** as a yellow solid (17 mg, 0.0049 mmol, 96% yield).

^{1}H-NMR (400 MHz, DMSO-d_6) δ 9.14-9.12 (m, 2H), 8.93 (s, 1H), 8.88 (s, 1H), 8.30-8.26 (m, 2H), 7.95 (t, J = 5.3 Hz, 1H), 7.65-7.54 (m, 10H), 7.11 (d, J = 8.7 Hz, 1H), 7.01 (d, J = 6.9 Hz, 1H), 6.57 (d, J = 17.4 Hz, 1H), 5.43 (dd, J = 12.6, 5.3 Hz, 1H), 5.34 (t, J = 5.7 Hz, 1H), 5.11-5.05 (m, 1H), 3.60-3.44 (m, 30H), 3.16-3.13 (m, 6H), 2.99-2.88 (m, 6H), 2.80-2.70 (m, 11H), 2.05 (d, J = 5.5 Hz, 4H), 1.90 (q, J = 7.2 Hz, 5H), 1.54-1.44 (m, 8H), 1.23 (d, J = 7.3 Hz, 2H); ^{13}C-NMR (100 MHz, DMSO-d_6) δ 172.4, 170.6, 170.4, 169.4, 167.7, 164.9, 162.5, 159.4, 159.3, 158.6, 158.3, 156.2, 156.0, 155.1, 154.5, 152.1, 146.9, 143.1, 142.9, 142.3, 141.6, 136.7, 136.5, 136.3, 132.5, 130.2, 129.0, 125.1, 124.3, 118.0, 116.3, 111.2, 109.6, 79.7, 70.3, 70.2, 70.0, 69.4, 67.3, 49.6, 47.7, 47.6, 42.3, 42.2, 39.2, 38.8, 38.4, 38.3, 38.0, 36.7, 33.9, 31.6, 28.0, 27.7, 27.3, 27.1, 26.7, 23.4, 21.9, 21.4, 21.4, 12.1; HRMS (ESI-TOF) 1247.5091 $(M + H)^+$ calcd for $C_{59}H_{71}N_{14}O_{17}$ 1247.5116.

2. General experimental procedures for biochemistry experiments. All non-labelled and biotin-labeled oligonucleotides were purchased from Eurofin or IDT (HPLC purification grade) and used without further purification. The fluorescent measurement was performed using the Spark (TECAN). Curve fitting was carried out in GraphPad Prism 9. Membranes images were acquired on ChemiDoc XRS (Bio-Rad). Image analysis was performed using ImageJ (National Institutes of Health, USA).

Fluorescence polarization assay

Fluorescent polarizations were recorded on the Spark (TECAN) using 384-well plate (Greiner). The excitation wavelength was 415 nm, and the emission wavelength was 520 nm. Oligonucleotides (purchased by Eurofin or IDT) were used in this protocol, and were dissolved as stock solutions (100 μM) in MilliQ water to be used without further purification. Further dilutions of the oligonucleotides were performed with 10 mM Li-cacodylate buffer (pH 7.4) with 100 mM KCl, and this experiment was carried out with a 1.5 μM DNA or RNA solution. The solution was denatured at 95ºC for 5 min, and then slowly cooled to room temperature. Oligonucleotide solutions diluted to 19–1500 nM using 10 mM Li-cacodylate buffer (pH 7.4) with 100 mM KCl. The compounds (G4L-PROTAC) were prepared as DMSO stock solutions (10 mM). The compounds solutions diluted to 0.4 μM using 10 mM Li-cacodylate buffer (pH 7.4) with 100 mM KCl. Each solution containing each concentration of oligonucleotide (55 μL) was added into the compound samples (55 μL) and incubated overnight. The *K*d values were calculated by using the following formula. Since a single G4 molecule can bind two ligands, the nucleic acid concentration was intentionally set to twice its actual value during data fitting for analysis.

$$\mathrm{Y} = \frac{Y_{max} \cdot ([\mathrm{ligand}] + [\mathrm{binding\ site}] + \mathrm{Kd} - \sqrt{([\mathrm{ligand}] + [\mathrm{binding\ site}] + \mathrm{Kd})^2 - 4 \cdot [\mathrm{ligand}] \cdot [\mathrm{binding\ site}]}}{2 \cdot [\mathrm{ligand}]}$$

Thiazole Orange (TO) displacement assay

TO displacement assay was performed with 96-well plate by an excitation wavelength of 485 nm and a detection wavelength of 535 nm using the Spark (TECAN, Switzerland, Zurich). Oligonucleotides (purchased by Eurofin or IDT) were used in this protocol, and were dissolved as stock solutions (100 μM) in MilliQ water to be used without further purification. Further dilutions of the oligonucleotides were performed with 10 mM Li-cacodylate buffer (pH 7.4) with 100 mM KCl, and this experiment was carried out with a 2.0 μM DNA or RNA solution. The solution was annealed by heating at 95°C for 5 min, and then slowly cooled to room temperature. The compounds (L2H2-6OTD, G4L-PROTAC and TO) were prepared as DMSO stock solutions (10 mM). TO solution diluted to 10 μM using MQ and L2H2-6OTD and G4L-PROTAC solutions diluted to 0-2 mM using DMSO. The TO solution (10 μM) was added into the annealing RNA solution, to give concentrations of the

oligonucleotide (0.513 μM) and the TO (1.03 μM), and incubated for 30 min. After that, each concentration of compound solutions (1 μL) was added into the mixture samples (39 μL in Li-cacodylate buffer with 100 mM KCl) and incubated at 25°C for 1 h (Final concentration = Oligonucleotides: 0.5 μM, TO: 1.0 μM, L2H2-6OTD or G4L-PROTAC compound: 0-50 μM, DMSO = 2.5%). Finally, we measured the fluorescent of TO at least three times at 25°C, and calculated EC_{50} values (the concentra-tion to displace 50% TO from oligonucleotide) of each compound, respectively. The spectra are representative of three averaged scans taken. The data were plotted following the formula.

$$E_x = 100 \times \left(1 - \frac{F_x}{F_o}\right)$$

The EC_{50} were calculated using the built-in nonlinear regression (sigmoidal, 4PL, X is concentration) by Graphpad Prism 9.

CRBN binding assay

This experiment was performed using the Cereblon binding kit (64BDCRBNPEG) purchased from cisbio. The excitation wavelength was 320 nm, the detection wavelength was 620, 665 nm, and the TR-fluorescence intensity was measured using the Spark (TECAN). The original compounds were prepared as DMSO stock solutions (10 mM) and diluted to 0.1, 0.3, 1, 3, 10 or 30 μM in DMSO. The remaining dilutions were made in diluent. The CRBN binding assay was conducted using a 0.04, 0.12, 0.4, 1.2, 4 or 12 μM solution of the compounds. Next, the compound solution (5μL), Human CRBN WT GST-tagged (5μL), GST Eu Cryptate antibody (5 μL) and thalidomide-red reagent (5 μL) were mixed on the 384-well proxiplate. The plate was then incubated for 3 hours at 25ºC. Subsequently, the TR-fluorescence intensity was recorded and the FRET-Ratio of acceptor to donor emission signals for each well was calculated using the following formula.

$$\text{FRET-Ratio} = \frac{F_{665}}{F_{620}} \times 10^4$$

The DC_{50} were calculated using the built-in nonlinear regression (sigmoidal, 4PL, X is concentration) by Graphpad Prism 9.

Quaternary complex formation assay

This experiment was performed using the Cereblon binding kit (64BDCRBNPEG) purchased from cisbio. This protocol used biotin-labeled oligonucleotides. Purified nucleotides were dissolved in MilliQ water as stock solutions (100 μM) and used as is. The oligonucleotides were further diluted in cacodylate buffer (50 mM Tris-HCl, pH 7.4, 150 mM KCl), and this experiment was conducted using an oligonucleotide solution (4 μM). The biotin-labeled oligonucleotide was annealed by heating at 95°C for 5 minutes and then cooling to room temperature. The duplex DNA was prepared by mixing

biotin-labeled telo24 and unlabeled ssDNA to create a solution, which was then annealed under the same conditions. Then, we mixed biotin-labeled oligonucleotide (1.2 μM) and d2-streptavidin (20 μg/μL, 610SADLF) in Tris buffer and incubated for 3 hours at 25°C. **1a**–**2b** were prepared as DMSO stock solutions (10 mM) and diluted to 1, 3 or 10 μM in DMSO. The remaining dilutions were made in diluent. This assay was conducted using a 0.4, 1.2 or 4 μM solution of the compounds. Next, the compound solution (5μL), Human CRBN WT GST-tagged (5μL), GST Eu Cryptate antibody (5 μL), PROTAC binding buffer (3 μL) and oligonucleotide-streptavidin solution (2 μL) were mixed on the 384-well proxiplate. The plate was then incubated for 3 hours at 25°C. Subsequently, the TR-fluorescence intensity was recorded with the Spark (TECAN) and the DFRET-Ratio was calculated using the following formula.

$$\Delta\text{FRET-Ratio} = \text{FRET-Ratio}_{\text{ligand}}\text{-FRET-Ratio}_{\text{w/o ligand}}$$

Cell culture

Hela cells were cultured at 37°C, 5% CO_2 in DMEM (SHIMADZU) containing 4.5 g/L glucose, 10% heat-inactivated fetal bovine serum (FBS, Corning), 1% penicillinstreptomycin and 0.1% sodium bicarbonate. 12 hours prior to compound addition, cells were seeded in 12-well plates at 1.5×10^5 cells/well and placed in a 37°C, 5 % CO_2 atmosphere.

Western blotting

Hela cells were treated with 3, 10 or 30 μM of G4L-PROTAC together with Endo-Porter peg (6 μM) for 24 hours. Then, the Hela cells were washed twice with ice-cold phosphate-buffered saline (PBS). SDS buffer was added, and cells were lysed. The cell lysate denatured at 96°C for 2 minutes, and protein concentration was quantified using Quick Start™ Braford Dye Reagent (BIO-RAD). 30 μg of total protein were separated by 10% SDS-PAGE gel electrophoresis (80 V: 20 min, 200 V: 30 min), transferred onto a PVDF membrane. The membrane was blocked in 4% skim milk in tris-buffered saline with 0.1% Tween20 (TBS-T) for 1 hour at room temperature or overnight at 4°C before incubation with primary antibody for 1hour at room temperature. After incubation with the peroxidase-conjugated secondary antibody for 1 hour at room temperature, the bands visualized using ChemiDoc XRS (Bio-Rad). Image analysis was performed using ImageJ 1.47c (National Institutes of Health, USA). The following antibodies were used in this study: anti-DHX36 (Bethyl Labs, #A300-525A; 1:5,000 dilution), anti-GAPDH (Proteintech, #10494-1-AP; 1:20,000 dilution), anti-Nucleolin (MBL, M019-3: 1:1,000 dilution) and anti-EDF1 (Proteintech, #12419-1-AP; 1:3,000dilution). Secondary antibodies were HRP-conjugated anti-rabbit IgG (Cell Signaling Technology, #7074; 1:3,000 dilution) and HRP-conjugated AffiniPure Goat Anti-mouse IgG (H+L) (Jackson ImmunoResearch, # 115-035-003; 1:50,000 dilution).

Real-time PCR analysis

Total RNA of Hela cells treated with G4L-PROTAC for 24 hours was extracted using RNeasy Plus Mini Kit (Qiagen) in accordance with the manufacturer's instructions. RNA was reverse transcribed using ReverTraAce qPCR RT Master Mix (TOYOBO) according to the manufacturer's protocol. Real-time PCR was performed on Thermal Cycler Dice Real Time System III (TaKaRa), using 10 μL of THUNDERBIRD SYBR qPCR Mix (TOYOBO), 1.2 μL of primers (5 μM), cDNA (1 μL) and Nuclease Free water for a total volume reaction of 20 μL. The reaction conditions were as follows: 95 °C for 30 s, followed by 40 cycles of 95 °C for 5 s and 60 °C for 30 s, and then followed by a melt curve stage of 95 °C for 15 s, 65 °C for 30 s, and 95 °C for 15 s. RPS18 used as internal housekeeping control gene. The $2^{-\Delta\Delta Ct}$ method was used to calculate gene expression fold-changes. Primer sequences were listed in Supplementary Table S2.

Cytotoxicity assay of G4L-PROTAC

Hela cells (12,000 cells, 80 μL) were suspended in DMEM 10% FBS and seeded in a 96-well plate and incubated for 12 hours. The cells were incubated with G4-PORTAC ligands were added in serial solutions at a volume of 20 μL per well at different concentrations and incubated with cells. After 24 hours, sodium 7-oxido-3-oxo-3H-phenoxazine 10-oxide (10 μL, 0.55 mM in PBS) was added to each well and the mixture incubated for an additional 4 hours. Fluorescent signal was scanned by plate reader (λex = 570 nm, λem = 585 nm). Error bars represent standard deviation from triplicate measurements.

Immunofluorescence staining

Place round 10 mm ø cover slides (24 sheets) in 24-well plate and add 0.5 mL cell solution (2 x 10^5 cells/mL for HeLa) and incubate 24h in the incubator with or without 30 μM G4L-PROTAC. Cells were fixed with 4% paraformaldehyde in PBS for 15 min at room temperature. After rinsing with PBS, cells were permeabilized with TritonX-100 in PBS for 10 min. After rinsing with PBS, all samples were blocked for 1 h with blocking solution (2% bovine serum albumin (BSA) in PBS). Cells were incubated overnight at 4ºC with BG4 (1:1000 dilution, Sigma-Aldrich ZMS1070). Cells were washed four times with PBS for 2 min at room temperature. Cells were incubated with goat anti-mouse Alexa 594 antibody (1:500 dilution, Invitrogen A-11005), for 1 h at room temperature, and then washed three times with PBS for 2 min at room temperature. Cells were stained for 15 min with 2 μM DAPI in PBS at room temperature, and then washed two times with PBS for 2 min at room temperature. Slides were mounted with glycerol at 60 °C, sealed with clear nail polish, and allowed to dry for 4 hours.

Dual luciferase assay

Dual luciferase assay was designed by cloning a human telomere G4 sequence, 5'- GATCT <u>TTA</u>

GGG TTA GGG TTA GGG TTA GGG TTA G-3', into the promoter region of the Renilla gene, while leaving the HSV TK promoter unaltered for Firefly expression (Fig. 6B) in a luciferase vector pC-KIT1 (Plasmid #118983, Addgene). As Renilla and Firefly genes encode distinct luciferases that convert different substrates into products emitting different colors, they can be quantified by analyzing the ratio of bioluminescence intensity of the respective product catalyzed by either Renilla or Firefly luciferase. To prepare plasmid, we used restriction enzymes NheI and BglII (NEB) to digest the plasmid vector pC-KIT1, followed by circularization of linearized plasmid with T4 DNA ligase (NEB) in presence of the human telomere G4 sequence mentioned above. The cloning was confirmed with EcoRI-HF (NEB) digestion in agarose gel electrophoresis, followed by DNA sequencing at the DNA Sequencing Facility, the University of Maine, Orono.

HEK293 cells (ATCC CRL-3216™) were cultured in DMEM media supplemented with 10% FBS (Gibco) at 37 °C and 5% CO_2. The maintained cells (4×10^4 cells/well) were seeded in 96-well plate (Corning® 96-well Clear Flat Bottom-3598). Using GenJet™ Plus transfection reagent (SignaGen Laboratories), cells were transfected with the DNA cloned vector (200 ng/well) with G4L-PROTAC ligands. Bioluminescence levels of the two luciferases were measured after 24 hours, following manufacture protocol of Pierce™ Renilla-Firefly Luciferase Dual Assay Kit (ThermoFisher Scientific-16185) in a bioluminescence detector (FLx800™ Fluorescence Microplate Reader, BioTek Instruments). At least three biological replicates were performed in HEK293T cells transfected with the G4 containing recombinant plasmid (Fig. 6B). Luminescence intensity ratios (Renilla/Firefly) were normalized to the control, in which cells carried the vector without any G4L-PROTAC ligands.

Proteome analysis

Hela cells were treated with 30 μM of G4L-PROTAC together with Endo-Porter peg (6 μM) for 24 hours. Cells were harvested as a pellet by centrifugation. Then, the pellet was washed twice with ice-cold phosphate-buffered saline (PBS). The process beyond this point was outsourced to Promega. Proteins were solubilized in 100 mM Tris (pH 8.0) buffer containing 4% SDS, 20 mM NaCl, and 10% acetonitrile (ACN) using a sealed ultrasonic homogenizer. Protein concentration was determined using a BCA assay, and the sample concentration was adjusted to 0.15 μg/μL with the same buffer. Sera-Mag SpeedBead Carboxylate-Modified Magnetic Particles (Hydrophilic) and Sera-Mag Carboxylate-Modified Magnetic Particles (Hydrophobic) (Cytiva) were mixed in a 1:1 (v/v) ratio, washed three times with distilled water, and resuspended in distilled water at a concentration of 8 μg solids/μL (SP3 beads). SP3 beads (20 μL) were added to the protein solution, followed by the addition of three times the sample volume of 1-propanol. The mixture was incubated at room temperature for 20 minutes with gentle mixing. After incubation, the beads were washed twice with 80% 1-propanol and once with ethanol. The beads were then resuspended in 80 μL of 50 mM Tris-HCl (pH 8.0) containing 10 mM $CaCl_2$ and 0.02% LMNG (Lauryl Maltose Neopentyl Glycol). Proteins were digested with 1 μg

Trypsin Platinum (Promega) at 37°C for 14 hours. To reduce and alkylate disulfide bonds, the digested samples were treated with 10 mM TCEP and 40 mM 2-chloroacetamide at 80°C for 15 minutes. The reaction was quenched by adding 16 μL of 5% TFA, followed by vortexing. Peptides were desalted using a reverse-phase spin column (GL-Tip SDB, GL Sciences), dried using a centrifugal evaporator, and reconstituted in 0.1% TFA containing 0.01% DMNG (Decyl Maltose Neopentyl Glycol) by vortexing for 10 minutes. Peptide concentration was measured using the Fluorometric Peptide Assay (Thermo Scientific, Cat# 23290) and adjusted to 100 ng/μL with 0.1% TFA and 0.01% DMNG. Peptides were analyzed by nanoLC-MS for proteome profiling.

NanoLC–MS/MS analyses were performed on an UltiMate 3000 TSLCnano LC System (Thermo Fisher Scientific) and Q Exactive HF-X (Thermo Fisher Scientific), as described above. The injection volume was 200 ng. The mobile phases consisted of (A) 0.1% formic acid and (B) 0.1% formic acid and 80% acetonitrile. Gradient conditions were 8-37% B in 32 min, 37-75% B in 6 min, and 75% B in 2 min. The mass scan ranges were m/z 495–745. The normalized collision energy was set to be 23. Isolation window: 6 m/z. Minimum AGC target: 3.00e6.

All data were analysed by using DIS-NN. The Sequest search parameters were set as follows: parent ion tolerance 10 ppm. To estimate the number of false positive protein identifications in a systematic fashion, the target-decoy approach was performed by searching both the target protein sequences and the reversed protein sequence in the database searching process. The identification data were filtered to a 1% false discovery rate (FDR) using Percolator algorithm in Proteome Discoverer software platform. The proteins and peptides denoted with high FDR confidence (FDR<1%) were exported and used for later analysis. To ensure reliability of protein quantification, only proteins identified with more than 10 unique peptides were analyzed.

Oligonucleotides sequences

Table S1. Sequences of Oligonucleotides used in this paper

Name	ODN	Sequence
telo24	DNA	d[TTAGGGTTAGGGTTAGGGTTAGGG]
bcl2	DNA	d[GGGCGCGGGAGGAATTGGGCGGG]
VEGFR	DNA	d[GGGTACCCGGGTGAGGTGCGGGGT]
PARP1	DNA	d[TGGGGTCCGAGGCGGGGCTTGGG]
19wt	DNA	d[GGGGGAGGGGTACAGGGGTACAGGGG]
c-kit1	DNA	d[AGGGAGGGCGCTGGGAGGAGGG]
VEGF	DNA	d[CGGGGCGGGCCTTGGGCGGGGT]
KRAS	DNA	d[AGGGCGGTGTGGGAATAGGGAA]
NRAS	RNA	d[UGUGGGAGGGGCGGGUCUGGGUGC]
TERRA	RNA	d[UAAGGGUAAGGGUAAGGGUAAGGG]
ssDNA	DNA	d[CCCTAACCCTAACCCTAACCCTAA]
dsDNA	DNA	d[TATAGCTATATTTTTTATAGCTATA]
Biotin-telo24	DNA	Biotin-d[TTAGGGTTAGGGTTAGGGTTAGGG]
Biotin-NRAS	RNA	Biotin-d[UGUGGGAGGGGCGGGUCUGGGUGC]
Biotin-ssDNA	DNA	Biotin-d[CCCTAACCCTAACCCTAACCCTAA]

Table S2. Primers for Real-time PCR used in this study

Name		Sequences
DHX36	forward	d[GTTTAAATCAGTTAACCAGACAC]
	reverse	d[ACTGAACCTGACCGTACACGCAATGTTGGTAGCAATTA]
RPS18	forward	d[TGTGGTGTTGAGGAAAGCA]
	reverse	d[CTTCAGTCGCTCCAGGTCTT]

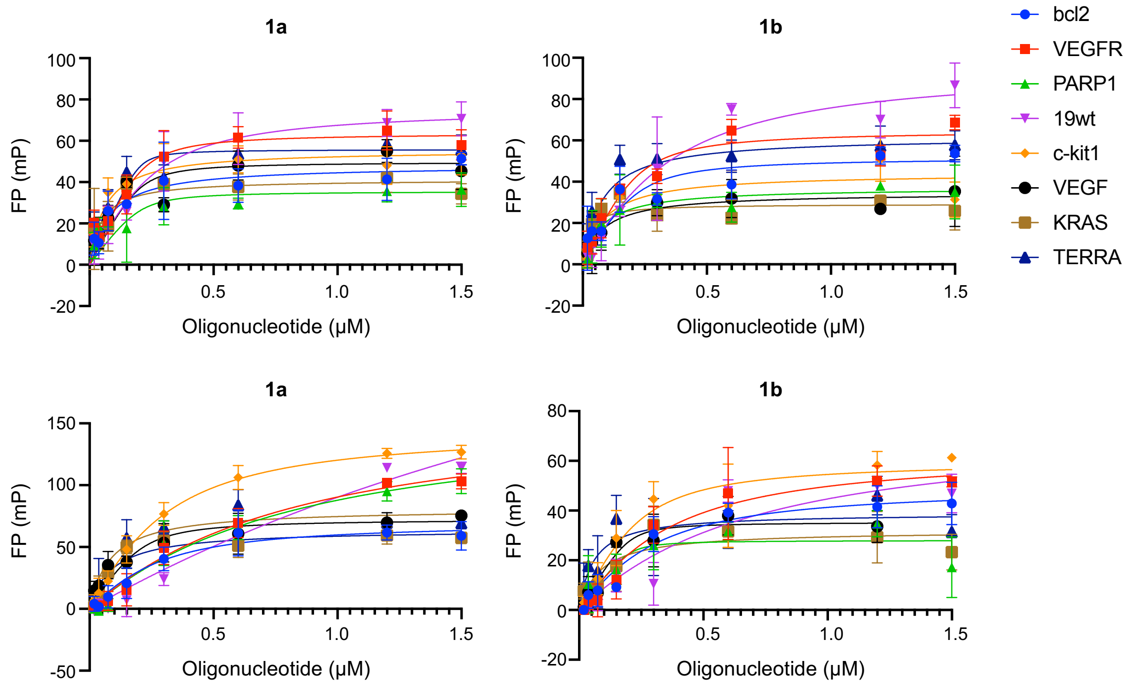

Figure S1. FP titration of **1a**–**2b** (0.2 μM) for several G4 sequences in 10 mM Li-cacodylate buffer (with 100 mM KCl, pH 7.4).

Table S3. K_d value (nM) for G4 sequences

Name	ODN	Topology	K_d (nM)			
			**1a**	**1b**	**2a**	**2b**
bcl2	DNA	Hybrid	30 ± 13	104 ± 53	266 ± 178	278 ± 258
VEGFR	DNA	Hybrid	27 ± 5	46 ± 18	802 ± 325	284 ± 139
PARP1	DNA	Hybrid	42 ± 40	39 ± 16	817 ± 447	101 ± 100
19wt	DNA	Anti-parallel	103 ± 27	289 ± 184	3402 ± 436	638 ± 255
c-kit1	DNA	Parallel	39 ± 23	38 ± 41	178 ± 22	84 ± 16
VEGF	DNA	Parallel	42 ± 38	4 ± 3	46 ± 23	43 ± 31
KRAS	DNA	Parallel	45 ± 35	19 ± 14	103 ± 45	99 ± 44
TERRA	RNA	Parallel	72 ± 39	26 ± 22	5 ± 4	110 ± 115

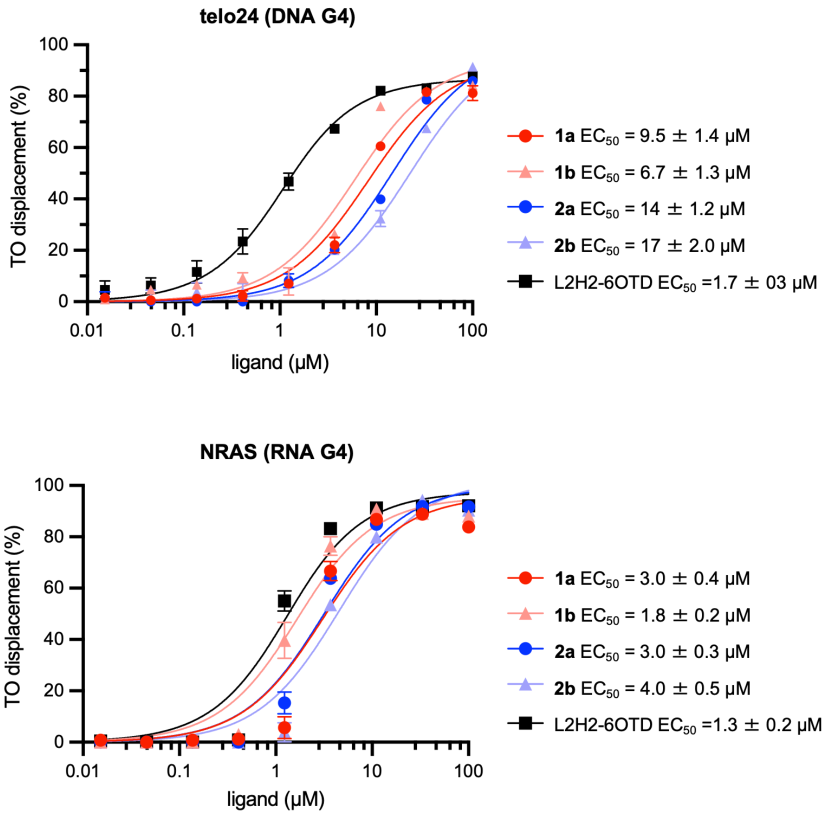

Figure S2. Thiazole-orange (TO) displacement assay. The EC_{50} values were calculated based on the TO fluorescence change after addition of each ligand (0–100 μM) to a mixture of telo24 or NRAS (0.5 μM) and TO (1 μM) in 10 mM Li-cacodylate buffer (with 100 mM KCl, pH 7.4). Data represents means ± SD (n = 3).

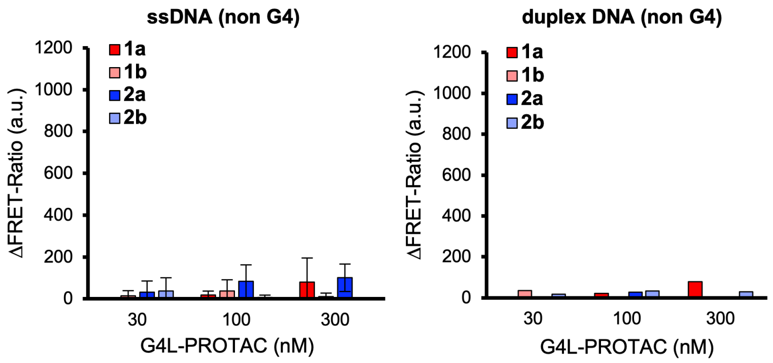

Figure S3. Verification of ternary complex formation using non-G4 (ssDNA and duplex DNA) with TR-FRET. The values represent the mean ± S.D. from three independent experiments (n = 3).

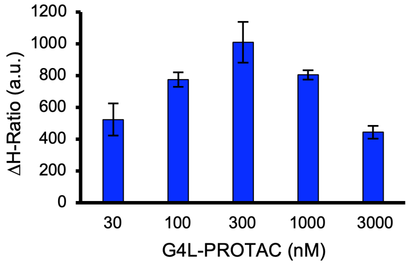

Figure S4. Verification of ternary complex formation using G4 (telo24) and **2a** (30–3000 nM) with TR-FRET.

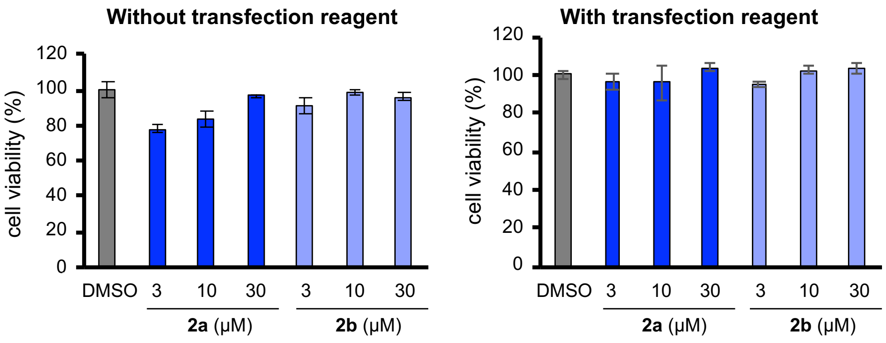

Figure S5. Cell viability was assessed using the AlamarBlue assay in HeLa cells treated with **2a** or **2b** for 24 hours, in the presence or absence of Endo-Porter PEG (0 or 6 μM), under all conditions used in this study. The values represent the mean ± S.D. from three independent experiments (n = 3).

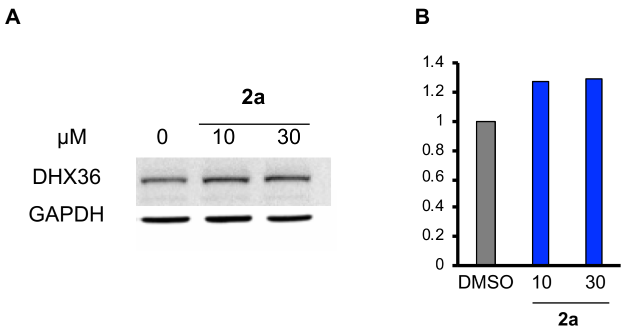

Figure S6. Western-blotting analysis of DHX36 in Hela cells treated with **2a** or **2b** for 24 hours without Endo-Porter PEG.

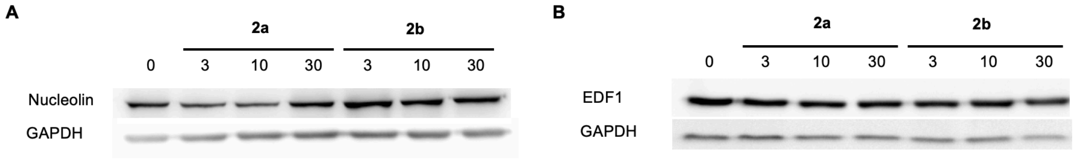

Figure S7. Western blotting analysis of Nucleolin (A) and EDF1 (B) in Hela cells treated with **2a** or **2b** for 24 hours.

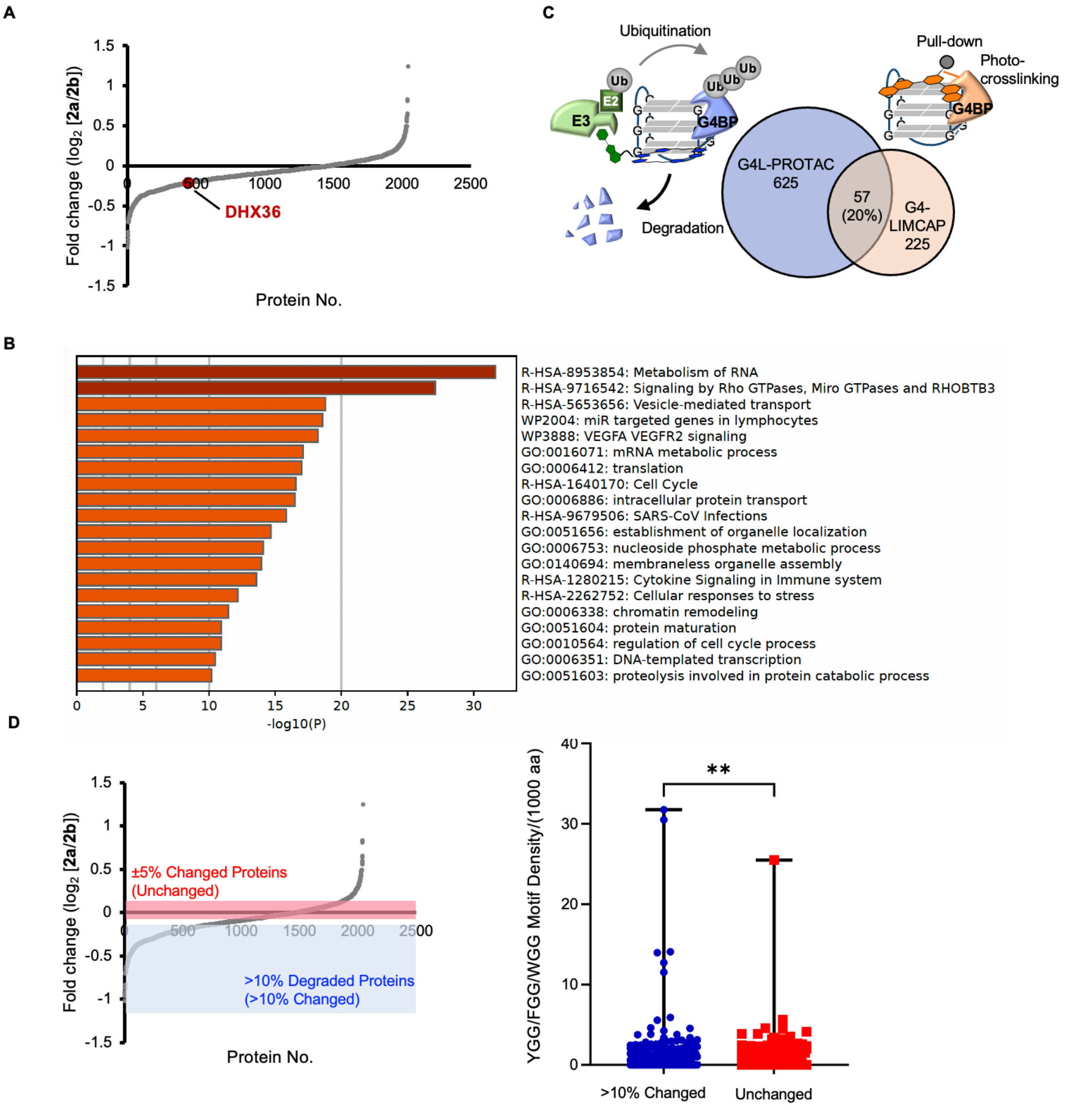

Figure S8. (A) Log₂ fold-change plot of proteins identified in HeLa cells upon **2a** versus **2b** treatment (n =2). Each dot represents a protein, and the y-axis indicates the relative abundance ratio (**2a**/**2b**). (B) Gene ontology (GO) analyses of the 682 proteins downregulated by >10% upon **2a** treatment. (C) Venn diagram showing the overlap between proteins reduced by the G4L-PROTAC ligand **2a** and G4-related proteins (G4RPs) previously identified by G4-LIMCAP. (D) Box-and-whisker plot comparing the density of short aromatic-glycine motifs (YGG, FGG, and WGG) per 1,000 amino acids between proteins downregulated by more than 10% after **2a** treatment (blue) and proteins with minimal change (±5%, red). $^{**}P < 0.01$.

Whole membrane images Figure S7A, B

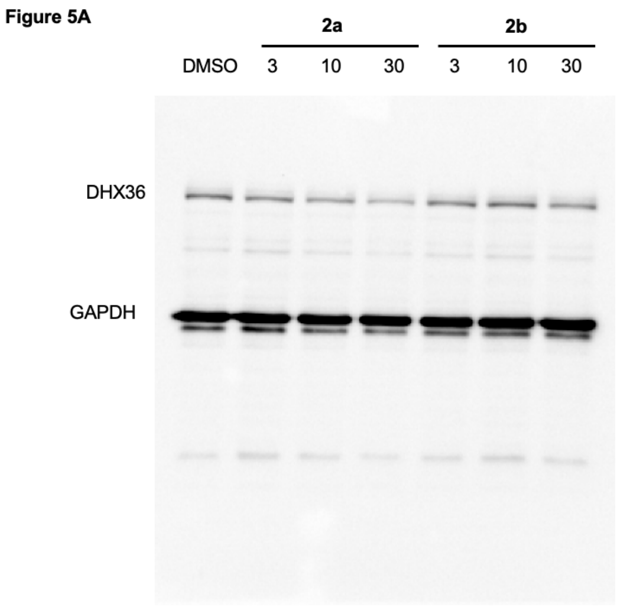

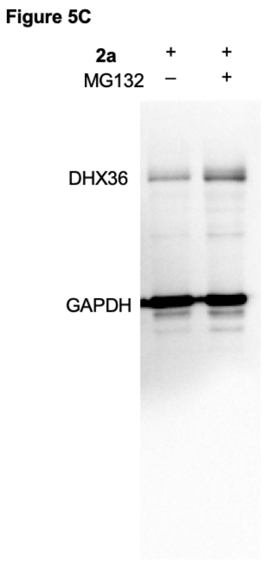

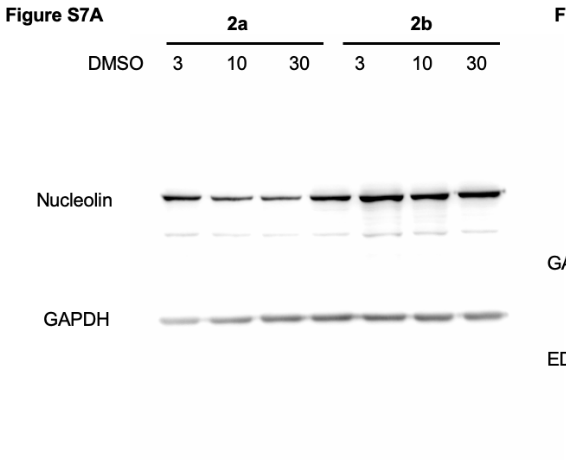

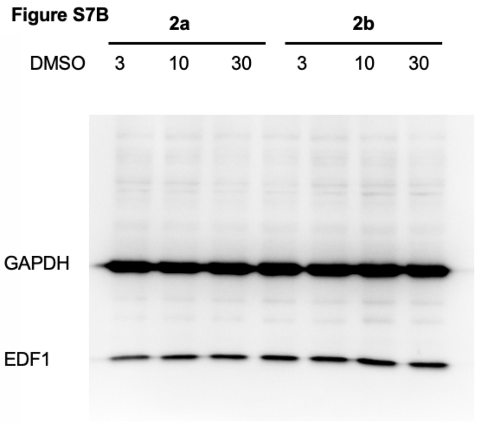

^{1}H and ^{13}C NMR spectra

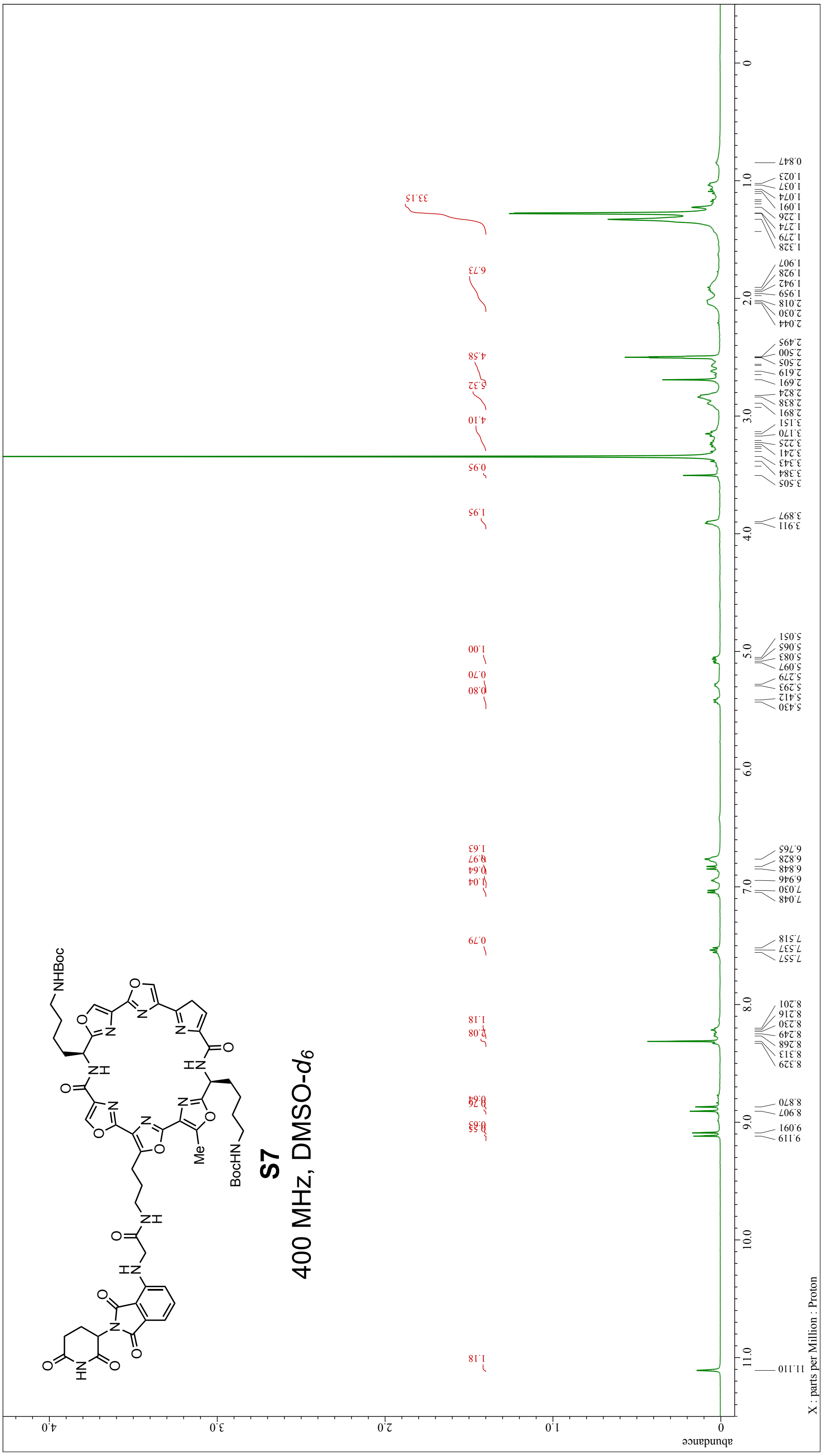

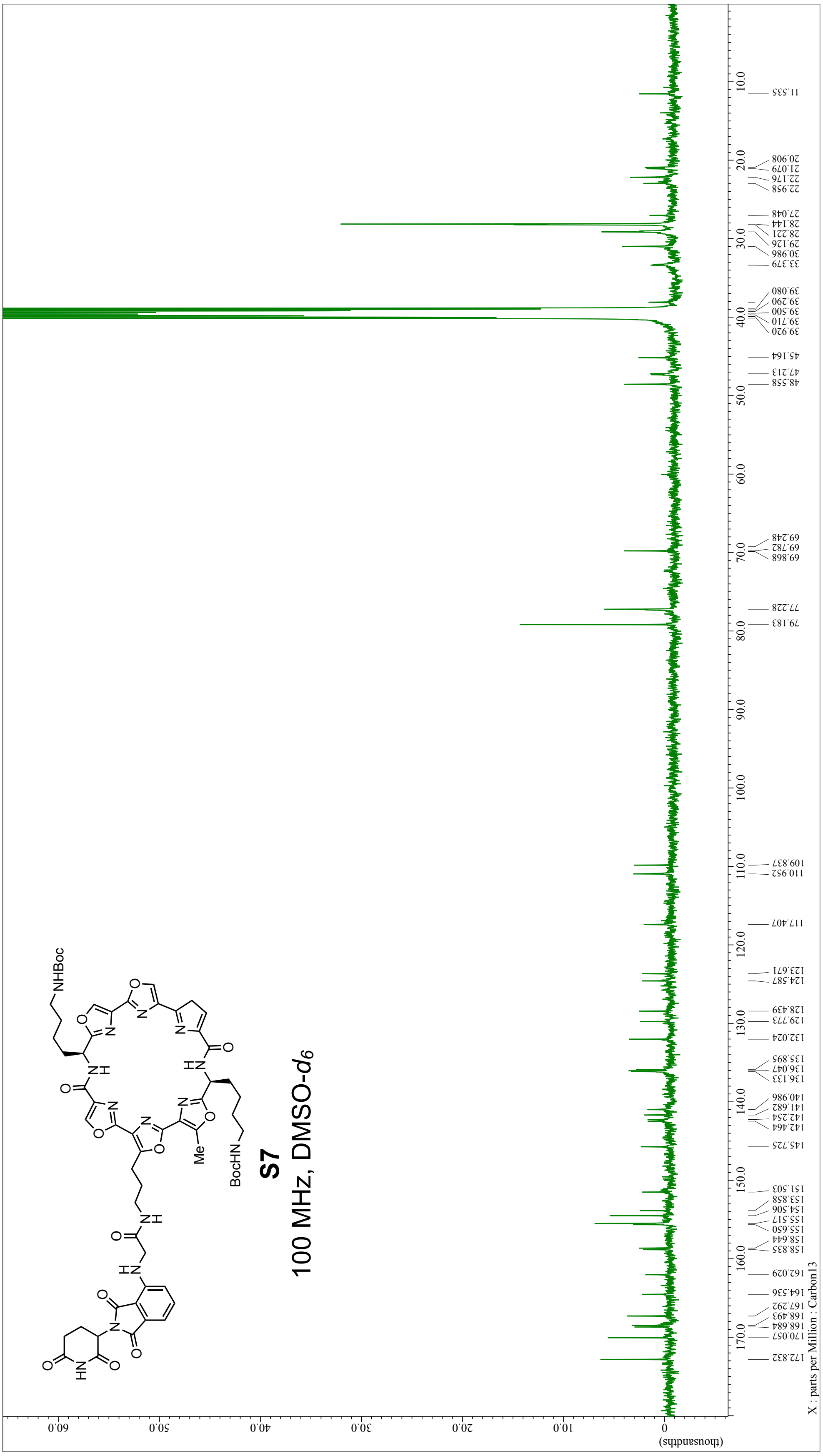
S7
100 MHz, DMSO-d_6
NHBoc
BocHN
Me
X : parts per Million : Carbon13
(thousandths)
11.535
20.908
21.079
22.176
22.958
27.048
28.144
28.221
29.126
30.986
33.379
39.080
39.290
39.500
39.710
39.920
45.164
47.213
48.558
69.248
69.782
69.868
77.228
79.183
109.837
110.952
117.407
123.671
124.587
128.439
129.773
132.024
135.895
136.047
136.133
140.986
141.682
142.254
142.464
145.725
151.503
153.858
154.506
155.517
155.650
158.644
158.835
162.029
164.536
167.292
168.493
168.684
170.057
172.832

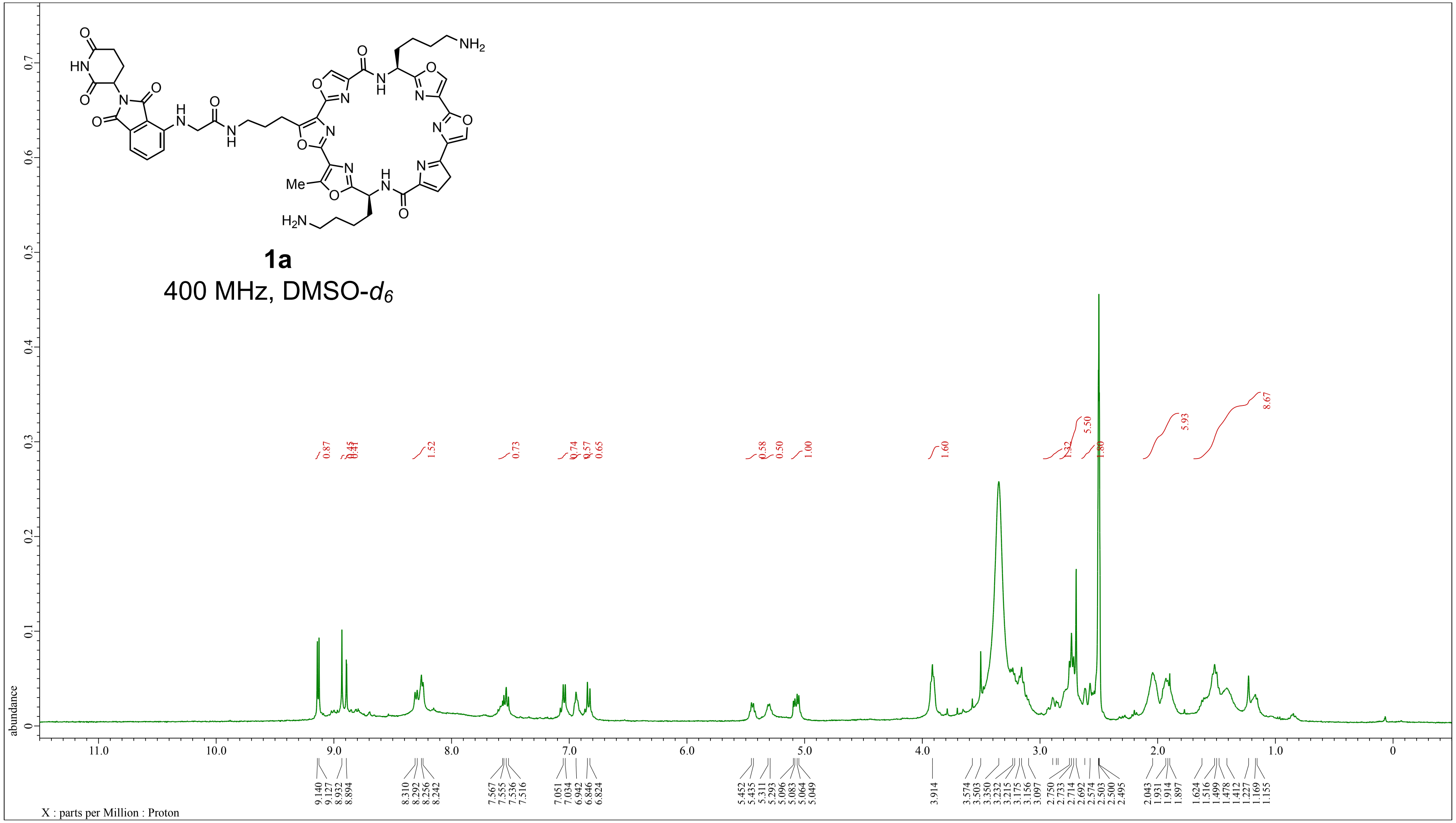
1a
400 MHz, DMSO-d_6
abundance
X : parts per Million : Proton

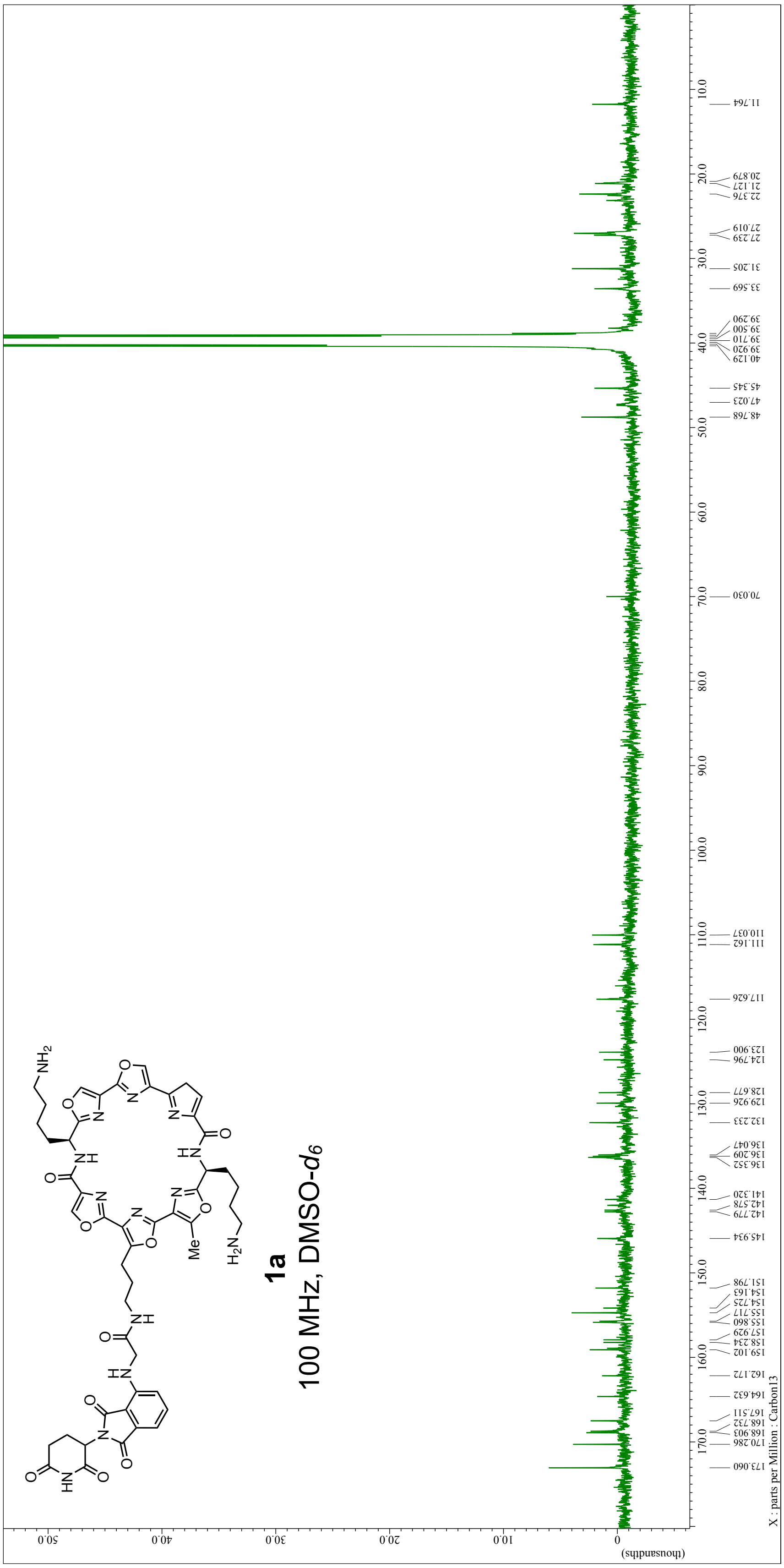
1a
100 MHz, DMSO-d_6
X : parts per Million : Carbon13
(thousandths)
173.060
170.286
168.903
168.732
167.511
164.632
162.172
159.102
158.234
157.929
155.860
155.717
154.725
154.163
151.798
145.934
142.779
142.578
141.320
136.352
136.209
136.047
132.233
129.926
128.677
124.796
123.900
117.626
111.162
110.037
70.030
48.768
47.023
45.345
40.129
39.920
39.710
39.500
39.290
33.569
31.205
27.239
27.019
22.376
21.127
20.879
11.764

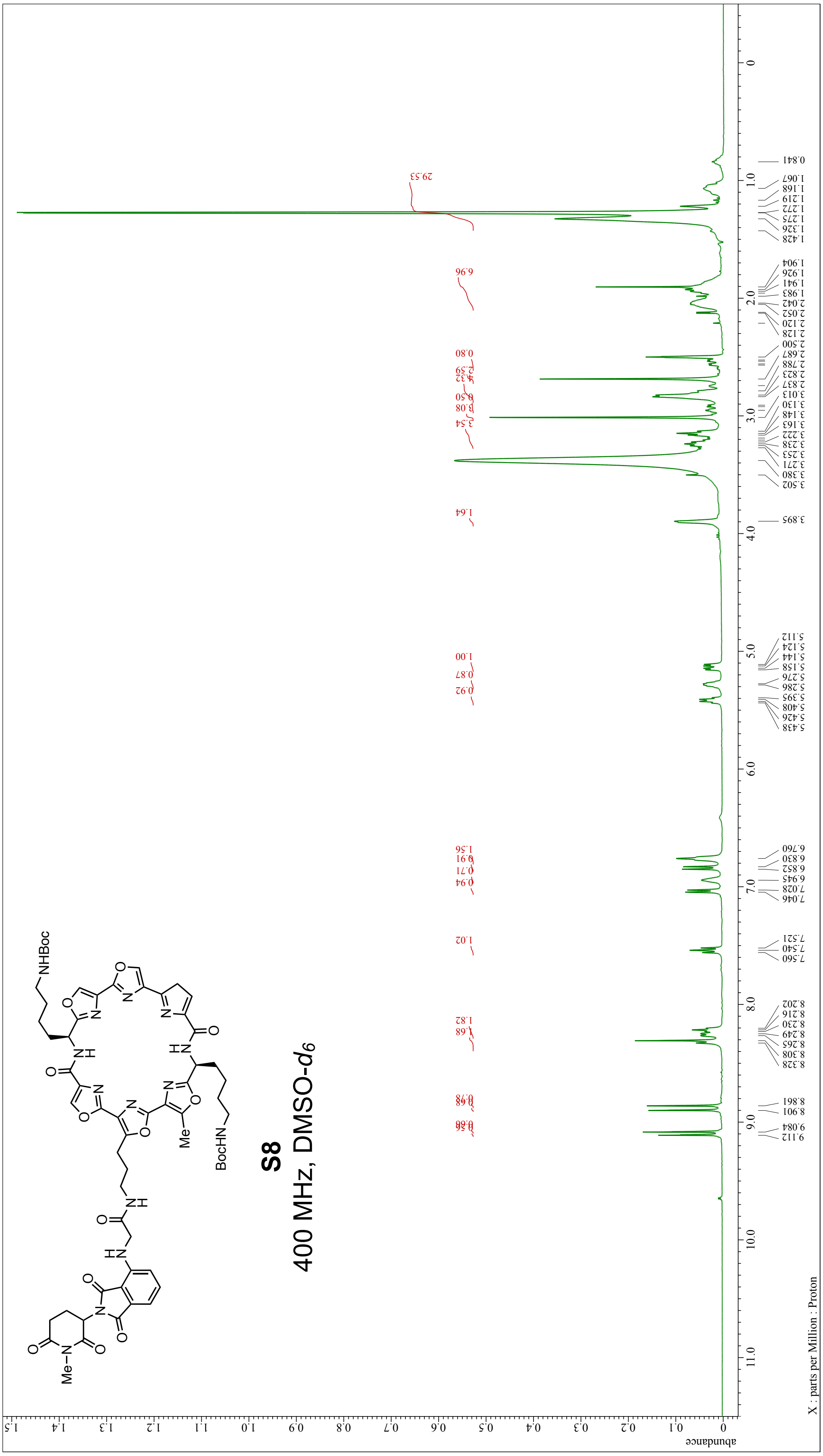
S8
400 MHz, DMSO-d6
X : parts per Million : Proton
abundance

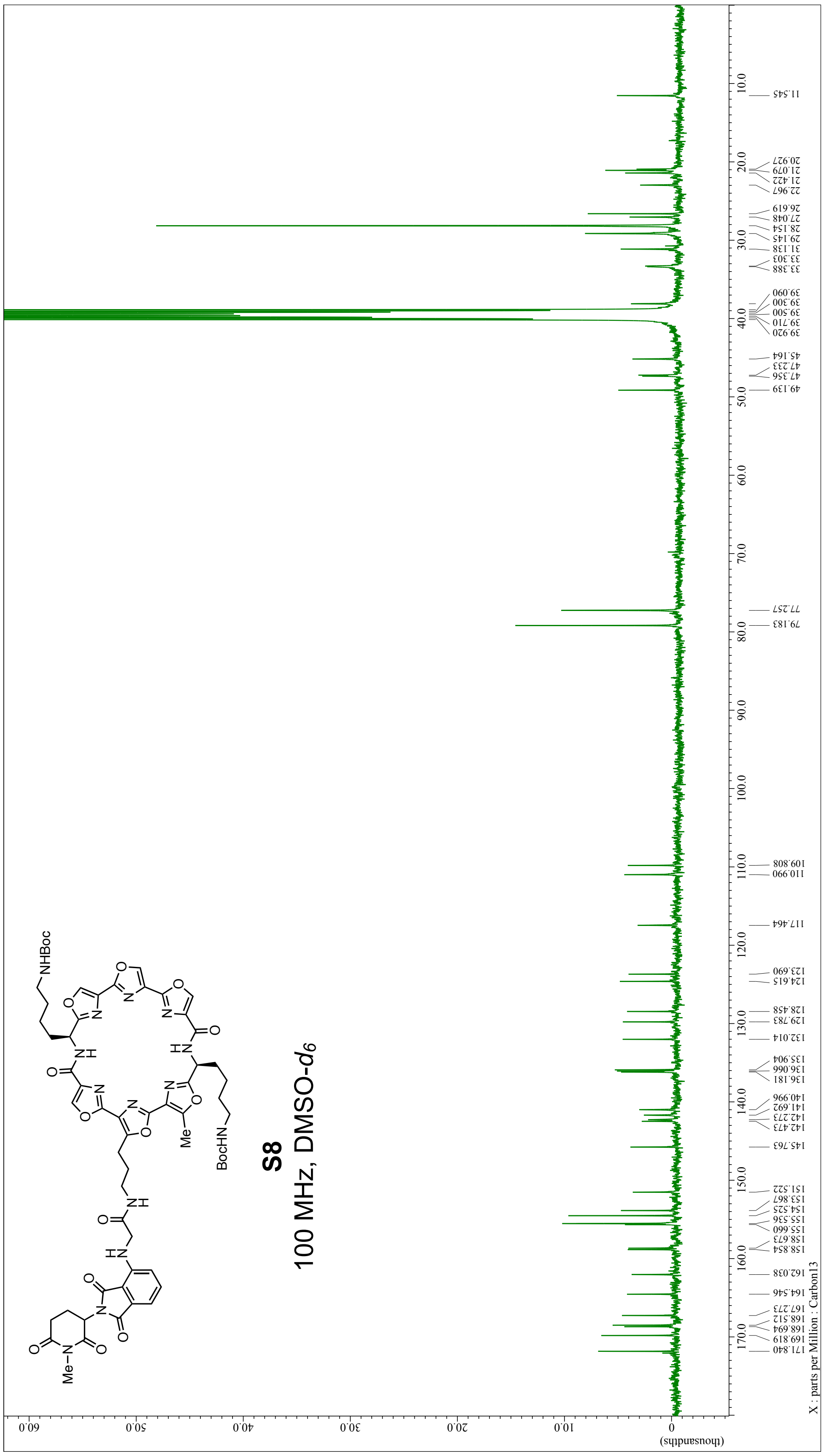
S8
100 MHz, DMSO-d_6
NHBoc
BocHN
Me
Me–N
X : parts per Million : Carbon13
(thousandths)
171.840
169.819
168.694
168.512
167.273
164.546
162.038
158.854
158.673
155.660
155.536
154.525
153.867
151.522
145.763
142.473
142.273
141.692
140.996
136.181
136.066
135.904
132.014
129.783
128.458
124.615
123.690
117.464
110.990
109.808
79.183
77.257
49.139
47.356
47.233
45.164
39.920
39.710
39.500
39.300
39.090
33.388
33.303
31.138
29.145
28.154
27.048
26.619
22.967
21.422
21.079
20.927
11.545

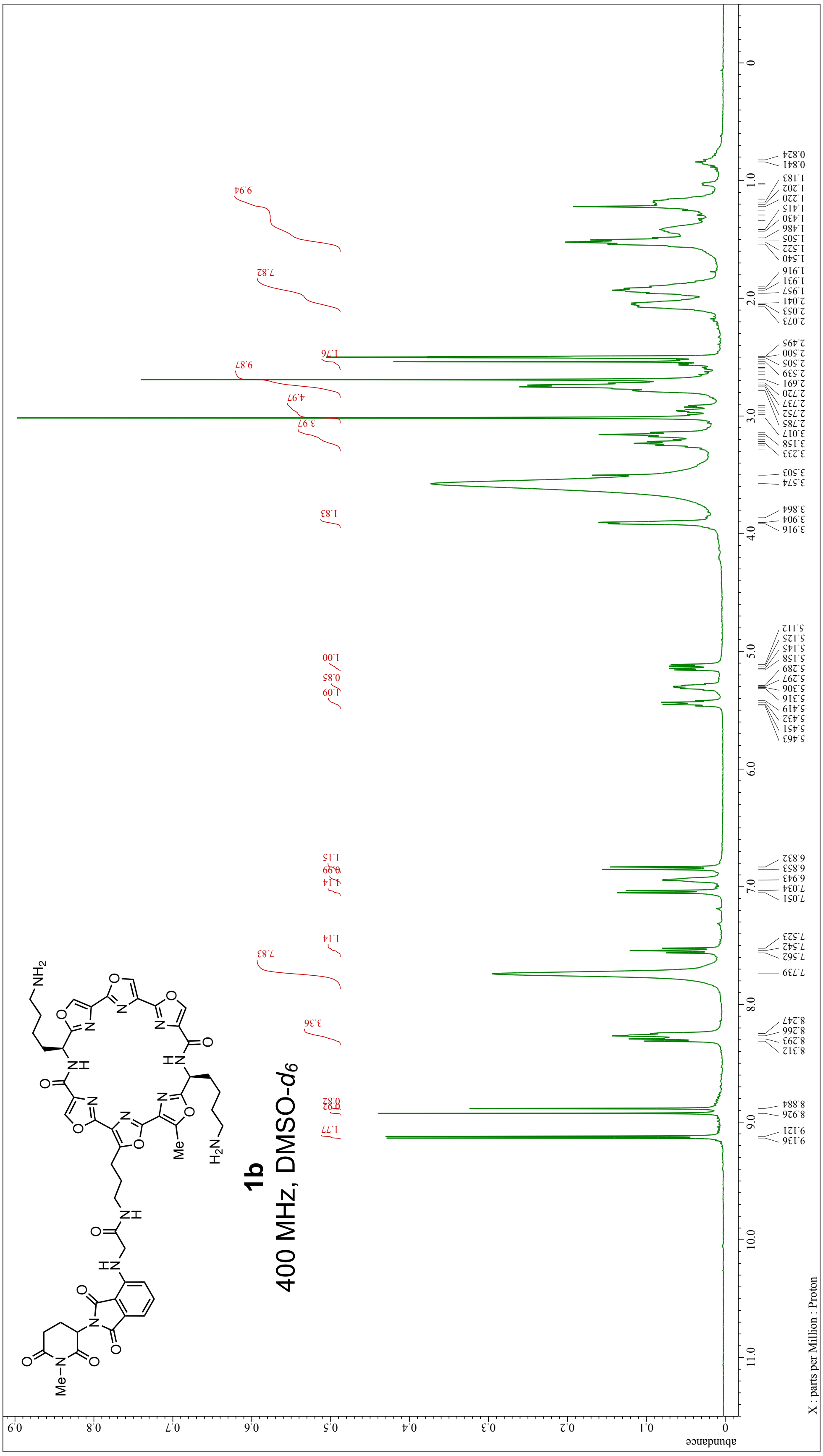
1b
400 MHz, DMSO-d_6
X : parts per Million : Proton
abundance

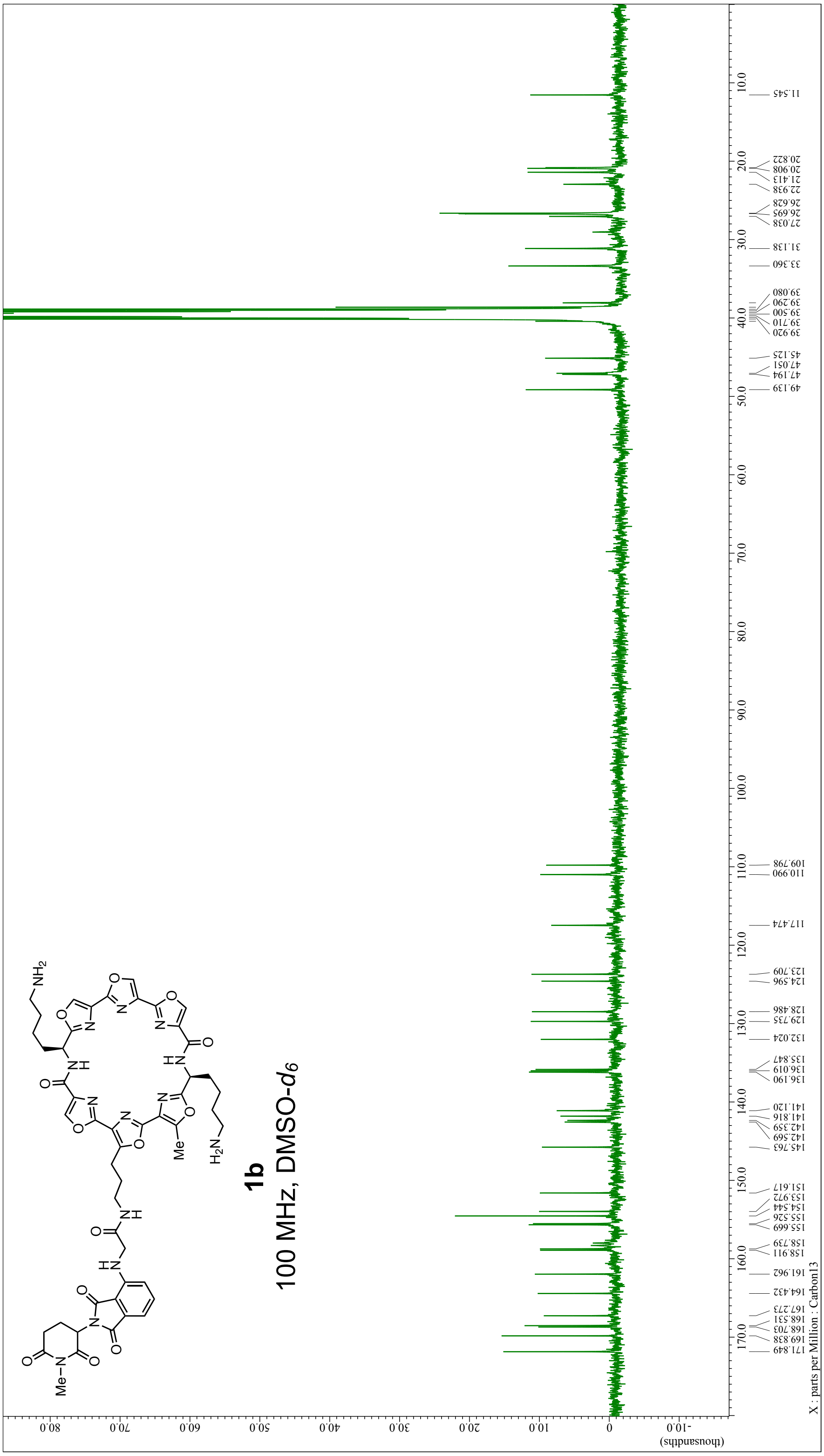
1b
100 MHz, DMSO-d_6
11.545
20.822
20.908
21.413
22.938
26.628
26.695
27.038
31.138
33.360
39.080
39.290
39.500
39.710
39.920
45.125
47.194
47.051
49.139
109.798
110.990
117.474
123.709
124.596
128.486
129.735
132.024
135.847
136.019
136.190
141.120
141.816
142.359
142.569
145.763
151.617
153.972
154.544
155.526
155.669
158.739
158.911
161.962
164.432
167.273
168.531
168.703
169.838
171.849
X : parts per Million : Carbon13
(thousandths)

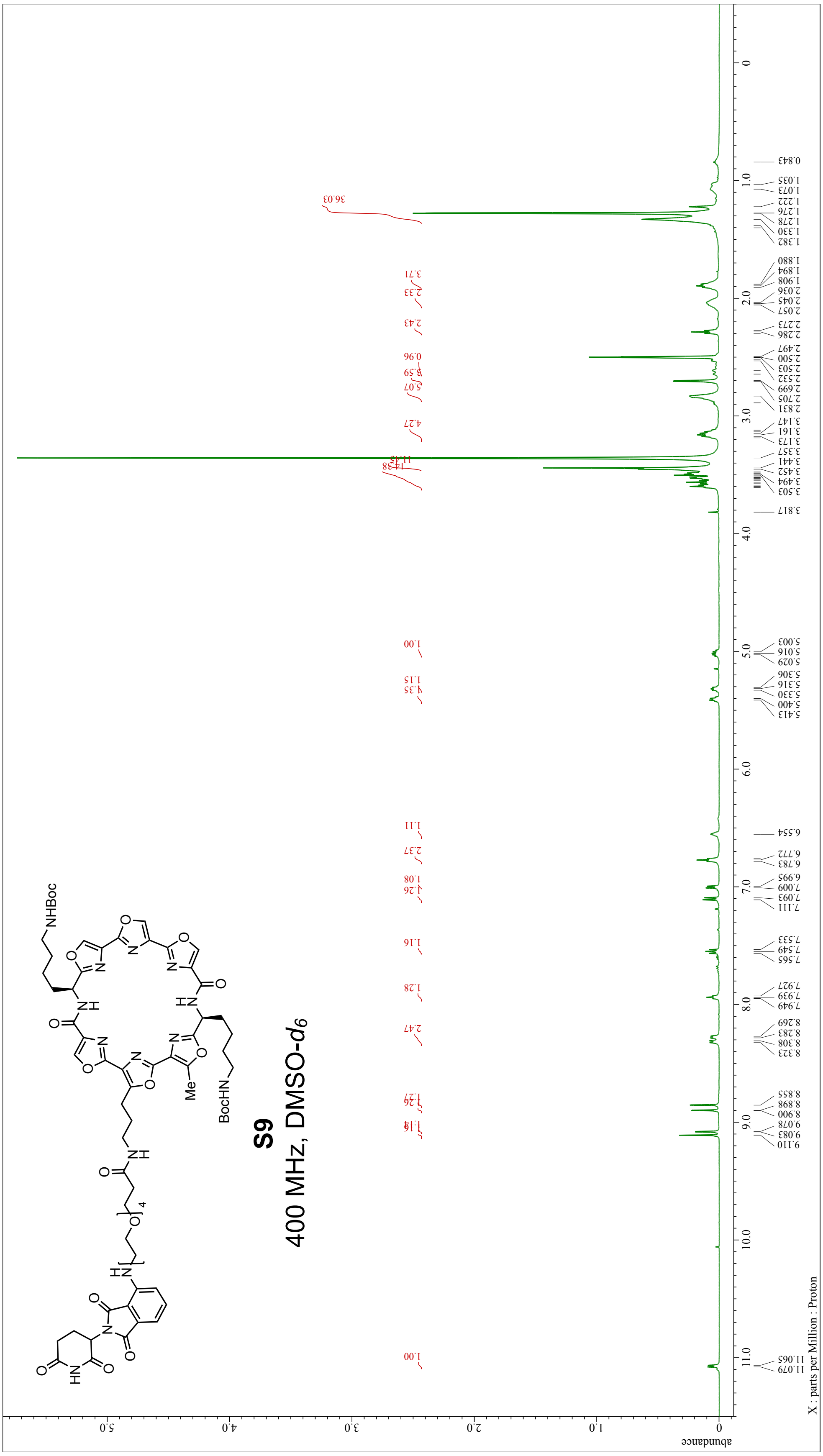
S9
400 MHz, DMSO-d_6
X : parts per Million : Proton
abundance

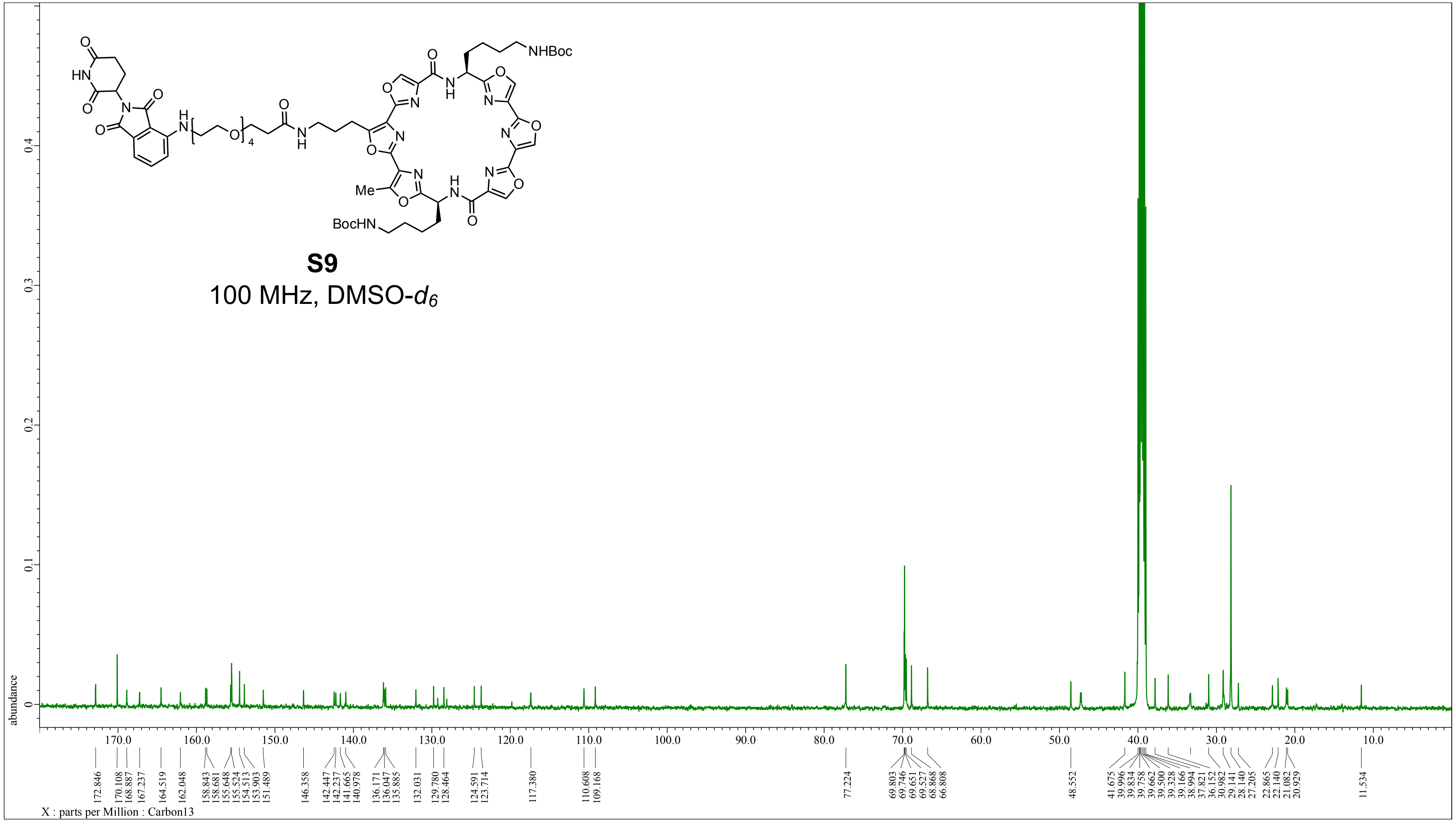

S9

100 MHz, DMSO-d_6

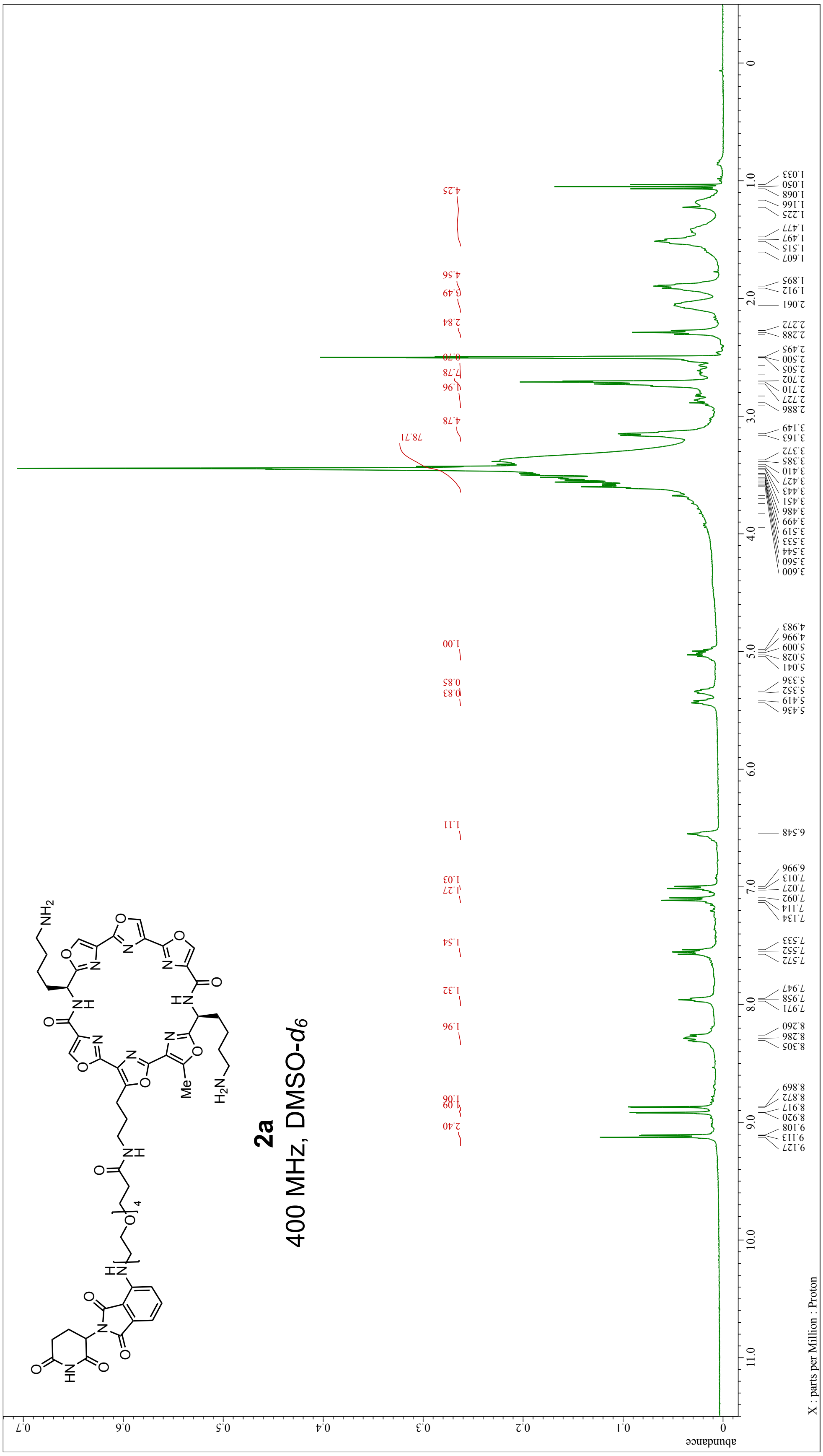
2a
400 MHz, DMSO-d_6
X : parts per Million : Proton
abundance
1.033 1.050 1.068 1.166 1.225 1.477 1.497 1.515 1.607 1.895 1.912 2.061 2.272 2.288 2.495 2.500 2.505 2.702 2.710 2.727 2.886 3.149 3.163 3.372 3.385 3.410 3.427 3.443 3.451 3.486 3.499 3.519 3.533 3.544 3.560 3.600 4.983 4.996 5.009 5.028 5.041 5.336 5.352 5.419 5.436 6.548 6.996 7.013 7.027 7.092 7.114 7.134 7.533 7.552 7.572 7.947 7.958 7.971 8.260 8.286 8.305 8.869 8.872 8.917 8.920 9.108 9.113 9.127

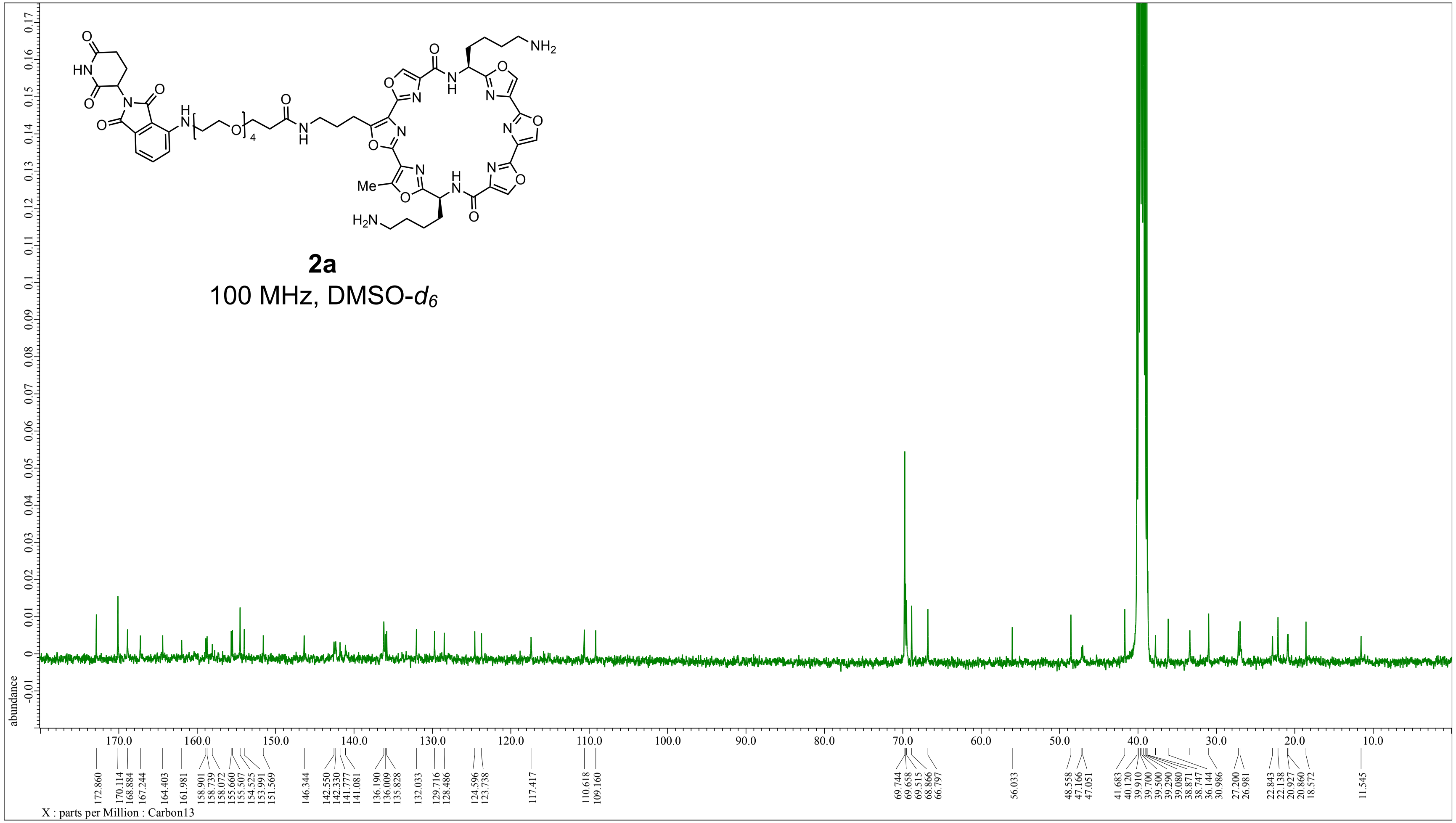
2a
100 MHz, DMSO-d_6
abundance
X : parts per Million : Carbon13
172.860 170.114 168.884 167.244 164.403 161.981 158.901 158.739 158.072 155.660 155.507 154.525 153.991 151.569 146.344 142.550 142.330 141.777 141.081 136.190 136.009 135.828 132.033 129.716 128.486 124.596 123.738 117.417 110.618 109.160 69.744 69.658 69.515 68.866 66.797 56.033 48.558 47.166 47.051 41.683 40.120 39.910 39.700 39.500 39.290 39.080 38.871 38.747 36.144 30.986 27.200 26.981 22.843 22.138 20.927 20.860 18.572 11.545

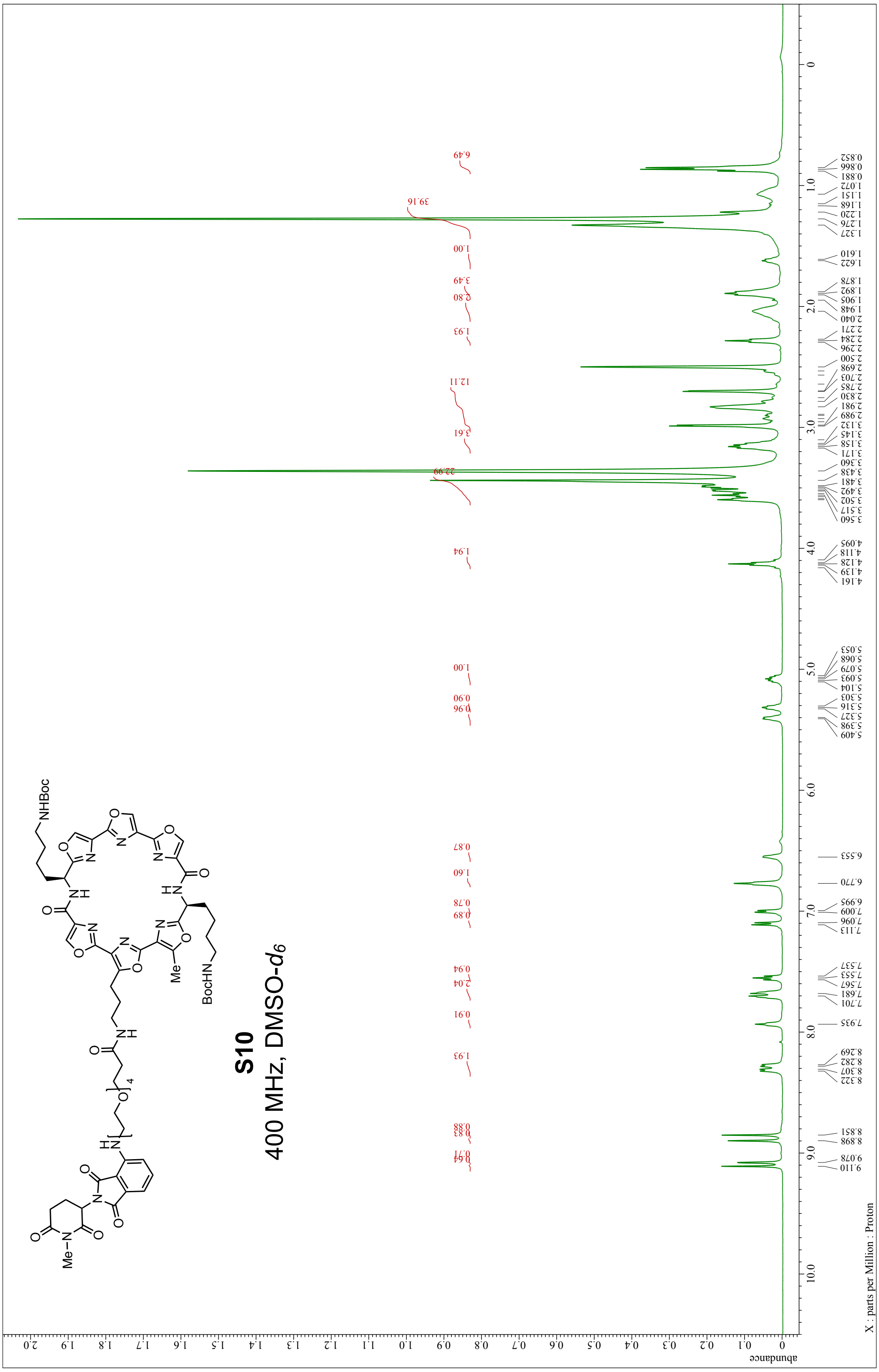
S10
400 MHz, DMSO-d_6
X : parts per Million : Proton
abundance

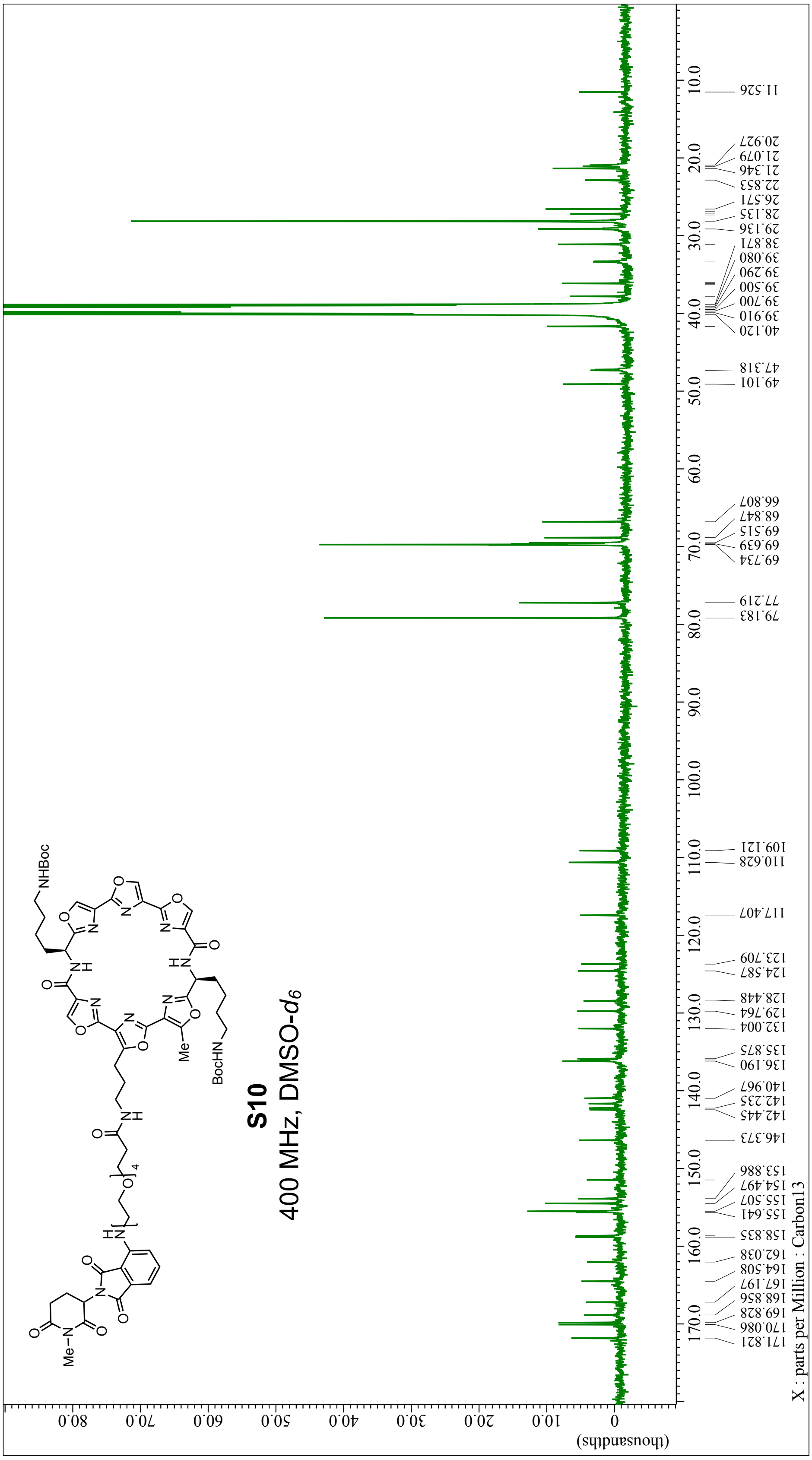

S10
400 MHz, DMSO-d6
X : parts per Million : Carbon13
(thousands)
11.526
20.927
21.079
21.346
22.853
26.571
28.135
29.136
38.871
39.080
39.290
39.500
39.700
39.910
40.120
47.318
49.101
66.807
68.847
69.515
69.639
69.734
77.219
79.183
109.121
110.628
117.407
123.709
124.587
128.448
129.764
132.004
135.875
136.190
140.967
142.235
142.445
146.373
153.886
154.497
155.507
155.641
158.835
162.038
164.508
167.197
168.856
169.828
170.086
171.821
NHBoc
BocHN
Me

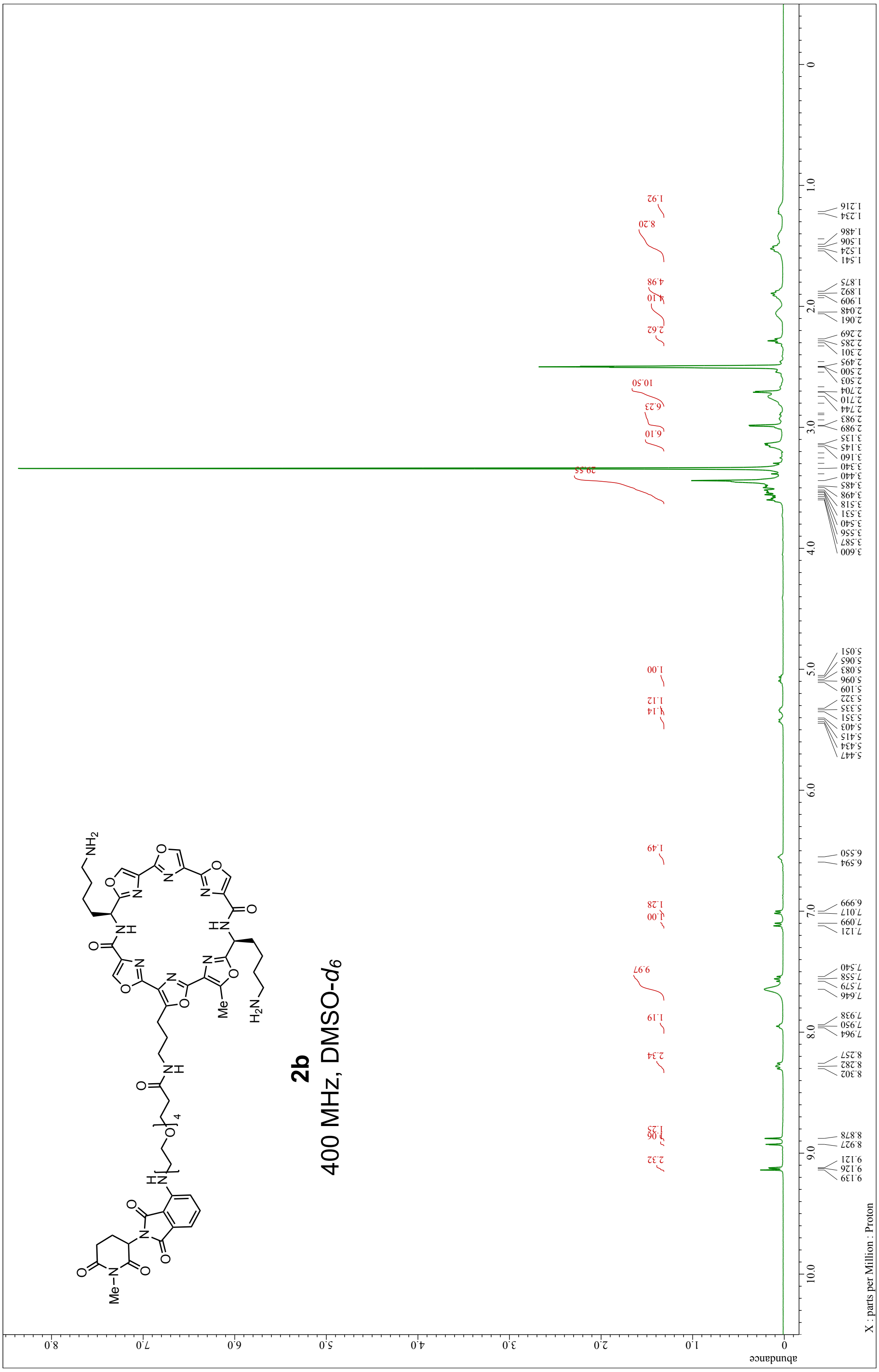
2b
400 MHz, DMSO-d_6
X : parts per Million : Proton
abundance

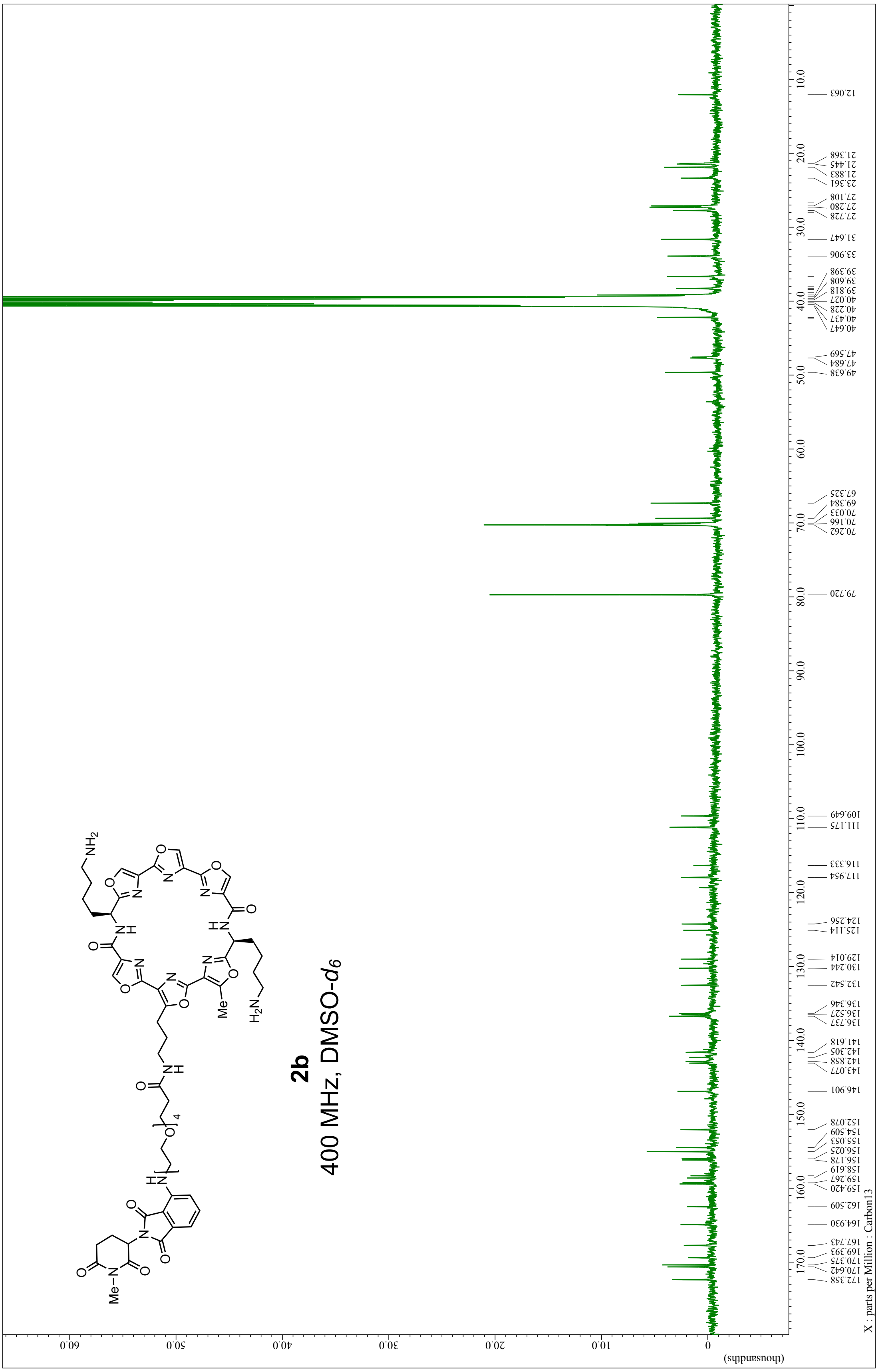
2b
400 MHz, DMSO-d_6
X : parts per Million : Carbon13
(thousandths)
12.063
21.368
21.445
21.883
23.361
27.108
27.280
27.728
31.647
33.906
39.398
39.608
39.818
40.027
40.228
40.437
40.647
47.569
47.684
49.638
67.325
69.384
70.033
70.166
70.262
79.720
109.649
111.175
116.333
117.954
124.256
125.114
129.014
130.244
132.542
136.346
136.527
136.737
141.618
142.305
142.858
143.077
146.901
152.078
154.509
155.053
156.025
156.178
158.619
159.267
159.420
162.509
164.930
167.743
169.393
170.375
170.642
172.358

Table S4. Proteins whose expression levels were reduced by more than 10% by **2a**.

Protein No.	Protein name	Master accession number	Fold change (log2 [2a/2b])
1	Complement C3	P01024	-1.021034419
2	Amyloid-beta precursor protein	P05067	-0.967099138
3	Squalene synthase	P37268	-0.926196119
4	SUN domain-containing protein 2	Q9UH99	-0.87566127
5	Heterogeneous nuclear ribonucleoprotein U-like protein 2	Q1KMD3	-0.831874991
6	Uncharacterized protein KIAA1671	Q9BY89	-0.705256017
7	Tropomodulin-3	Q9NYL9	-0.70239082
8	Phosphatidylinositol-binding clathrin assembly protein	Q13492	-0.699667004
9	E3 ubiquitin-protein ligase RNF123	Q5XPI4	-0.692820597
10	Exocyst complex component 6B	Q9Y2D4	-0.674261125
11	Inorganic pyrophosphatase 2, mitochondrial	Q9H2U2	-0.650820241
12	Basement membrane-specific heparan sulfate proteoglycan core protein	P98160	-0.641039221
13	Influenza virus NS1A-binding protein	Q9Y6Y0	-0.637146578
14	Phosphate carrier protein, mitochondrial	Q00325	-0.634133005
15	Phosphoserine aminotransferase	Q9Y617	-0.631862717
16	Hydroxymethylglutaryl-CoA synthase, cytoplasmic	Q01581	-0.621999214
17	Histone-lysine N-methyltransferase 2D	O14686	-0.621226487
18	1-phosphatidylinositol 3-phosphate 5-kinase	Q9Y2I7	-0.62018927
19	Disintegrin and metalloproteinase domain-containing protein 10	O14672	-0.614195112
20	Protein-glutamine gamma-glutamyltransferase 2	P21980	-0.613892836
21	Low-density lipoprotein receptor	P01130	-0.590913344
22	Motile sperm domain-containing protein 2	Q8NHP6	-0.581381995
23	Lanosterol 14-alpha demethylase	Q16850	-0.57864707
24	Ribosome biogenesis protein BRX1 homolog	Q8TDN6	-0.577489733
25	Asparagine synthetase [glutamine-hydrolyzing]	P08243	-0.57524426
26	Serine/threonine-protein kinase 11-interacting protein	Q8N1F8	-0.572854314
27	Peroxiredoxin-6	P30041	-0.560377431
28	Zinc finger C3H1 domain-containing protein	O60293	-0.556511722
29	Plastin-3	P13797	-0.546835421

30	Little elongation complex subunit 1	Q9Y2F5	-0.545988223
31	GTP-binding nuclear protein Ran	P62826	-0.540455196
32	Pre-rRNA-processing protein TSR1 homolog	Q2NL82	-0.530128638
33	Laminin subunit gamma-1	P11047	-0.529611501
34	Rho GTPase-activating protein 21	Q5T5U3	-0.527026139
35	Multiple PDZ domain protein	O75970	-0.526100843
36	Pleckstrin homology-like domain family B member 2	Q86SQ0	-0.524909042
37	Aspartate aminotransferase, cytoplasmic	P17174	-0.523741623
38	Glycerophosphocholine phosphodiesterase GPCPD1	Q9NPB8	-0.522159697
39	Origin recognition complex subunit 5	O43913	-0.510590156
40	Phosphorylase b kinase regulatory subunit alpha, skeletal muscle isoform	P46020	-0.509002824
41	A-kinase anchor protein 12	Q02952	-0.508687525
42	Ubiquitin carboxyl-terminal hydrolase 4	Q13107	-0.502376574
43	Prohibitin 1	P35232	-0.498726435
44	HEAT repeat-containing protein 6	Q6AI08	-0.491757197
45	Sodium bicarbonate cotransporter 3	Q9Y6M7	-0.488204158
46	Procollagen-lysine,2-oxoglutarate 5-dioxygenase 2	O00469	-0.487538937
47	Ral GTPase-activating protein subunit beta	Q86X10	-0.486533353
48	Sorbitol dehydrogenase	Q00796	-0.484000844
49	Tumor necrosis factor alpha-induced protein 3	P21580	-0.482910313
50	Cytoplasmic dynein 2 heavy chain 1	Q8NCM8	-0.477893099
51	Ornithine aminotransferase, mitochondrial	P04181	-0.476993612
52	Eukaryotic translation initiation factor 3 subunit D	O15371	-0.476632466
53	Lysophosphatidylserine lipase ABHD12	Q8N2K0	-0.476342421
54	Nuclear receptor coactivator 7	Q8NI08	-0.473790767
55	Chromodomain-helicase-DNA-binding protein 1	O14646	-0.46726547
56	RNA polymerase II-associated protein 1	Q9BWH6	-0.464400697
57	Sorting nexin-8	Q9Y5X2	-0.460370862
58	ATP-binding cassette sub-family B member 6	Q9NP58	-0.459388757
59	Bystin	Q13895	-0.457311543
60	GPI transamidase component PIG-T	Q969N2	-0.454531463

61	Zinc finger and BTB domain-containing protein 21	Q9ULJ3	-0.453489756
62	Annexin A5	P08758	-0.452365119
63	Transcription initiation factor TFIID subunit 2	Q6P1X5	-0.449410919
64	Phosphofurin acidic cluster sorting protein 1	Q6VY07	-0.448328981
65	Peroxiredoxin-1	Q06830	-0.444971988
66	Guanine nucleotide exchange factor DBS	O15068	-0.444705354
67	Phosphatidylinositol 4-phosphate 3-kinase C2 domain-containing subunit alpha	O00443	-0.441742277
68	Probable ATP-dependent RNA helicase DDX10	Q13206	-0.441024779
69	Thioredoxin domain-containing protein 5	Q8NBS9	-0.437353712
70	Calmodulin-regulated spectrin-associated protein 1	Q5T5Y3	-0.42921186
71	Protein arginine methyltransferase NDUFAF7, mitochondrial	Q7L592	-0.428382457
72	Phosphoenolpyruvate carboxykinase [GTP], mitochondrial	Q16822	-0.428139624
73	Transforming growth factor-beta receptor-associated protein 1	Q8WUH2	-0.427363286
74	2-hydroxyacyl-CoA lyase 2	A1L0T0	-0.424999803
75	ATP-citrate synthase	P53396	-0.42487149
76	39S ribosomal protein L13, mitochondrial	Q9BYD1	-0.420395543
77	Protein SGT1 homolog	Q9Y2Z0	-0.416434783
78	RNA-binding protein 14	Q96PK6	-0.415916004
79	PDZ and LIM domain protein 1	O00151	-0.415080565
80	Laminin subunit beta-1	P07942	-0.41456024
81	Nuclear pore complex protein Nup153	P49790	-0.411535374
82	4'-phosphopantetheine phosphatase	Q9NVE7	-0.411436276
83	TATA element modulatory factor	P82094	-0.406506629
84	Alpha-catulin	Q9UBT7	-0.406174235
85	Glutathione S-transferase kappa 1	Q9Y2Q3	-0.405433799
86	Leucine-rich repeat-containing protein 59	Q96AG4	-0.405400673
87	Nck-associated protein 1	Q9Y2A7	-0.403377374
88	Tensin-1	Q9HBL0	-0.402787976
89	Putative RNA polymerase II subunit B1 CTD phosphatase RPAP2	Q8IXW5	-0.401599919
90	Folylpolyglutamate synthase, mitochondrial	Q05932	-0.401318838
91	Conserved oligomeric Golgi complex subunit 1	Q8WTW3	-0.396972367

92	Fascin	Q16658	-0.392700653
93	Probable E3 ubiquitin-protein ligase HERC1	Q15751	-0.391998522
94	TAF6-like RNA polymerase II p300/CBP-associated factor-associated factor 65 kDa subunit 6L	Q9Y6J9	-0.390419269
95	LIM domain and actin-binding protein 1	Q9UHB6	-0.389093249
96	Aladin	Q9NRG9	-0.388917649
97	Nucleolar protein 6	Q9H6R4	-0.387319136
98	Nucleolar complex protein 2 homolog	Q9Y3T9	-0.385611522
99	Cleavage and polyadenylation specificity factor subunit 3	Q9UKF6	-0.381740446
100	Vacuolar protein sorting-associated protein 52 homolog	Q8N1B4	-0.381211923
101	Heterogeneous nuclear ribonucleoprotein L-like	Q8WVV9	-0.379932825
102	Contactin-associated protein 1	P78357	-0.379751008
103	U1 small nuclear ribonucleoprotein 70 kDa	P08621	-0.379694387
104	Bifunctional 3'-5' exonuclease/ATP-dependent helicase WRN	Q14191	-0.379326619
105	G-rich sequence factor 1	Q12849	-0.378097436
106	Ribonucleoside-diphosphate reductase large subunit	P23921	-0.377404895
107	Serine/threonine-protein kinase MRCK alpha	Q5VT25	-0.377268333
108	Fanconi anemia group A protein	O15360	-0.376646713
109	Spatacsin	Q96JI7	-0.376154991
110	Cip1-interacting zinc finger protein	Q9ULV3	-0.374078247
111	Prostaglandin reductase 1	Q14914	-0.373924344
112	N-acetylgalactosaminyltransferase 7	Q86SF2	-0.373583591
113	SR-related and CTD-associated factor 8	Q9UPN6	-0.372826544
114	28S ribosomal protein S29, mitochondrial	P51398	-0.372691145
115	Plasma membrane calcium-transporting ATPase 1	P20020	-0.371520706
116	E3 ubiquitin-protein ligase MIB1	Q86YT6	-0.370688916
117	Calreticulin	P27797	-0.368769974
118	Centrosomal protein of 170 kDa	Q5SW79	-0.368141518
119	Thyroid adenoma-associated protein	Q6YHU6	-0.367693982
120	Kinesin-like protein KIF1B	O60333	-0.367539589
121	Hepatocyte growth factor receptor	P08581	-0.367265784
122	Protein PALS1	Q8N3R9	-0.36682009

123	Bifunctional 3'-phosphoadenosine 5'-phosphosulfate synthase 2	O95340	-0.366365235
124	Endophilin-B2	Q9NR46	-0.365835428
125	Lanosterol synthase	P48449	-0.36580155
126	Coiled-coil and C2 domain-containing protein 1A	Q6P1N0	-0.364387962
127	Telomeric repeat-binding factor 2	Q15554	-0.364090547
128	Phosphoglucomutase-1	P36871	-0.362089748
129	Serine/threonine-protein kinase TBK1	Q9UHD2	-0.360969259
130	1-phosphatidylinositol 4,5-bisphosphate phosphodiesterase gamma-2	P16885	-0.360260847
131	DnaJ homolog subfamily C member 10	Q8IXB1	-0.359683865
132	Syntaxin-binding protein 1	P61764	-0.359487739
133	Kelch domain-containing protein 4	Q8TBB5	-0.358521125
134	Serine/threonine-protein phosphatase 2A 65 kDa regulatory subunit A alpha isoform	P30153	-0.358273134
135	Prohibitin-2	Q99623	-0.357680425
136	Transcriptional repressor NF-X1	Q12986	-0.357658483
137	Vacuolar protein sorting-associated protein 33B	Q9H267	-0.356138972
138	Pericentriolar material 1 protein	Q15154	-0.354981937
139	Eukaryotic translation initiation factor 3 subunit L	Q9Y262	-0.353622802
140	Nicotinamide phosphoribosyltransferase	P43490	-0.353402405
141	Protein CMSS1	Q9BQ75	-0.353041855
142	NADH dehydrogenase [ubiquinone] 1 alpha subcomplex subunit 10, mitochondrial	O95299	-0.351660422
143	ESF1 homolog	Q9H501	-0.348022471
144	Nucleolar MIF4G domain-containing protein 1	Q5C9Z4	-0.346994439
145	Kinase D-interacting substrate of 220 kDa	Q9ULH0	-0.346912966
146	Integrin beta-5	P18084	-0.346550394
147	Ataxin-2-like protein	Q8WWM7	-0.346547156
148	Periodic tryptophan protein 2 homolog	Q15269	-0.346076717
149	Plexin-A1	Q9UIW2	-0.343916843
150	Protein O-glucosyltransferase 2	Q6UW63	-0.343251849
151	Cdc42-interacting protein 4	Q15642	-0.342104508
152	Nucleolar complex protein 3 homolog	Q8WTT2	-0.341956421
153	Peroxisomal bifunctional enzyme	Q08426	-0.341883275

154	Progesterone-induced-blocking factor 1	Q8WXW3	-0.341801337
155	Zinc finger and BTB domain-containing protein 40	Q9NUA8	-0.341291325
156	Glycogen [starch] synthase, muscle	P13807	-0.340071123
157	Serpin H1	P50454	-0.339655751
158	Asparagine--tRNA ligase, cytoplasmic	O43776	-0.339385572
159	SPATS2-like protein	Q9NUQ6	-0.338034744
160	Long-chain fatty acid transport protein 4	Q6P1M0	-0.337572114
161	Fatty acid synthase	P49327	-0.337261158
162	Oxysterol-binding protein-related protein 10	Q9BXB5	-0.336702176
163	Bifunctional UDP-N-acetylglucosamine 2-epimerase/N-acetylmannosamine kinase	Q9Y223	-0.335815492
164	Double-stranded RNA-specific adenosine deaminase	P55265	-0.334972704
165	Protocadherin Fat 1	Q14517	-0.334552402
166	Fermitin family homolog 2	Q96AC1	-0.333630982
167	Ribosomal protein S6 kinase alpha-4	O75676	-0.332509185
168	Succinyl-CoA:3-ketoacid coenzyme A transferase 1, mitochondrial	P55809	-0.331923957
169	Kynureninase	Q16719	-0.331639216
170	Transport and Golgi organization protein 6 homolog	Q9C0B7	-0.330684697
171	Mitofusin-2	O95140	-0.330420828
172	Erbin	Q96RT1	-0.330072649
173	Nucleolar GTP-binding protein 2	Q13823	-0.329688928
174	Heterogeneous nuclear ribonucleoprotein L	P14866	-0.328233989
175	Flotillin-1	O75955	-0.327629656
176	Gamma-tubulin complex component 6	Q96RT7	-0.327097026
177	40S ribosomal protein S2	P15880	-0.327000538
178	RNA-binding protein 34	P42696	-0.326957655
179	Ubiquitin carboxyl-terminal hydrolase 24	Q9UPU5	-0.326929004
180	Alpha-ketoglutarate-dependent dioxygenase FTO	Q9C0B1	-0.326906079
181	FAST kinase domain-containing protein 2, mitochondrial	Q9NYY8	-0.326903282
182	Misshapen-like kinase 1	Q8N4C8	-0.326797996
183	GTP-binding protein 1	O00178	-0.326544232
184	Palladin	Q8WX93	-0.325885823

185	L-lactate dehydrogenase B chain	P07195	-0.324871567
186	Transcription activator BRG1	P51532	-0.324730138
187	Probable ATP-dependent RNA helicase DDX41	Q9UJV9	-0.324203282
188	Serine hydroxymethyltransferase, cytosolic	P34896	-0.323402307
189	Protein TANC2	Q9HCD6	-0.323358097
190	Proteasome activator complex subunit 4	Q14997	-0.323281703
191	Breast cancer anti-estrogen resistance protein 3	O75815	-0.323210979
192	NLR family member X1	Q86UT6	-0.323139038
193	Caspase-8	Q14790	-0.32219887
194	Zinc finger protein 644	Q9H582	-0.322126105
195	Kinesin-like protein KIF14	Q15058	-0.321764408
196	Nucleolar protein 9	Q86U38	-0.321595881
197	ATP-dependent RNA helicase DDX50	Q9BQ39	-0.320601905
198	Tyrosyl-DNA phosphodiesterase 1	Q9NUW8	-0.319863084
199	Focal adhesion kinase 1	Q05397	-0.319737761
200	BLOC-2 complex member HPS5	Q9UPZ3	-0.319450961
201	SH2 domain-containing protein 4A	Q9H788	-0.319060464
202	Uveal autoantigen with coiled-coil domains and ankyrin repeats	Q9BZF9	-0.31813184
203	Membrane-associated phosphatidylinositol transfer protein 2	Q9BZ72	-0.317753046
204	Protein enabled homolog	Q8N8S7	-0.317270168
205	Tyrosine--tRNA ligase, cytoplasmic	P54577	-0.316899978
206	MKI67 FHA domain-interacting nucleolar phosphoprotein	Q9BYG3	-0.316293664
207	Ankyrin repeat and LEM domain-containing protein 2	Q86XL3	-0.315656229
208	Structural maintenance of chromosomes flexible hinge domain-containing protein 1	A6NHR9	-0.315403972
209	Ubiquitin carboxyl-terminal hydrolase 25	Q9UHP3	-0.314621715
210	Baculoviral IAP repeat-containing protein 6	Q9NR09	-0.314566441
211	Xenotropic and polytropic retrovirus receptor 1	Q9UBH6	-0.314250735
212	Propionyl-CoA carboxylase beta chain, mitochondrial	P05166	-0.313609172
213	Zinc finger protein 106	Q9H2Y7	-0.312966638
214	Splicing factor, suppressor of white-apricot homolog	Q12872	-0.312764838
215	Aspartate aminotransferase, mitochondrial	P00505	-0.31273403

216	NADPH--cytochrome P450 reductase	P16435	-0.311925126
217	General transcription and DNA repair factor IIH helicase subunit XPD	P18074	-0.311351535
218	Integrin beta-1	P05556	-0.311265657
219	Nucleolar complex protein 4 homolog	Q9BVI4	-0.311208858
220	Putative ATP-dependent RNA helicase DHX57	Q6P158	-0.311178872
221	Rho guanine nucleotide exchange factor 11	O15085	-0.310957753
222	Leukemia inhibitory factor receptor	P42702	-0.309069953
223	Protein kinase C and casein kinase substrate in neurons protein 3	Q9UKS6	-0.309051824
224	General transcription factor 3C polypeptide 5	Q9Y5Q8	-0.308931054
225	Histone deacetylase 3	O15379	-0.308099551
226	Golgin subfamily A member 2	Q08379	-0.307858969
227	Prolyl 4-hydroxylase subunit alpha-2	O15460	-0.30705633
228	Tubulin--tyrosine ligase-like protein 12	Q14166	-0.306478554
229	Parafibromin	Q6P1J9	-0.306311557
230	EH domain-binding protein 1	Q8NDI1	-0.305848821
231	GDP-mannose 4,6 dehydratase	O60547	-0.305132437
232	Ras-related protein Rab-7a	P51149	-0.305038611
233	Carboxypeptidase D	O75976	-0.304872769
234	Exportin-1	O14980	-0.304723834
235	Calmodulin-regulated spectrin-associated protein 2	Q08AD1	-0.303932388
236	Bromodomain and WD repeat-containing protein 1	Q9NSI6	-0.303410061
237	Kinesin-like protein KIF15	Q9NS87	-0.303395221
238	Ras GTPase-activating-like protein IQGAP3	Q86VI3	-0.303367666
239	Integrin alpha-1	P56199	-0.30257962
240	26S proteasome non-ATPase regulatory subunit 12	O00232	-0.302569776
241	Mediator of RNA polymerase II transcription subunit 23	Q9ULK4	-0.302556444
242	Keratin, type II cytoskeletal 2 epidermal	P35908	-0.30243695
243	ATP-binding cassette sub-family D member 3	P28288	-0.302323561
244	ATP-dependent DNA helicase Q4	O94761	-0.300860763
245	Double-stranded RNA-specific editase B2	Q9NS39	-0.300518808
246	Sorting nexin-5	Q9Y5X3	-0.299356841

247	Protein flightless-1 homolog	Q13045	-0.298418961
248	Intersectin-2	Q9NZM3	-0.298069681
249	Glutamine-dependent NAD(+) synthetase	Q6IA69	-0.297628832
250	ATP-dependent RNA helicase DDX54	Q8TDD1	-0.297065281
251	Protein O-GlcNAcase	O60502	-0.296928506
252	Acyl-coenzyme A thioesterase 9, mitochondrial	Q9Y305	-0.296288722
253	Hydroxysteroid dehydrogenase-like protein 2	Q6YN16	-0.295302676
254	Calpain-2 catalytic subunit	P17655	-0.295252511
255	Dedicator of cytokinesis protein 6	Q96HP0	-0.294218575
256	Protein CIP2A	Q8TCG1	-0.294028332
257	PCI domain-containing protein 2	Q5JVF3	-0.293749025
258	ATP-dependent RNA helicase DDX55	Q8NHQ9	-0.293330831
259	NHL repeat-containing protein 2	Q8NBF2	-0.293048702
260	28S ribosomal protein S22, mitochondrial	P82650	-0.292930262
261	Thioredoxin reductase 1, cytoplasmic	Q16881	-0.292778664
262	Threonine--tRNA ligase 1, cytoplasmic	P26639	-0.29119399
263	Erythroid differentiation-related factor 1	Q3B7T1	-0.29074505
264	Intersectin-1	Q15811	-0.290463399
265	Protein arginine N-methyltransferase 5	O14744	-0.290384503
266	eIF5-mimic protein 1	Q9Y6E2	-0.290001203
267	Procollagen galactosyltransferase 1	Q8NBJ5	-0.289928366
268	Rapamycin-insensitive companion of mTOR	Q6R327	-0.28665949
269	Heterogeneous nuclear ribonucleoprotein U	Q00839	-0.286658683
270	Bifunctional purine biosynthesis protein ATIC	P31939	-0.285631706
271	DNA-directed RNA polymerase III subunit RPC5	Q9NVU0	-0.284598462
272	Ubiquitin carboxyl-terminal hydrolase 47	Q96K76	-0.283731443
273	Fatty acid CoA ligase Acsl3	O95573	-0.2835489
274	U3 small nucleolar RNA-associated protein 15 homolog	Q8TED0	-0.28244875
275	Probable ATP-dependent RNA helicase DDX47	Q9H0S4	-0.281972606
276	E3 ubiquitin-protein ligase TRIP12	Q14669	-0.281731691
277	Condensin complex subunit 2	Q15003	-0.281588427
278	tRNA-dihydrouridine(47) synthase [NAD(P)(+)]-like	Q96G46	-0.280274253

279	Cell division cycle protein 27 homolog	P30260	-0.278981093
280	Protein Aster-B	Q3KR37	-0.278947336
281	Keratin, type II cytoskeletal 7	P08729	-0.277983509
282	Protein transport protein Sec24D	O94855	-0.277479425
283	Pseudouridylate synthase TRUB2, mitochondrial	O95900	-0.277164476
284	Rab3 GTPase-activating protein non-catalytic subunit	Q9H2M9	-0.276706836
285	Fructose-bisphosphate aldolase C	P09972	-0.276228597
286	Chloride intracellular channel protein 4	Q9Y696	-0.275358904
287	Histone-lysine N-methyltransferase 2B	Q9UMN6	-0.274967361
288	Fibronectin type-III domain-containing protein 3A	Q9Y2H6	-0.27483442
289	Paired amphipathic helix protein Sin3b	O75182	-0.273846816
290	Mediator of RNA polymerase II transcription subunit 14	O60244	-0.273676593
291	Rac GTPase-activating protein 1	Q9H0H5	-0.273312674
292	Intermembrane lipid transfer protein VPS13C	Q709C8	-0.272799983
293	Threonine synthase-like 1	Q8IYQ7	-0.271657708
294	SRSF protein kinase 1	Q96SB4	-0.271194378
295	Myotubularin-related protein 13	Q86WG5	-0.271069236
296	Kelch-like protein 7	Q8IXQ5	-0.269790692
297	Ubiquitin carboxyl-terminal hydrolase 14	P54578	-0.268966632
298	Signal recognition particle subunit SRP72	O76094	-0.268359229
299	Calpain-1 catalytic subunit	P07384	-0.268169414
300	Calpain-7	Q9Y6W3	-0.267107493
301	E3 ubiquitin-protein ligase ARIH2	O95376	-0.266111753
302	Metastasis-associated protein MTA2	O94776	-0.264632434
303	NADH-ubiquinone oxidoreductase 75 kDa subunit, mitochondrial	P28331	-0.264620711
304	Probable RNA-binding protein 19	Q9Y4C8	-0.26461345
305	Replication factor C subunit 2	P35250	-0.263819342
306	Prosaposin	P07602	-0.262866059
307	DDB1- and CUL4-associated factor 13	Q9NV06	-0.262324063
308	Disintegrin and metalloproteinase domain-containing protein 9	Q13443	-0.261821447
309	Treacle protein	Q13428	-0.261799516
310	Apoptosis inhibitor 5	Q9BZZ5	-0.261714311

311	Coronin-1C	Q9ULV4	-0.261392503
312	Cysteine--tRNA ligase, cytoplasmic	P49589	-0.261166262
313	Inositol polyphosphate 5-phosphatase OCRL	Q01968	-0.260915428
314	YEATS domain-containing protein 2	Q9ULM3	-0.260750038
315	Probable JmjC domain-containing histone demethylation protein 2C	Q15652	-0.260541493
316	Plakophilin-3	Q9Y446	-0.260372282
317	Keratin, type I cytoskeletal 9	P35527	-0.260201093
318	Molybdenum cofactor sulfurase	Q96EN8	-0.259610764
319	Phosphatidylinositol 5-phosphate 4-kinase type-2 gamma	Q8TBX8	-0.259276761
320	AMP deaminase 2	Q01433	-0.259063937
321	Triple functional domain protein	O75962	-0.258900406
322	Histone-lysine N-methyltransferase SETD1A	O15047	-0.258810955
323	Polyadenylate-binding protein 4	Q13310	-0.258514095
324	Histone-arginine methyltransferase CARM1	Q86X55	-0.257932916
325	Ubiquitin carboxyl-terminal hydrolase 10	Q14694	-0.257476097
326	Rho family-interacting cell polarization regulator 1	Q6ZS17	-0.257287306
327	Trafficking protein particle complex subunit 11	Q7Z392	-0.257233606
328	Heterochromatin protein 1-binding protein 3	Q5SSJ5	-0.256844342
329	Bifunctional 3'-phosphoadenosine 5'-phosphosulfate synthase 1	O43252	-0.256771155
330	Mitofusin-1	Q8IWA4	-0.256725148
331	NADH dehydrogenase [ubiquinone] 1 alpha subcomplex subunit 9, mitochondrial	Q16795	-0.256307465
332	26S proteasome regulatory subunit 8	P62195	-0.255966601
333	RUN and FYVE domain-containing protein 1	Q96T51	-0.254343266
334	X-ray repair cross-complementing protein 6	P12956	-0.253406307
335	26S proteasome non-ATPase regulatory subunit 5	Q16401	-0.253339332
336	HMG box transcription factor BBX	Q8WY36	-0.25287494
337	Guanine nucleotide exchange factor VAV2	P52735	-0.251307382
338	Charged multivesicular body protein 7	Q8WUX9	-0.250812009
339	FACT complex subunit SSRP1	Q08945	-0.250729859
340	Heterogeneous nuclear ribonucleoprotein A3	P51991	-0.249576117
341	Ubiquitin carboxyl-terminal hydrolase 3	Q9Y6I4	-0.249285405

342	Importin subunit alpha-1	P52292	-0.248326359
343	Nucleoporin p54	Q7Z3B4	-0.24768709
344	Testin	Q9UGI8	-0.247501628
345	Lysine-rich nucleolar protein 1	Q1ED39	-0.24748416
346	Ran-binding protein 6	O60518	-0.247064962
347	Nucleolar protein 10	Q9BSC4	-0.24685596
348	Interferon-induced 35 kDa protein	P80217	-0.246344514
349	Aldehyde dehydrogenase family 16 member A1	Q8IZ83	-0.246295438
350	Nuclear migration protein nudC	Q9Y266	-0.245899515
351	La-related protein 4B	Q92615	-0.245864923
352	Methionine--tRNA ligase, cytoplasmic	P56192	-0.245150007
353	Endothelin-converting enzyme 1	P42892	-0.244671199
354	GTP-binding protein 4	Q9BZE4	-0.24420572
355	Exocyst complex component 1	Q9NV70	-0.243606378
356	Exocyst complex component 5	O00471	-0.242908032
357	Ubiquitin carboxyl-terminal hydrolase 15	Q9Y4E8	-0.242620012
358	Eukaryotic translation initiation factor 2A	Q9BY44	-0.242211953
359	TBC1 domain family member 1	Q86TI0	-0.242121362
360	Phosphatidylinositol 4,5-bisphosphate 3-kinase catalytic subunit alpha isoform	P42336	-0.241933586
361	60S ribosomal protein L7a	P62424	-0.241637991
362	Heat shock protein HSP 90-beta	P08238	-0.241524183
363	Helicase-like transcription factor	Q14527	-0.240905301
364	Protein TANC1	Q9C0D5	-0.238816932
365	Nuclear receptor corepressor 1	O75376	-0.238511524
366	Protoporphyrinogen oxidase	P50336	-0.238029344
367	Probable ATP-dependent RNA helicase DHX40	Q8IX18	-0.237745614
368	Peptidyl-prolyl cis-trans isomerase FKBP5	Q13451	-0.236762294
369	CAP-Gly domain-containing linker protein 1	P30622	-0.236567368
370	Ras-related protein Rab-20	Q9NX57	-0.236139291
371	Dystonin	Q03001	-0.236005922
372	E3 ubiquitin-protein ligase UHRF1	Q96T88	-0.235926824

373	Structural maintenance of chromosomes protein 5	Q8IY18	-0.235813126
374	26S proteasome non-ATPase regulatory subunit 6	Q15008	-0.235464355
375	Exportin-T	O43592	-0.235047726
376	Protein furry homolog-like	O94915	-0.234581198
377	Clathrin heavy chain 1	Q00610	-0.234476347
378	Vacuolar protein-sorting-associated protein 36	Q86VN1	-0.234203417
379	Histone-lysine N-methyltransferase, H3 lysine-36 specific	Q96L73	-0.233028168
380	Nuclear pore membrane glycoprotein 210	Q8TEM1	-0.232873785
381	ATPase MORC2	Q9Y6X9	-0.232851472
382	Thyroid receptor-interacting protein 6	Q15654	-0.232775137
383	Germinal-center associated nuclear protein	O60318	-0.232605144
384	Tyrosine-protein phosphatase non-receptor type 12	Q05209	-0.232497626
385	Protein disulfide-isomerase	P07237	-0.232321061
386	DnaJ homolog subfamily C member 16	Q9Y2G8	-0.232154164
387	Gamma-soluble NSF attachment protein	Q99747	-0.231627242
388	Eukaryotic translation initiation factor 5	P55010	-0.231519584
389	L-lactate dehydrogenase A chain	P00338	-0.231454292
390	Cullin-3	Q13618	-0.231143669
391	Peptidyl-prolyl cis-trans isomerase A	P62937	-0.230782273
392	Protein zyg-11 homolog B	Q9C0D3	-0.230600275
393	LIM and calponin homology domains-containing protein 1	Q9UPQ0	-0.229918706
394	Protein RCC2	Q9P258	-0.229877354
395	Dual specificity protein phosphatase 12	Q9UNI6	-0.229874092
396	Extended synaptotagmin-1	Q9BSJ8	-0.229846851
397	Monofunctional C1-tetrahydrofolate synthase, mitochondrial	Q6UB35	-0.229642105
398	Glutamine--fructose-6-phosphate aminotransferase [isomerizing] 1	Q06210	-0.228684904
399	Ankyrin repeat and KH domain-containing protein 1	Q8IWZ3	-0.228570214
400	Ataxin-2	Q99700	-0.228090348
401	Importin-11	Q9UI26	-0.22755425
402	Nuclear pore complex protein Nup205	Q92621	-0.227053212
403	Ectopic P granules protein 5 homolog	Q9HCE0	-0.22673638

404	N-alpha-acetyltransferase 50	Q9GZZ1	-0.226401794
405	Phosphoinositide 3-kinase regulatory subunit 4	Q99570	-0.226353243
406	Keratin, type II cytoskeletal 1	P04264	-0.225992907
407	26S proteasome non-ATPase regulatory subunit 2	Q13200	-0.225421988
408	Collagen alpha-1(XII) chain	Q99715	-0.225272196
409	RNA helicase aquarius	O60306	-0.225130767
410	Sushi, von Willebrand factor type A, EGF and pentraxin domain-containing protein 1	Q4LDE5	-0.224923808
411	AH receptor-interacting protein	O00170	-0.224714719
412	HEAT repeat-containing protein 5A	Q86XA9	-0.224439521
413	Microtubule-actin cross-linking factor 1, isoforms 1/2/3/4/5	Q9UPN3	-0.224303426
414	RNA polymerase II-associated factor 1 homolog	Q8N7H5	-0.223452949
415	Ribosome biogenesis protein BMS1 homolog	Q14692	-0.222977295
416	Pseudouridylate synthase 7 homolog	Q96PZ0	-0.222758179
417	Sorting nexin-27	Q96L92	-0.222182947
418	DNA polymerase delta subunit 2	P49005	-0.221726421
419	Cytosol aminopeptidase	P28838	-0.221713929
420	Bifunctional phosphoribosylaminoimidazole carboxylase/phosphoribosylaminoimidazole succinocarboxamide synthetase	P22234	-0.221537187
421	Cytochrome b-c1 complex subunit 1, mitochondrial	P31930	-0.22101946
422	Purine nucleoside phosphorylase	P00491	-0.220953163
423	SUN domain-containing protein 1	O94901	-0.220861174
424	Intermembrane lipid transfer protein VPS13A	Q96RL7	-0.220583927
425	Protein Wiz	O95785	-0.220577013
426	E3 ubiquitin-protein ligase UBR4	Q5T4S7	-0.220478415
427	Laminin subunit alpha-5	O15230	-0.220240948
428	Adenylosuccinate lyase	P30566	-0.219668836
429	E3 ubiquitin-protein ligase HERC2	O95714	-0.219394988
430	Pseudouridylate synthase RPUSD2	Q8IZ73	-0.217968681
431	Zyxin	Q15942	-0.217862584
432	Glycine--tRNA ligase	P41250	-0.217707069
433	Transducin beta-like protein 3	Q12788	-0.217619533
434	Elongation factor G, mitochondrial	Q96RP9	-0.217566518

435	Tetratricopeptide repeat protein 28	Q96AY4	-0.217397234
436	DNA excision repair protein ERCC-6-like	Q2NKX8	-0.216473775
437	THO complex subunit 6 homolog	Q86W42	-0.216363213
438	Actin filament-associated protein 1	Q8N556	-0.21624986
439	PH-interacting protein	Q8WWQ0	-0.215753475
440	Histone-lysine N-methyltransferase SETDB1	Q15047	-0.215732296
441	5'-3' exoribonuclease 2	Q9H0D6	-0.215544547
442	Protein FRG1	Q14331	-0.215334738
443	Pre-mRNA-processing factor 40 homolog A	O75400	-0.215276951
444	ATP-dependent DNA/RNA helicase DHX36	Q9H2U1	-0.21503114
445	Cysteine and histidine-rich domain-containing protein 1	Q9UHD1	-0.21451749
446	Ubiquitin-like modifier-activating enzyme 6	A0AVT1	-0.214385205
447	Nucleoporin NDC1	Q9BTX1	-0.214225183
448	N6-adenosine-methyltransferase catalytic subunit	Q86U44	-0.213963562
449	Golgi-associated PDZ and coiled-coil motif-containing protein	Q9HD26	-0.212524516
450	Alpha-actinin-1	P12814	-0.212514664
451	Ribosomal RNA small subunit methyltransferase NEP1	Q92979	-0.212342529
452	Endoplasmic reticulum resident protein 44	Q9BS26	-0.211874146
453	Golgi integral membrane protein 4	O00461	-0.211131467
454	Zinc finger and BTB domain-containing protein 11	O95625	-0.211064706
455	Ribonucleoprotein PTB-binding 1	Q8IY67	-0.210584273
456	Sorting and assembly machinery component 50 homolog	Q9Y512	-0.210466742
457	RNA-binding protein 28	Q9NW13	-0.209566459
458	Protein ITPRID2	P28290	-0.209332165
459	Alpha-N-acetylglucosaminidase	P54802	-0.209327601
460	Oxysterol-binding protein-related protein 1	Q9BXW6	-0.209201119
461	26S proteasome non-ATPase regulatory subunit 3	O43242	-0.20909185
462	Nesprin-2	Q8WXH0	-0.209059102
463	Genetic suppressor element 1	Q14687	-0.20898898
464	NADH-cytochrome b5 reductase 1	Q9UHQ9	-0.208686072
465	Dolichyl-diphosphooligosaccharide--protein glycosyltransferase subunit STT3A	P46977	-0.208534829

466	Nuclear pore complex protein Nup160	Q12769	-0.208340186
467	Elongator complex protein 2	Q6IA86	-0.208245046
468	Unconventional myosin-Ib	O43795	-0.207680038
469	Gamma-tubulin complex component 3	Q96CW5	-0.207574442
470	WASH complex subunit 4	Q2M389	-0.206739918
471	NBAS subunit of NRZ tethering complex	A2RRP1	-0.206596755
472	AT-rich interactive domain-containing protein 1B	Q8NFD5	-0.206584533
473	Leucine-rich repeat-containing protein 47	Q8N1G4	-0.206073945
474	Torsin-1A-interacting protein 2	Q8NFQ8	-0.203705608
475	Lupus La protein	P05455	-0.203398667
476	Insulin-like growth factor 2 mRNA-binding protein 3	O00425	-0.203284322
477	SKI2 subunit of superkiller complex protein	Q15477	-0.20315379
478	Paxillin	P49023	-0.203106834
479	Acetyl-CoA carboxylase 1	Q13085	-0.203081245
480	CAD protein	P27708	-0.202902603
481	CCR4-NOT transcription complex subunit 9	Q92600	-0.202823329
482	Staphylococcal nuclease domain-containing protein 1	Q7KZF4	-0.202780587
483	Anoctamin-6	Q4KMQ2	-0.202637624
484	Centrosomal protein of 97 kDa	Q8IW35	-0.202460838
485	Metal transporter CNNM3	Q8NE01	-0.202016864
486	Mitochondrial intermediate peptidase	Q99797	-0.201773713
487	Dipeptidyl peptidase 3	Q9NY33	-0.201671009
488	Nuclear pore complex protein Nup133	Q8WUM0	-0.201577301
489	Protein TASOR	Q9UK61	-0.201507605
490	Prolyl endopeptidase	P48147	-0.201471872
491	Ankyrin repeat and SAM domain-containing protein 1A	Q92625	-0.201427317
492	Brain-specific angiogenesis inhibitor 1-associated protein 2	Q9UQB8	-0.20125738
493	Dipeptidyl peptidase 9	Q86TI2	-0.200818459
494	Eukaryotic translation initiation factor 2 subunit 2	P20042	-0.200813579
495	Centrosomal protein of 290 kDa	O15078	-0.200565271
496	FAST kinase domain-containing protein 5, mitochondrial	Q7L8L6	-0.200284989
497	Protein phosphatase 1 regulatory subunit 21	Q6ZMI0	-0.200283167

498	Serine/threonine-protein phosphatase 6 regulatory subunit 1	Q9UPN7	-0.200176296
499	Protein mono-ADP-ribosyltransferase PARP4	Q9UKK3	-0.200042218
500	Vacuolar protein sorting-associated protein 16 homolog	Q9H269	-0.199512487
501	T-complex protein 1 subunit gamma	P49368	-0.199423432
502	SLIT-ROBO Rho GTPase-activating protein 1	Q7Z6B7	-0.198733342
503	Methionine adenosyltransferase 2 subunit beta	Q9NZL9	-0.198693504
504	Sister chromatid cohesion protein PDS5 homolog B	Q9NTI5	-0.197358355
505	Filamin-A	P21333	-0.196795017
506	Ubiquitin conjugation factor E4 A	Q14139	-0.196362137
507	Golgi apparatus protein 1	Q92896	-0.196052383
508	Inhibitor of nuclear factor kappa-B kinase subunit beta	O14920	-0.195987069
509	Leukocyte receptor cluster member 8	Q96PV6	-0.195930757
510	Vimentin	P08670	-0.195902775
511	Phosphatidylinositol 3-kinase catalytic subunit type 3	Q8NEB9	-0.195711528
512	Eukaryotic translation initiation factor 3 subunit I	Q13347	-0.19509877
513	ATP-dependent 6-phosphofructokinase, liver type	P17858	-0.194787995
514	Sickle tail protein homolog	Q5T5P2	-0.194619852
515	Presequence protease, mitochondrial	Q5JRX3	-0.19439024
516	Radixin	P35241	-0.194162903
517	Transcriptional regulator ATRX	P46100	-0.194157773
518	MAU2 chromatid cohesion factor homolog	Q9Y6X3	-0.193678537
519	Activating signal cointegrator 1 complex subunit 3	Q8N3C0	-0.192437402
520	RNA-binding protein FXR1	P51114	-0.192364642
521	General transcription and DNA repair factor IIH helicase subunit XPB	P19447	-0.192111999
522	UDP-N-acetylglucosamine--peptide N-acetylglucosaminyltransferase 110 kDa subunit	O15294	-0.192024977
523	Mitotic spindle assembly checkpoint protein MAD1	Q9Y6D9	-0.191836348
524	Tubulin-specific chaperone D	Q9BTW9	-0.191815516
525	E3 ubiquitin-protein ligase TRIM33	Q9UPN9	-0.191680242
526	E3 ubiquitin-protein ligase UBR3	Q6ZT12	-0.190935354
527	Neurobeachin-like protein 2	Q6ZNJ1	-0.190489817
528	Dihydroxyacetone phosphate acyltransferase	O15228	-0.190130558

529	Oxygen-dependent coproporphyrinogen-III oxidase, mitochondrial	P36551	-0.19011263
530	Bifunctional glutamate/proline--tRNA ligase	P07814	-0.189882187
531	Eukaryotic initiation factor 4A-I	P60842	-0.189842679
532	HAUS augmin-like complex subunit 3	Q68CZ6	-0.18893557
533	Endoplasmic reticulum chaperone BiP	P11021	-0.188894938
534	3-hydroxyacyl-CoA dehydrogenase type-2	Q99714	-0.188653268
535	Stress-induced-phosphoprotein 1	P31948	-0.188541206
536	Tyrosine-protein phosphatase non-receptor type 9	P43378	-0.18850115
537	Protein phosphatase 1 regulatory subunit 12A	O14974	-0.188377256
538	6-phosphogluconate dehydrogenase, decarboxylating	P52209	-0.188242393
539	Rabankyrin-5	Q9P2R3	-0.18730834
540	116 kDa U5 small nuclear ribonucleoprotein component	Q15029	-0.18707674
541	Protein transport protein Sec23A	Q15436	-0.186948605
542	Long-chain-fatty-acid--CoA ligase 4	O60488	-0.186945108
543	Mediator of RNA polymerase II transcription subunit 24	O75448	-0.186927037
544	Cytoplasmic dynein 1 light intermediate chain 1	Q9Y6G9	-0.186449919
545	ATP-dependent 6-phosphofructokinase, platelet type	Q01813	-0.185822525
546	TBC1 domain family member 9B	Q66K14	-0.185716825
547	Isocitrate dehydrogenase [NADP] cytoplasmic	O75874	-0.18536554
548	Ras-associated and pleckstrin homology domains-containing protein 1	Q70E73	-0.185092567
549	Atlastin-3	Q6DD88	-0.184707973
550	Probable ATP-dependent RNA helicase DDX31	Q9H8H2	-0.18457545
551	Probable ATP-dependent RNA helicase DDX52	Q9Y2R4	-0.184497729
552	Coatomer subunit beta'	P35606	-0.183742213
553	Vigilin	Q00341	-0.183628752
554	Polycomb protein EED	O75530	-0.183592275
555	2-oxoglutarate dehydrogenase complex component E1	Q02218	-0.183512042
556	Terminal uridylyltransferase 7	Q5VYS8	-0.183396367
557	Protein transport protein Sec24A	O95486	-0.182733383
558	2',3'-cyclic-nucleotide 3'-phosphodiesterase	P09543	-0.1826679
559	Nonsense-mediated mRNA decay factor SMG7	Q92540	-0.182551374

560	GTPase Era, mitochondrial	O75616	-0.182425311
561	Sperm-associated antigen 1	Q07617	-0.181968267
562	Anaphase-promoting complex subunit 2	Q9UJX6	-0.181829543
563	Exocyst complex component 2	Q96KP1	-0.181780895
564	Leucine-rich repeat-containing protein 40	Q9H9A6	-0.181480633
565	HAUS augmin-like complex subunit 6	Q7Z4H7	-0.181003551
566	Dolichyl-diphosphooligosaccharide--protein glycosyltransferase subunit 1	P04843	-0.180810211
567	Alpha-mannosidase 2	Q16706	-0.180636199
568	Vacuolar protein sorting-associated protein 11 homolog	Q9H270	-0.180380629
569	Protein FAM91A1	Q658Y4	-0.180230751
570	Tyrosine-protein phosphatase non-receptor type 11	Q06124	-0.180022245
571	Leucine--tRNA ligase, mitochondrial	Q15031	-0.179839812
572	ADP-ribosylation factor GTPase-activating protein 2	Q8N6H7	-0.179428548
573	DNA mismatch repair protein Mlh1	P40692	-0.179125997
574	Leucine--tRNA ligase, cytoplasmic	Q9P2J5	-0.178876404
575	Heat shock protein 105 kDa	Q92598	-0.178856978
576	Agrin	O00468	-0.178678533
577	Septin-7	Q16181	-0.178197723
578	Syntaxin-binding protein 3	O00186	-0.178125034
579	Disco-interacting protein 2 homolog B	Q9P265	-0.178091587
580	Succinate dehydrogenase [ubiquinone] flavoprotein subunit, mitochondrial	P31040	-0.177406226
581	Annexin A3	P12429	-0.17723642
582	Coatomer subunit gamma-2	Q9UBF2	-0.177150038
583	Nonsense-mediated mRNA decay factor SMG8	Q8ND04	-0.176817857
584	Serine/threonine-protein kinase SIK2	Q9H0K1	-0.17654813
585	Deoxyhypusine synthase	P49366	-0.17634015
586	Eukaryotic translation initiation factor 4 gamma 3	O43432	-0.175832719
587	U4/U6.U5 tri-snRNP-associated protein 1	O43290	-0.175246101
588	FAST kinase domain-containing protein 4	Q969Z0	-0.174747846
589	Pumilio homolog 3	Q15397	-0.174581805
590	Nuclear factor NF-kappa-B p100 subunit	Q00653	-0.174322304

591	Lysine-specific histone demethylase 1A	O60341	-0.174308992
592	Vacuolar fusion protein CCZ1 homolog B	P86790	-0.174265112
593	Clathrin interactor 1	Q14677	-0.173876029
594	AP-2 complex subunit beta	P63010	-0.17366794
595	E3 ubiquitin-protein ligase HECTD1	Q9ULT8	-0.173343728
596	Conserved oligomeric Golgi complex subunit 6	Q9Y2V7	-0.173317198
597	Thymidylate kinase	P23919	-0.173301251
598	FAS-associated factor 1	Q9UNN5	-0.172974473
599	Conserved oligomeric Golgi complex subunit 4	Q9H9E3	-0.172919482
600	Protein SON	P18583	-0.172016702
601	Mitotic interactor and substrate of PLK1	Q8IVT2	-0.171936966
602	Isoleucine--tRNA ligase, mitochondrial	Q9NSE4	-0.171739968
603	Hypoxia up-regulated protein 1	Q9Y4L1	-0.170814162
604	TBC1 domain family member 15	Q8TC07	-0.170807289
605	Lymphoid-specific helicase	Q9NRZ9	-0.169613706
606	Protein LTV1 homolog	Q96GA3	-0.169500941
607	4-trimethylaminobutyraldehyde dehydrogenase	P49189	-0.169301706
608	Nucleolar RNA helicase 2	Q9NR30	-0.168996274
609	ATP-binding cassette sub-family E member 1	P61221	-0.16881203
610	AP-5 complex subunit zeta-1	O43299	-0.168742005
611	26S proteasome non-ATPase regulatory subunit 11	O00231	-0.16855477
612	Probable ATP-dependent RNA helicase DDX56	Q9NY93	-0.16853634
613	WD repeat and FYVE domain-containing protein 1	Q8IWB7	-0.168529019
614	Peptidyl-prolyl cis-trans isomerase FKBP4	Q02790	-0.168371552
615	28S ribosomal protein S9, mitochondrial	P82933	-0.168020588
616	SWI/SNF-related matrix-associated actin-dependent regulator of chromatin subfamily A member 5	O60264	-0.167316269
617	Unconventional myosin-XIX	Q96H55	-0.167131303
618	Alpha-enolase	P06733	-0.167088355
619	Nuclear pore complex protein Nup85	Q9BW27	-0.166362119
620	DNA repair protein complementing XP-C cells	Q01831	-0.166351796
621	H/ACA ribonucleoprotein complex subunit DKC1	O60832	-0.165795828

622	Eukaryotic translation initiation factor 4 gamma 2	P78344	-0.165329553
623	Phosphoacetylglucosamine mutase	O95394	-0.16532845
624	Cullin-5	Q93034	-0.165257019
625	CAAX prenyl protease 1 homolog	O75844	-0.165202698
626	Protein NPAT	Q14207	-0.165198449
627	CDK5 regulatory subunit-associated protein 2	Q96SN8	-0.165080067
628	Peptidyl-prolyl cis-trans isomerase D	Q08752	-0.164897539
629	Vacuolar protein sorting-associated protein 51 homolog	Q9UID3	-0.164574928
630	V-type proton ATPase 116 kDa subunit a 3	Q13488	-0.164129425
631	Glycylpeptide N-tetradecanoyltransferase 1	P30419	-0.163790986
632	PDZ and LIM domain protein 7	Q9NR12	-0.163567322
633	60S ribosomal protein L5	P46777	-0.162805589
634	Tyrosine-protein kinase JAK1	P23458	-0.162434851
635	Alkaline phosphatase, tissue-nonspecific isozyme	P05186	-0.162379887
636	E3 ubiquitin-protein ligase RBBP6	Q7Z6E9	-0.162209232
637	DNA polymerase delta catalytic subunit	P28340	-0.162070713
638	Armadillo-like helical domain-containing protein 3	Q5T2E6	-0.16170892
639	Rap guanine nucleotide exchange factor 6	Q8TEU7	-0.161645876
640	Gamma-tubulin complex component 4	Q9UGJ1	-0.16089924
641	Integrin alpha-V	P06756	-0.160655767
642	Probable global transcription activator SNF2L1	P28370	-0.160212994
643	WD repeat-containing protein 36	Q8NI36	-0.159659927
644	Protein arginine N-methyltransferase 3	O60678	-0.159575669
645	Pachytene checkpoint protein 2 homolog	Q15645	-0.159536837
646	Ribosome biogenesis protein BOP1	Q14137	-0.159469677
647	U4/U6 small nuclear ribonucleoprotein Prp4	O43172	-0.158981646
648	Protein SDA1 homolog	Q9NVU7	-0.158968317
649	Liprin-beta-1	Q86W92	-0.158947972
650	U3 small nucleolar RNA-associated protein 6 homolog	Q9NYH9	-0.158836456
651	Oxysterol-binding protein-related protein 9	Q96SU4	-0.158801446
652	Ubiquitin conjugation factor E4 B	O95155	-0.158620735
653	60S ribosomal protein L7	P18124	-0.158325017

654	Dedicator of cytokinesis protein 7	Q96N67	-0.158027718
655	Coatomer subunit alpha	P53621	-0.156849847
656	Proteasome adapter and scaffold protein ECM29	Q5VYK3	-0.156611959
657	Src substrate cortactin	Q14247	-0.156224133
658	Protein timeless homolog	Q9UNS1	-0.155953015
659	Signal transducer and activator of transcription 3	P40763	-0.155916683
660	Optineurin	Q96CV9	-0.155858871
661	Proteasome activator complex subunit 2	Q9UL46	-0.155794906
662	Translocation protein SEC63 homolog	Q9UGP8	-0.155744261
663	COP9 signalosome complex subunit 2	P61201	-0.155728702
664	WD40 repeat-containing protein SMU1	Q2TAY7	-0.155689618
665	Ribosomal protein S6 kinase alpha-1	Q15418	-0.155681576
666	Utrophin	P46939	-0.155557153
667	Gasdermin-E	O60443	-0.1553998
668	HBS1-like protein	Q9Y450	-0.154907417
669	Mitochondrial chaperone BCS1	Q9Y276	-0.154803112
670	Autophagy-related protein 9A	Q7Z3C6	-0.154687622
671	Conserved oligomeric Golgi complex subunit 8	Q96MW5	-0.15450396
672	Phosphatidylinositol 3,4,5-trisphosphate 5-phosphatase 2	O15357	-0.15445551
673	Nucleobindin-1	Q02818	-0.154043511
674	Partitioning defective 3 homolog	Q8TEW0	-0.153995029
675	Peripheral plasma membrane protein CASK	O14936	-0.15397715
676	RNA-binding protein 26	Q5T8P6	-0.15352379
677	Synembryn-A	Q9NPQ8	-0.153303819
678	DENN domain-containing protein 4C	Q5VZ89	-0.15292382
679	E3 ubiquitin-protein ligase UBR1	Q8IWV7	-0.152586445
680	Echinoderm microtubule-associated protein-like 2	O95834	-0.152179931
681	Copine-8	Q86YQ8	-0.152169984
682	Sodium/potassium-transporting ATPase subunit alpha-1	P05023	-0.152125245

Reference for supporting information.

[1] M. Yasuda, Y. Ma, S. Okabe, Y. Wakabayashi, D. Su, Y. Chang, H. Seimiya, M. Tera, K. Nagasawa, *Chem. Commun*., **2020**, *56*, 12905.
